# Photoredox Iron-Catalyzed Decarboxylative Radical Cyclization for the Synthesis of Oxindoles and Chroman-4-ones

**DOI:** 10.1021/acs.joc.5c00669

**Published:** 2025-05-15

**Authors:** Shaoyang Han, Litao Liu, Jianqing Meng, Meichao Li, Qun Cao, Zhenlu Shen

**Affiliations:** † College of Chemical Engineering, 12624 Zhejiang University of Technology, Hangzhou 310014, China; ‡ State Key Laboratory of Advanced Separation Membrane Materials, Zhejiang University of Technology, Hangzhou 310014, China; § School of Chemistry, 4488 University of Leicester, Leicester LE1 7RH, U.K.

## Abstract

A sustainable, photocatalytic approach for the synthesis of oxindoles and chroman-4-ones was developed using carboxylate salts as radical precursors and FeCl_3_ as a catalyst. The reaction proceeds via a decarboxylative radical cyclization mechanism triggered by ligand-to-metal charge transfer under visible light irradiation, operating efficiently at room temperature. This method demonstrates excellent substrate scope, including the use of various alkyl carboxylates, and functional group tolerance and offers a scalable pathway for gram-scale synthesis, highlighting its practical application.

## Graphical abstract

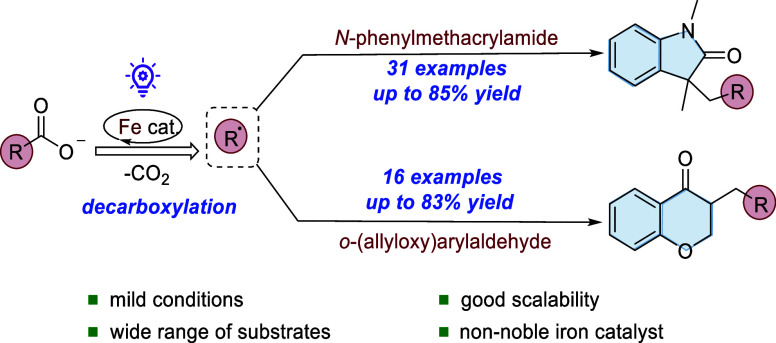

## Introduction

Oxindole and chroman-4-one scaffolds serve as the core structure for a wide range of natural products, medicinal compounds, and biological compounds, exhibiting extraordinary biological and pharmaceutical activities (Figure 1).^1^ Therefore, significant efforts have been directed toward the synthesis of these scaffolds. Traditional oxindole synthesis requires noble transition metal (e.g., Pd, Rh)-catalyzed coupling of arylamides with alkyl (pseudo)-halides, which necessitates high temperatures and expensive catalysts/ligands.^2^ Moreover, the well-known traditional syntheses of chroman-4-ones, such as Luthman^3^ and Stetter^4^ reactions, require multistep procedures under harsh reaction conditions.

**Figure 1:**
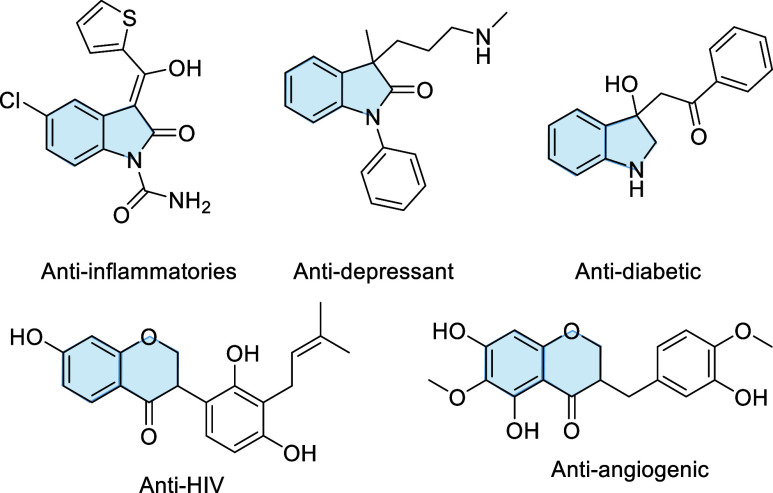
Examples of bioactive oxindoles and chroman-4-ones.

Among various recently developed synthetic methodologies, the intermolecular cascade radical cyclization of alkenes has emerged as one of the most straightforward approaches for constructing oxindoles and chroman-4-ones. For example, it was reported that precursors such as aldehydes,^5^ alkanes,^6^ *N*-(acyloxy)­phthalimides,^7^ and hypervalent iodines^8^ can generate carbon radicals, which could subsequently participate in cascade radical cyclization for the synthesis of oxindoles. Meanwhile, it was also reported that 1,3-dicarbonyl compounds,^9^ acryloyl chlorides,^10^ oxamic acids,^11^ cyclopropanols,^12^ hypervalent iodines,^13^ and difluoromethylated bromides^14^ could also be used as radical precursors for the synthesis of chroman-4-ones derivatives. However, many of these methods relied on expensive noble metal catalysts (e.g., Ir, Re, Ag, and Pd), elevated temperatures, and building blocks that necessitate multistep preparation processes.

Carboxylic acids are abundant, cost-effective industrial raw materials commonly used for the synthesis of pharmaceutical intermediates.^15^ Recently, research on decarboxylative functionalization using carboxylic acids has led to continuous innovative breakthroughs in organic synthesis.^16^ In particular, decarboxylative cascade cyclization reactions have emerged as a promising strategy for the green synthesis of heterocyclic compounds due to their exceptional atom economy.^17^ However, the generation of carbon radicals from the corresponding organic carboxylic acids has encountered significant challenges due to the high oxidation potential of the carboxylate anions.^18^ To the best of our knowledge, the existing methods that utilize inexpensive carboxylic acid derivatives as alkyl radical sources for the synthesis of oxindole and chroman-4-one scaffolds still heavily rely on the use of precious metals catalysts or photocatalysts (e.g., AgNO_3_, Ir-based complexes, CeCl_3_, 2-chlorothioxanthone, methylene blue); for details, see Scheme 1a,b.^19^ This reliance not only increases the overall cost but also limits the sustainability of the process. Therefore, the development of a practical and sustainable protocol to utilize alkyl carboxylic derivatives as radical sources for the efficient synthesis of oxindoles and chroman-4-ones remains highly desirable.

**Scheme 1:**
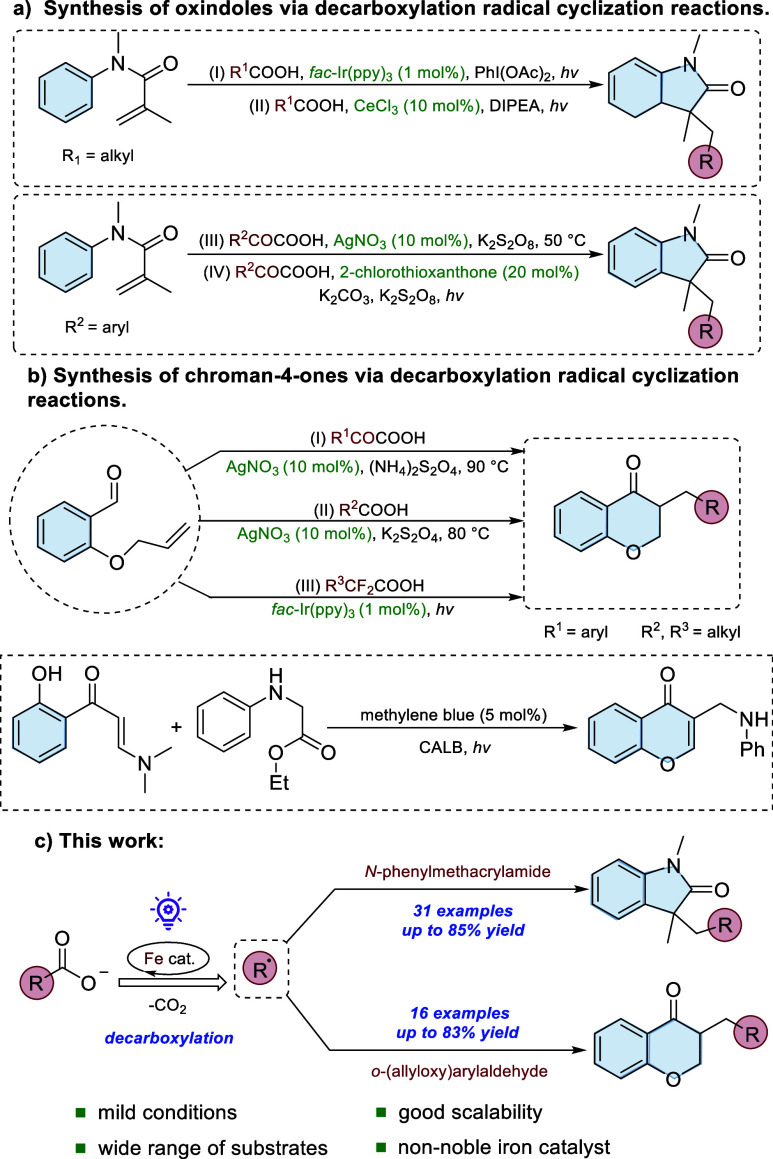
Synthetic Strategy

In the ongoing pursuit of greener and more sustainable synthetic methodologies, light-triggered ligand-to-metal charge transfer (LMCT) has emerged as an efficient and powerful tool, capable of selectively generating carbon-centered radicals through C–H bond and C–heteroatom bond activation.^20^ Recently, it was discovered that Fe­(III)-carboxylate complexes, formed in situ from carboxylate anions and trivalent iron salts, could undergo an intramolecular LMCT process under visible light irradiation, leading to the generation of carbon radicals and Fe­(II) species via a subsequent decarboxylation process.^21^

Inspired by these studies, herein, we report a novel and general approach for the sustainable photocatalytic synthesis of oxindole and chroman-4-one derivatives using carboxylate salts as radical precursors, with FeCl_3_ as a catalyst. This transformation proceeds via a decarboxylative radical cyclization pathways, leveraging radicals generated through ligand-to-iron charge transfer. The reaction operates efficiently under mild conditions at room temperature, demonstrating excellent substrate scope and functional group tolerance. Notably, the method is compatible with a wide range of tertiary, secondary, and primary alkyl carboxylates, showcasing scalability to gram-scale synthesis, highlighting its practicality for scale synthesis.

## Results and Discussion

We initiated our study by employing *N*-methyl-*N*-acryloylphenylamine **1a** (0.3 mmol) as a model substrate in the presence of sodium pivalate **2a** (1.5 mmol, 5 equiv) in acetonitrile solvent (MeCN, 5.0 mL). Iron­(III) chloride (FeCl_3_, 10 mol %) was used as catalyst, while tetrabutylammonium bromide (TBAB, 5 mol %) served as a phase transfer catalyst. The reaction mixture was irradiated with a 35 W blue LED light at 390 nm under a nitrogen atmosphere with di-*tert*-butyl peroxide (DTBP) as an oxidant for 26 h at room temperature, affording the desired 1,3-dimethyl-3-neopentylindole-2-one (**3aa**) with a yield of 75% (Table 1, entry 1). Control experiments showed that the exclusion of TBAB would lead to a slightly decreased yield of **3aa** (Table 1, entry 2). In the absence of FeCl_3_, no product formation was observed (Table 1, entry 3). Substituting FeCl_3_ with alternative iron catalysts (e.g., FeCl_2_, FeCl_2_·4H_2_O, Fe­(acac)_3_, Fe_2_(SO_4_)_3_, FeSO_4_·7H_2_O) resulted in a reduced yield of **3aa** (Table 1, entries 4–8). These experimental findings highlighted the crucial role of FeCl_3_ in the reaction process. The absence of light resulted in no product formation (Table 1, entry 9), while any deviation from the 390 nm wavelength, either by increasing or decreasing it, resulted in a decline in product yield (Table 1, entries 10 and 11). This observation aligns with the reported literature, which indicates that iron carboxylate complexes primarily absorb light in the near-ultraviolet region.^22^ Control experiments also demonstrated that the reaction did not proceed in the absence of an oxidizing agent, indicating that an oxidant is essential for the transformation (Table 1, entry 12). Several oxidizing agents, including potassium persulfate (K_2_S_2_O_8_), manganese dioxide (MnO_2_), sodium chlorate (NaClO_3_), sodium iodate (NaIO_3_), dicumyl peroxide (DCP), and air, were evaluated (Table 1, entries 13–18), but none exhibited superior efficiency compared to di-*tert*-butyl peroxide (DTBP). Further screening of various solvents, including MeCN/H_2_O mixtures, dichloroethane (DCE), dichloromethane (DCM), chlorobenzene, toluene, and tetrahydrofuran (THF), did not lead to significant improvements in yield (Table 1, entries 19–24). Finally, we investigated the effect of the carboxylate salt loading and reaction time (see Tables S1 and S2 in the Supporting Information for details). It was determined that 5 equiv of the carboxylate salt and a reaction duration of 26 h were optimal for maximizing the yield.

**Table 1:**
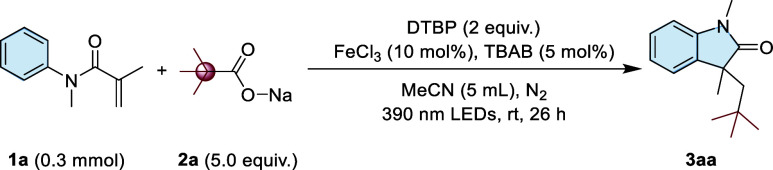
Optimization of the Reaction Conditions^a^

Entry	Deviations from standard conditions	Yield (%)^b^
1	none	75
2	no TBAB	70
3	no iron catalyst	N.R.
4	FeCl_2_	65
5	FeCl_2_·4H_2_O	15
6	Fe(acac)_3_	30
7	Fe_2_(SO_4_)_3_	25
8	FeSO_4_·7H_2_O	20
9	no light	N.R.
10	380 nm LED	54
11	405 nm LED	68
12	no oxidant	N.R.
13	K_2_S_2_O_8_ as an oxidant	20
14	MnO_2_ as an oxidant	16
15	NaClO_3_ as an oxidant	20
16	NaIO_3_ as an oxidant	25
17	DCP as an oxidant	43
18	air as an oxidant	trace
19	MeCN/H_2_O (4:1)	16
20	DCE	25
21	DCM	50
22	chlorobenzene	trace
23	toluene	trace
24	THF	trace

a Reaction condition: **1a** (0.3 mmol), **2a** (1.5 mmol), FeCl_3_ (10 mol %), TBAB (5 mol %), DTBP (2 equiv), MeCN (5 mL), N_2_, 390 nm LEDs, 26 h.

b Isolated yields based on **1a**.

Under the optimized conditions, we conducted a comprehensive investigation of the substrate scope, involving various *N*-methyl-*N*-aryl acrylamides (**1**) and **2a**. As shown in Scheme 2, *N*-aryl acrylamides bearing electron-donating (−CH_3_, –OCH_3_) or electron-withdrawing groups (−F, −Br, −Cl, and −COOEt) at the ortho, meta, and para positions underwent smooth conversion with **2a**, affording the corresponding indolinone derivatives in moderate to very good yields (**3ba**–**3sa**). Interestingly, a meta-methyl substituent resulted in the formation of two products (**3pa and 3pa′**), whereas meta-substituents such as −OCH_3_, −Cl, or −Br led to the exclusive formation of a single product (**3qa**, **3ra**, and **3sa**). However, the highly electron-withdrawing nitro group (-NO_2_) hardly participates in the reaction (**3na**). Next, various *N* substituted *N*-aryl acrylamides (e.g., *N*-ethyl, *N*-phenyl, and *N*-benzyl) were investigated under optimized reaction conditions, yielding the desired products in 71–78% yield. However, when *N*-acetyl-*N*-aryl acrylamide was used as the substrate, no targeted product (**3wa**) was detected.

**Scheme 2:**
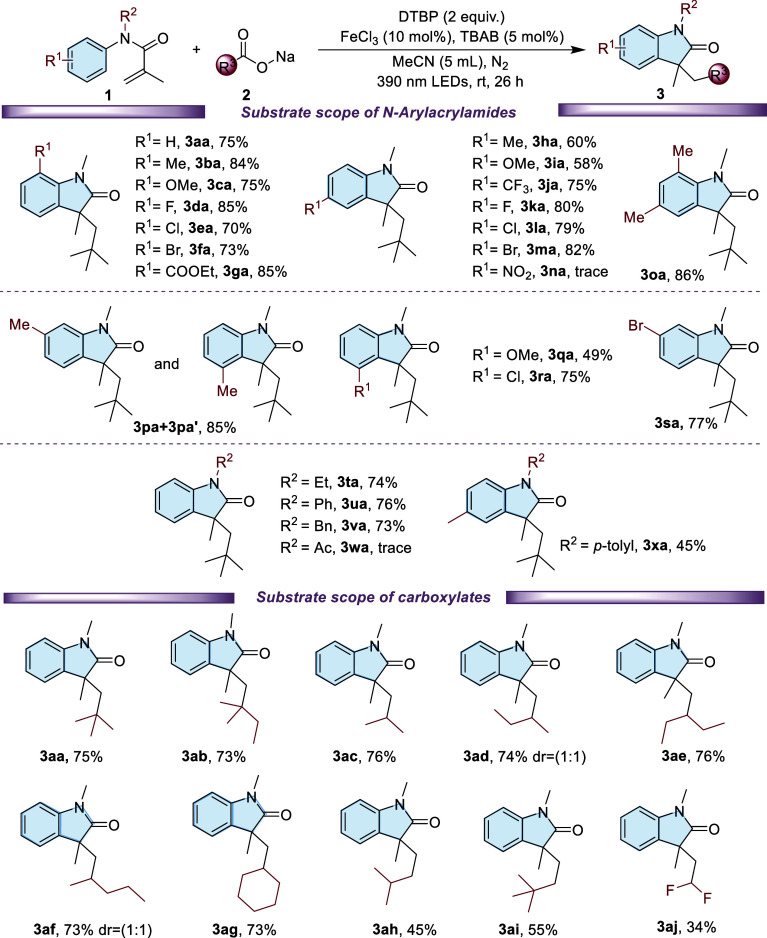
Substrate Scopes of *N*-Arylacrylamides and Carboxylates^a^

a Reaction condition: **1** (0.3 mmol), **2** (1.5 mmol), FeCl_3_ (10 mol %), TBAB (5 mol %), DTBP (2 equiv), MeCN (5 mL), N_2_, 390 nm LEDs, 26 h.

We also explored the reaction of **1a** with various carboxylate sodium salts under the optimized reaction conditions. As shown in Scheme 2, both tertiary and secondary carboxylate sodium salts proved to be effective substrates, affording the corresponding products (**3ab**, **3ac**, **3ad**, **3ae**, **3af**, **and 3ag**) in good yields (73–76%). Notably, when the substrate was sodium difluoroacetate, we obtained the target product **3aj** in an unsatisfactory 34% isolated yield. It was noticed that when primary carboxylate sodium salts were used, the yields of corresponding indolinones (**3ah**, **3ai**) decreased slightly (45–55%). These findings suggest that the stability of the radical intermediate formed after decarboxylation plays a crucial role in determining the efficiency of the reaction—more stable radicals lead to higher yields.

With the successful development of photoredox decarboxylative radical cyclization for the synthesis of oxindoles, this method was further explored for the synthesis of chroman-4-one derivatives. Initially, applying the optimized reaction conditions from Table 1 to 2-(allyloxy)­benzaldehyde (**4a**) resulted in a sluggish reaction, affording only a 40% yield of **6aa** (Table S3, entry 2). Thus, several modifications to the original method were implemented to optimize the synthesis of chroman-4-one derivatives, including an increase in the amount of carboxylate reagent (**2a**, Table S3) or replacing **2a** with carboxylic acid (**5a**, Table S4). It was found that the use of in situ generated carboxylate salt, by mixing carboxylic acid with an organic base (e.g., NEt_3_, DBU), could significantly improve the yield of **6aa** (Table S5, entries 1–2). This improvement could be attributed to the increased solubility of the in situ generated carboxylate salt with the organic cation, compared to that of **2a**, eliminating the need for phase transfer catalyst TBAB. Furthermore, increasing the amount of DTBP from 2 equiv. to 4 equiv. and extending the reaction time to 32 h led to a satisfactory yield of **6aa** at 82% (for more detailed optimization, see Tables S3–S9 in the Supporting Information).

Once the optimized reaction conditions for the synthesis of chroman-4-ones were established, the substrate scope was explored using a variety of substituted *o*-(allyloxy)­arylaldehydes and alkyl carboxylic acids (Scheme 3). It was found that *o*-(allyloxy)­arylaldehydes, which contain either electron-donating or electron-withdrawing substituents, such as –CH_3_, −*t*-Bu, –OCH_3_, −Br, −Cl, and −F, underwent smooth reactions with pivalate acid **5a**, resulting in the formation of the corresponding chroman-4-one derivatives **6ba–6ka** with moderate to good yields, ranging from 54% to 83%. In general, *o*-(allyloxy)­arylaldehydes bearing electron-deficient substituents on the aryl group demonstrate greater reactivity than their electron-rich counterparts. It was proposed that the electron-withdrawing groups reduce the electron density of the aldehyde moiety through an inductive effect, thereby facilitating the occurrence of nucleophilic carbon-centered radical cyclization steps (for more details, see the proposed mechanism part and Scheme 5). Subsequently, a range of aliphatic tertiary and secondary carboxylic acids were used to expand the substrate scope of this method, leading to the corresponding chroman-4-ones in yields between 50 and 75% (**6ab**–**6af**). Similar to the reactivity trend observed in the synthesis of oxindoles (Scheme 3), tertiary carboxylic acids demonstrated better reactivity than secondary ones in the formation of chroman-4-ones.

**Scheme 3:**
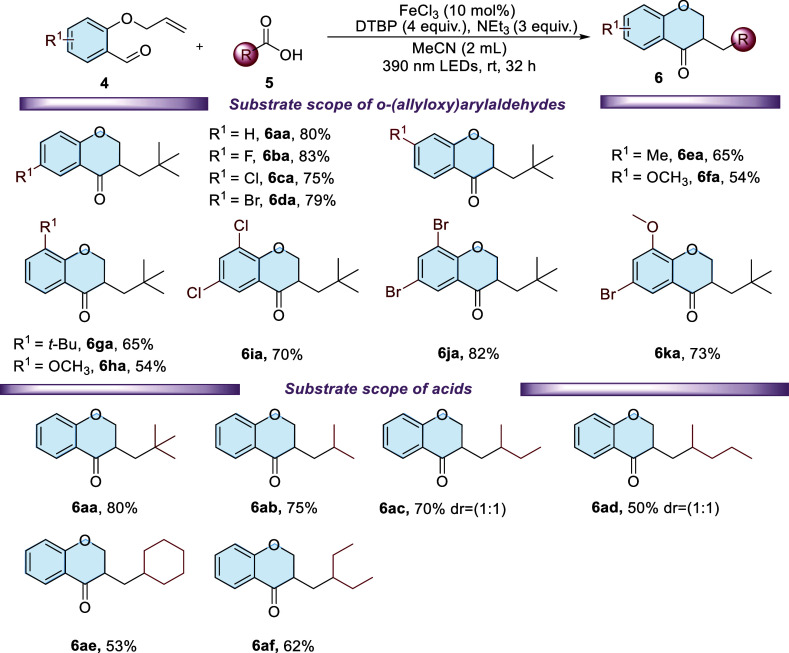
Substrate Scopes of *o*-(Allyloxy)­arylaldehydes and Acids^a^

a Reaction condition: **3** (0.3 mmol), **4** (1.5 mmol), FeCl_3_ (10 mol %), NEt_3_ (5 mol %), DTBP (4 equiv), MeCN (2 mL), N_2_, 390 nm LEDs, 32 h.

To further establish the scalability of our method, large-scale reactions were performed using 6 mmol **1a** and **4a** for the synthesis of **3aa** and **6aa**. The desired products (**3aa** and **6aa**) were obtained in near-gram scale with yields of 66% and 73%, respectively (Scheme 4). Although there was a slight decrease in the yield compared to that by the small-scale synthesis (**5aa**—75% and **6aa**—80% in Schemes 2 and 3), the gram-scale synthesis demonstrated the scalability of the reaction system.

**Scheme 4:**
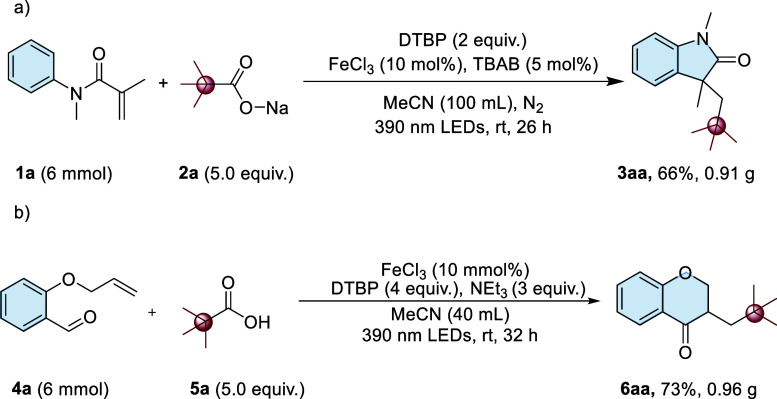
Gram-Scale Synthesis

To further investigate the mechanism of this photochemical iron-catalyzed decarboxylative radical cyclization, several control experiments were conducted with **1a** and **2a** as model substrates. As shown in Scheme 5a, when radical scavenger TEMPO (2,2,6,6-tetramethylpiperidine-1-oxyl) was added, the transformation was completely suppressed. The HRMS analysis of the reaction mixture confirmed the formation of the *tert*-butyl radical during the photoreaction, as evidenced by the detection of compound **7**, an adduct of TEMPO and the *tert*-butyl radical [M + H^+^] found 214.2170, calculated 214.2165; for detailed HRMS, see Figure S1. Additionally, when a stoichiometric amount of FeCl_3_ (5 equiv) was added in the absence of the DTBP oxidant, no target product was detected. This experimental result confirms that DTBP plays an important role in the formation of the final product. In addition, the need for continuous visible light irradiation in this decarboxylative cascade alkylation/cyclization was demonstrated through the on/off light experiment (see Section S7 in the Supporting Information).

**Scheme 5:**
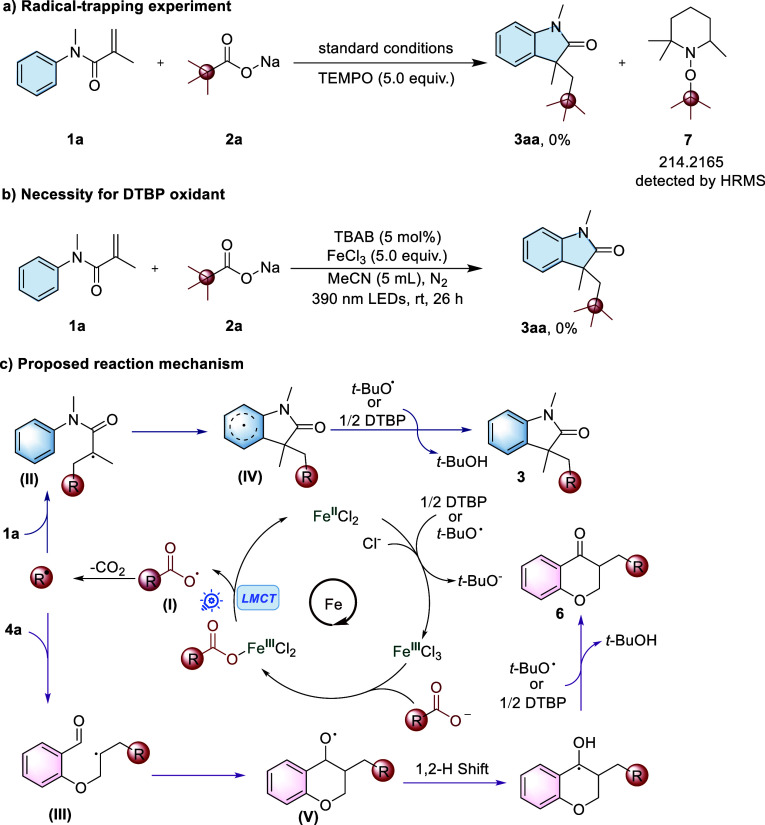
Control Experiments and Proposed Mechanism

Based on the above obtained results and existing literature studies,^23^ a plausible reaction mechanism was proposed (Scheme 5). Initially, Fe­(III)-carboxylate complexes, which are generated in situ from carboxylate anions and Fe­(III) cations, can undergo ligand-to-metal charge transfer (LMCT) under UV light irradiation, leading to the formation of the acyloxy radical intermediate (**I**) and Fe­(II) species (for UV–vis absorbance spectra, see Figures S4 and S5 in the Supporting Information).^24^ The Fe­(II) species can then be reoxidized by oxidants (e.g., DTBP or in situ generated *t*-BuO^•^) to regenerate Fe­(III) species,^25^ while intermediate (**I**) rapidly undergoes decarboxylation, generating a carbon-centered radical (R^•^), which subsequently participates in an intermolecular addition to the alkene substrate (**1a** or **4a**), leading to the formation of carbon-centered intermediates (**II**) or (**III**). These intermediates can undergo rapid intramolecular radical cyclization to produce a benzenoid π intermediate (**IV**) or alkoxy radical intermediate (**V**). Finally, oxidative rearomatization by DTBP or *t*-BuO^•^ facilitates the conversion of intermediates **IV** or **V** into the desired product **3** or **6**.

In conclusion, we developed a novel and efficient method for the sustainable photocatalytic synthesis of oxindole and chroman-4-one derivatives, utilizing carboxylate salts as a radical precursor and FeCl_3_ as a catalyst via a decarboxylative radical cyclization reaction. This method offers several advantages, including excellent substrate scope, functional group tolerance, and the ability to work with tertiary, secondary, and primary alkyl carboxylates under mild room temperature conditions. The scalability of this approach was also demonstrated, highlighting its potential for large-scale synthesis. Ongoing studies in our laboratory are focused on expanding the scope of this photocatalytic LMCT method for other synthetic applications in the field of *N*-heterocycle synthesis.

## Experimental Section

### Safety Statement

Caution! Although DTBP is a stable organic peroxide, the reaction with DTBP should be quenched with sodium sulfite and water before workup procedure. Caution! Triethylamine and carboxylic acids have irritating odors, and their inhalation can cause damage to the body. When you work with these chemicals, weighing and transferring operations should be carried out within a fume hood. Prolonged or repeated exposure to these compounds may result in adverse health effects. Additionally, exposure to light sources can be harmful to the eyes, so protective goggles should be worn.

## Supplementary Material



## Data Availability

The data underlying this study are available in the published article and its Supporting Information.

## Notes

General Information, reaction optimization, general procedure for the synthesis of oxindoles and chroman-4-ones, additional control experiment, UV–vis absorbance experiment, HRMS spectra, and NMR spectra (PDF)
