# Supplementary material for: Photoredox Iron-Catalyzed Decarboxylative Radical Cyclization for the Synthesis of Oxindoles and Chroman-4-ones
Source: J Org Chem. 2025 May 15;90(21):7062–9. doi: 10.1021/acs.joc.5c00669 (PMC12131215; doi:10.1021/acs.joc.5c00669)
Supplement: Supplementary file 1 [file jo5c00669_si_001.pdf]

Supporting Information

Photoredox Iron-Catalyzed Decarboxylative Radical Cyclization for the Synthesis of Oxindoles and Chroman-4-ones

Shaoyang Han,[a,b] Litao Liu,[a] Jianqing Meng,[a] Meichao Li,*[a] Qun Cao,*[c] and Zhenlu Shen*[a,b]

[a] College of Chemical Engineering, Zhejiang University of Technology, Hangzhou 310014, China

[b] State Key Laboratory of Advanced Separation Membrane Materials, Zhejiang University of Technology, Hangzhou 310014, China

[c] School of Chemistry, University of Leicester, Leicester, LE1 7RH, United Kingdom

Email: limc@zjut.edu.cn
Email: qc52@le.ac.uk
Email: zhenlushen@zjut.edu.cn

1. General Information S3

2. Reaction Optimization S4

Table S1. Influence of sodium pivalate loading on the synthesis of oxindoles S4

Table S2. Effect of reaction time on the synthesis of oxindoles S4

Table S3. Influence of sodium pivalate loading on the synthesis of chroman-4-ones S5

Table S4. Screening of pivalate acid loading for the synthesis of chroman-4-ones S5

Table S5. Effect of bases on the synthesis of chroman-4-ones S5

Table S6. Screening of the loading of NEt_3 for the synthesis of chroman-4-ones S6

Table S7. Screening of oxidants for the synthesis of chroman-4-ones S6

Table S8. Screening of the loading of the DTBP S6

Table S9. Screening of reaction time S7

3. General procedure A: for the synthesis of oxindoles S8

4. General procedure B: for the radical cascade cyclization of *o*-(allyloxy)arylaldehydes with acids S9

5. Gram-scale synthesis for oxindoles and chroman-4-ones S10

6. Control Experiment S12

7. "On/off" LED irradiation experiment S13

8. UV-vis absorbance experiment S15

9. Characterization of products S17

10. References S45

11. NMR Spectra S46

12. HRMS Spectra S95

1. General Information

The reagents and solvents were purchased from commercial suppliers and used without further purification unless noted. All reactions were monitored by TLC with silica gel-coated plates. Product purification was accomplished by flash chromatography using 200-300 mesh silica gel. The ^{1}H NMR (400 MHz), ^{13}C NMR (101 MHz), and ^{19}F NMR (376 MHz) spectra were recorded on a Varian spectrometer using $CDCl_3$ or DMSO-d_6 as the solvent. Measurements were done at ambient temperature. ^{1}H NMR chemical shifts are referenced to the residual hydrogen signals of the deuterated solvent (7.26 ppm for $CDCl_3$ or 2.50 ppm for DMSO-d_6). The ^{13}C NMR chemical shifts are referenced to the ^{13}C signals of the deuterated solvent (77.16 ppm for $CDCl_3$ or 39.50 ppm for DMSO-d_6). Abbreviations used in the description of NMR data are listed as follows: s = singlet, d = doublet, dd = doublet of doublet, t = triplet, m = multiplet. Mass spectra were measured with a HRMS-APCI instrument using ESI ionization. Mass spectra were measured with an HRMS-APCI instrument using ESI ionization. Melting points were measured by WRS-1C Melting Point Apparatus (Shanghai INESA Physico-Optical Instrument Co., Ltd.). Photo-induced reactions were performed under 390 nm light irradiation using a 35 W LED lamp purchased from Xuzhou Ai Jia Electronic Technology Co., Ltd. (available on Taobao.com). The distance from the light source to the irradiation vessel was approximately 1.5 cm, and no filter was used in our study. A fan was employed to ensure that the reactions remained at or near room temperature when using the LED.

2. Reaction Optimization

Table S1. Influence of sodium pivalate loading on the synthesis of oxindoles

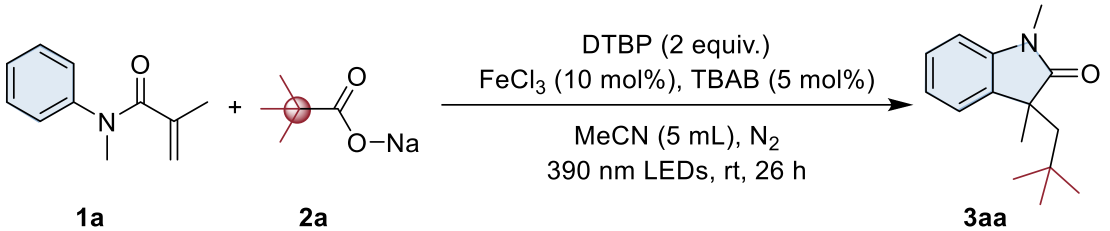

1a **2a** **3aa**

Entry	**x (mmol)**	Yield of **3aa** (%)
1	1.20	56
2	1.35	69
**3**	**1.50**	**75**
4	1.65	74

Reaction condition: **1a** (0.3 mmol, 52.6 mg), **2a** (**x** mmol), $FeCl_3$ (0.03 mmol, 4.9 mg), TBAB (0.015 mmol, 4.8 mg), DTBP (0.6 mmol, 87.7mg), MeCN (0.06 M, 5 mL), N_2, 390 nm LEDs, 26 h. Isolated yields based on **1a**.

Table S2. Effect of reaction time on the synthesis of oxindoles

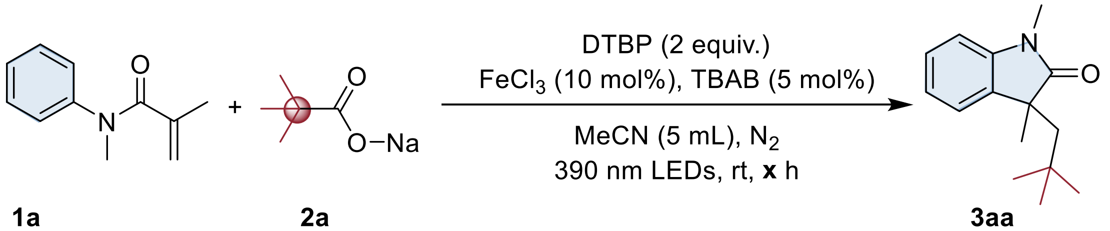

1a **2a** **3aa**

Entry	x (h)	Yield of **3aa** (%)
1	8	30
2	16	54
3	24	68
**4**	**26**	**75**
5	28	74

Reaction condition: **1a** (0.3 mmol, 52.6 mg), **2a** (**1.5** mmol, 0.1863 g), $FeCl_3$ (0.03 mmol, 4.9 mg), TBAB (0.015 mmol, 4.8 mg), DTBP (0.6 mmol, 87.7 mg), MeCN (0.06 M, 5 mL), N_2, 390 nm LEDs, **x** h. Isolated yields based on **1a**.

Table S3. Influence of sodium pivalate loading on the synthesis of chroman-4-ones

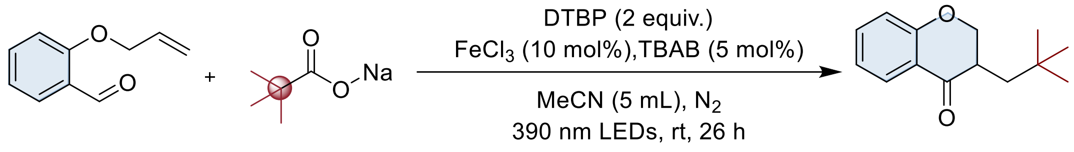

Entry	**x (mmol)**	Yield of **6aa** (%)
1	0.9	33
2	1.5	40
**3**	**2.1**	**46**

Reaction condition: **4a** (0.3 mmol, 48.7 mg), **2a** (**x** mmol), $FeCl_3$ (0.03 mmol, 4.9 mg), TBAB (0.015 mmol, 4.8 mg), DTBP (0.6 mmol, 87.7 mg), MeCN (0.06 M, 5 mL), N_2, 390 nm LEDs, **26** h. Isolated yields based on **4a**.

Table S4. Screening of pivalate acid loading for the synthesis of chroman-4-ones

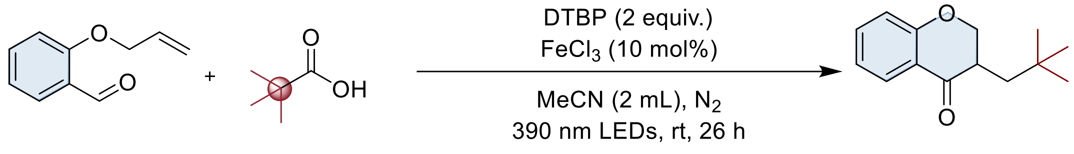

Entry	**x (mmol)**	Yield of **6aa** (%)
1	1.2	43
**2**	**1.5**	**50**
3	1.8	47

Reaction condition: **4a** (0.3 mmol, 48.7 mg), **5a** (**x** mmol), $FeCl_3$ (0.03 mmol, 4.9 mg), DTBP (0.6 mmol, 87.7 mg), MeCN (0.15 M, 2 mL), N_2, 390 nm LEDs, **26** h. Isolated yields based on **4a**.

Table S5. Effect of bases on the synthesis of chroman-4-ones

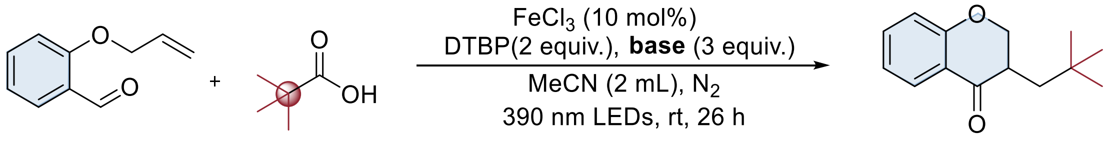

Entry	Base	Yield of **6aa**
**1**	**TEA**	**59**
2	DBU	54

Reaction conditions: **4a** (0.3 mmol, 48.7 mg), **5a** (1.5 mmol, 0.1532 g), $FeCl_3$ (0.03 mmol, 4.9 mg), DTBP (0.6 mmol, 87.7 mg), base as specified (0.9 mmol, 3 equiv.), MeCN (0.15 M, 2 mL), N_2, 390 nm LEDs, 26 h. Isolated yields based on **4a**.

Table S6. Screening of the loading of NEt_3 for the synthesis of chroman-4-ones

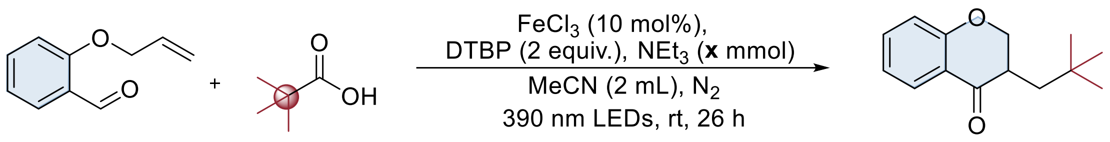

4a **5a** **6aa**

Entry	**x (mmol)**	Yield of **6aa**
1	0.6	45
**2**	**0.9**	**59**
3	1.2	43

Reaction conditions: **4a** (0.3 mmol, 48.7 mg), **5a** (1.5 mmol, 0.1532 g), $FeCl_3$ (0.03 mmol, 4.9 mg), DTBP (0.6 mmol, 87.7 mg), NEt_3 (**x** mmol), MeCN (0.15 M, 2 mL), N_2, 390 nm LEDs, 26 h. Isolated yields based on **4a**.

Table S7. Screening of oxidants for the synthesis of chroman-4-ones

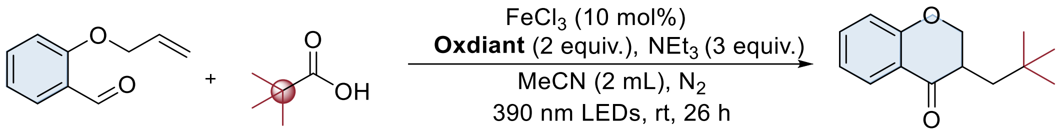

4a **5a** **6aa**

Entry	Oxidant	Yield of **6aa**
**1**	**DTBP**	**59**
2	DCP	47
3	$K_2S_2O_8$	35

Reaction conditions: **4a** (0.3 mmol, 48.7 mg), **5a** (1.5 mmol, 0.1532 g), $FeCl_3$ (0.03 mmol, 4.9 mg), oxidant as specified (0.4 mmol, 2 equiv.), NEt_3 (0.9 mmol, 91.1 mg), MeCN (0.15 M, 2 mL), N_2, 390 nm LEDs, 26 h. Isolated yields based on **4a**.

Table S8. Screening of the loading of the DTBP

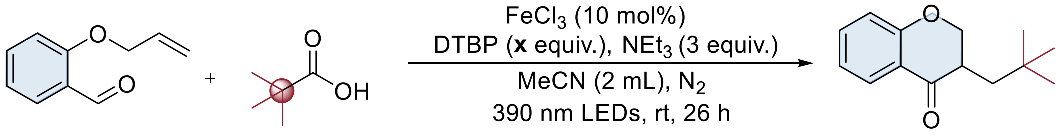

4a **5a** **6aa**

Entry	**mmol**	Yield of **6aa**
1	0.6	59
2	0.9	64
**3**	**1.2**	**73**
4	1.5	71

Reaction conditions: **4a** (0.3 mmol, 48.7 mg), **5a** (1.5 mmol, 0.1532 g), $FeCl_3$ (0.03 mmol, 4.9 mg), DTBP (**x** equiv.), NEt_3 (0.9 mmol, 91.1 mg), MeCN (0.15 M, 2 mL), N_2, 390 nm LEDs, 26 h. Isolated yields based on **4a**.

Table S9. Screening of reaction time

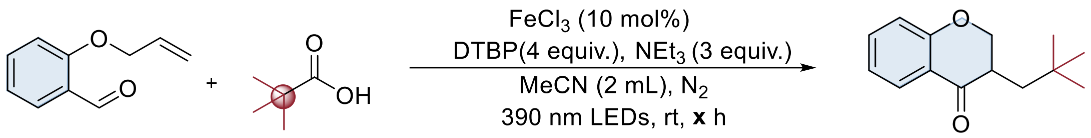

4a 5a 6aa

Entry	x	Yield of **6aa**
1	20	66
2	26	73
**3**	**32**	**82**
4	38	80

Reaction conditions: **4a** (0.3 mmol, 48.7 mg), **5a** (1.5 mmol, 0.1532 g), $FeCl_3$ (0.03 mmol, 4.9 mg), DTBP (1.2 mmol, 0.1743 g), NEt_3 (0.9 mmol, 91.01 mg), MeCN (0.15 M, 2 mL), N_2, 390 nm LEDs, reaction time as specified. Isolated yields based on **4a**.

3. General procedure A: for the synthesis of oxindoles

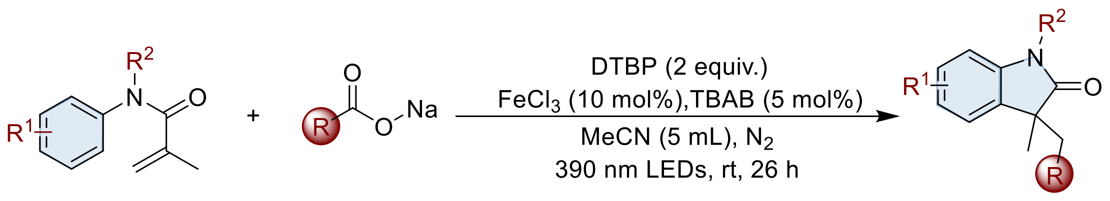

1 **2** **3**

An oven dried 10 mL reaction vial was charged with **1** (0.3 mmol, 1 equiv.), **2** (1.5 mmol, 5 equiv.), $FeCl_3$ (0.03 mmol, 4.9 mg), TBAB (0.015 mmol, 4.8 mg), DTBP (0.6 mmol, 87.74 mg) and a stir bar. Then MeCN (0.06 M, 5 mL) was added to the glass vial. The reaction vial was sealed, evacuated and backfilled three times with 1 atm of N_2. The reaction mixture was stirred and irradiated using a 35 W, 390 nm LED lamp for 26 hours until the reaction was complete (monitored by TLC). After the reaction, the mixture was quenched by the addition of H_2O (5 mL). EtOAc (5 mL) was then added, and the layers were separated. The aqueous layer was extracted with EtOAc (2 × 5 mL). The combined organic layers were dried over Na_2SO_4, filtered, and concentrated in vacuo. The solvent was removed by rotary evaporation, and the product was purified by column chromatography on silica gel using a petroleum ether/ethyl acetate mixture as the eluent.

4. General procedure B: for the radical cascade cyclization of *o*-(allyloxy)arylaldehydes with acids

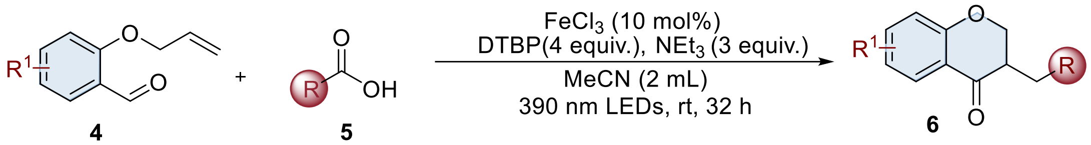

An oven dried 10 mL reaction vial was charged with **4** (0.3 mmol, 1 equiv.), **5** (1.5 mmol, 5 equiv.), $FeCl_3$ (0.03 mmol, 4.9 mg), DTBP (1.2 mmol, 0.1743 g) and a stir bar. Then MeCN (0.15 M, 2 mL) and NEt_3 (0.9 mmol, 91.1 mg) were added to the glass vial. The reaction vial was sealed, evacuated and backfilled three times with 1 atm of N_2. The reaction mixture was stirred and irradiated using a 35 W, 390 nm LED lamp for 32 hours until the reaction was complete (monitored by TLC). After reaction, the mixture was quenched by addition of $NaHCO_3$ (sat. aq. 5 mL). EtOAc (5 mL) was added and the layers were separated. The aqueous layer was extracted with EtOAc (2 x 5 mL). The combined organic layers were dried (Na_2SO_4), filtered and concentrated in vacuo. The solvent was removed by rotary evaporation and purified by column chromatography on silica gel using petroleum ether/ethyl acetate as the eluent.

5. Gram-scale synthesis for oxindoles and chroman-4-ones

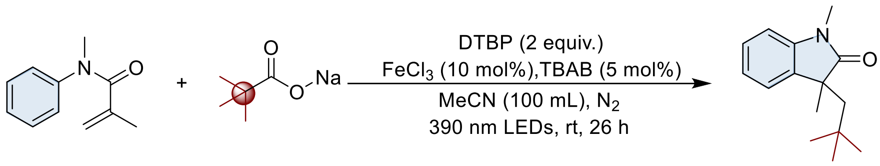

1a 2a 3aa

A 150 mL dried round-bottom flask was charged with a magnetic stirring bar, **1a** (6 mmol, 1.05 g), **2a** (30 mmol, 3.72 g), $FeCl_3$ (0.6 mmol, 97.3 mg) ,TBAB (0.3 mmol, 96.7 mg), DTBP (12 mmol, 1.75 g) and MeCN (0.06 M, 100 mL). The resulting mixture was stirred under 390 nm light-irradiation (LEDs) at room temperature with N_2 atmosphere for 26 hours. After reaction, the mixture was quenched by addition of $NaHCO_3$ (sat. aq. 30 mL). EtOAc (30 mL) was added and the layers were separated. The aqueous layer was extracted with EtOAc (2 x 30 mL). The combined organic layers were dried (Na_2SO_4), filtered and concentrated in vacuo. The crude product was purified by column chromatography. Compound **3aa** was isolated in 66% yield (0.91 g) as a yellow oil.

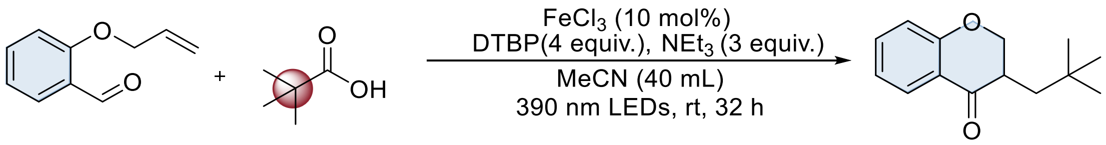

4a 5a 6aa

An oven dried 100 mL reaction vial was charged with **4a** (6 mmol, 0.97 g), **5a** (30 mmol, 3.06 g), $FeCl_3$ (0.6 mmol), DTBP (3.51 g, 4 equiv.) and a stir bar. Then MeCN (40 mL) and NEt_3 (1.82 g, 3 equiv.) were added to the glass vial. The reaction vial was sealed, evacuated and backfilled three times with 1 atm of N_2. The reaction mixture was stirred and irradiated using a 35 W, 390 nm LED lamp for 32 hours until the reaction was complete (monitored by TLC). After reaction, the mixture was quenched by addition of $NaHCO_3$ (sat. aq. 20 mL). EtOAc (20 mL) was added and the layers were separated. The aqueous

layer was extracted with EtOAc (2 x 20 mL). The combined organic layers were dried (Na_2SO_4), filtered and concentrated in vacuo. The crude product was purified by column chromatography. Compound **6aa** was isolated in 73% yield (0.96 g) as a yellow solid.

6. Control Experiment

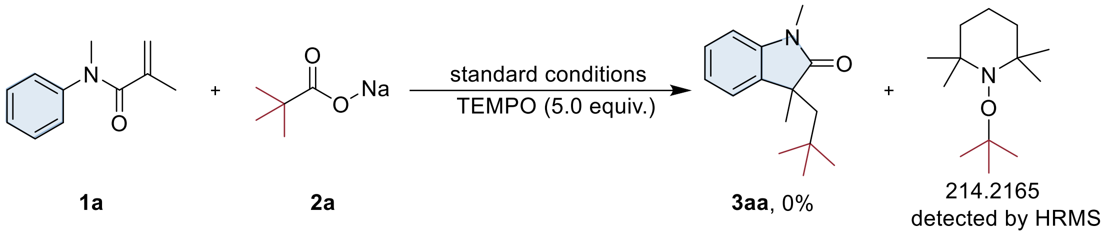

Standard conditions: 1a (0.3 mmol,52.6 mg), **2a** (5 equiv., 0.1862 g), DTBP (3 equiv., 87.13 mg), TEMPO (5 equiv., 0.2344 mg), $FeCl_3$ (0.03 mmol, 4.9 mg) and TBAB (0.015 mmol, 4.8 mg) in MeCN (0.06M, 5 mL) under N_2 atmosphere and stirred under 390 nm light-irradiation (LEDs) at room temperature for 26 h.

HRMS (ESI) m/z: $[M + H]^+$ Calcd. for $C_{13}H_{28}NO$ 214.2165; found 214.2170. As shown in the figure S1 below.

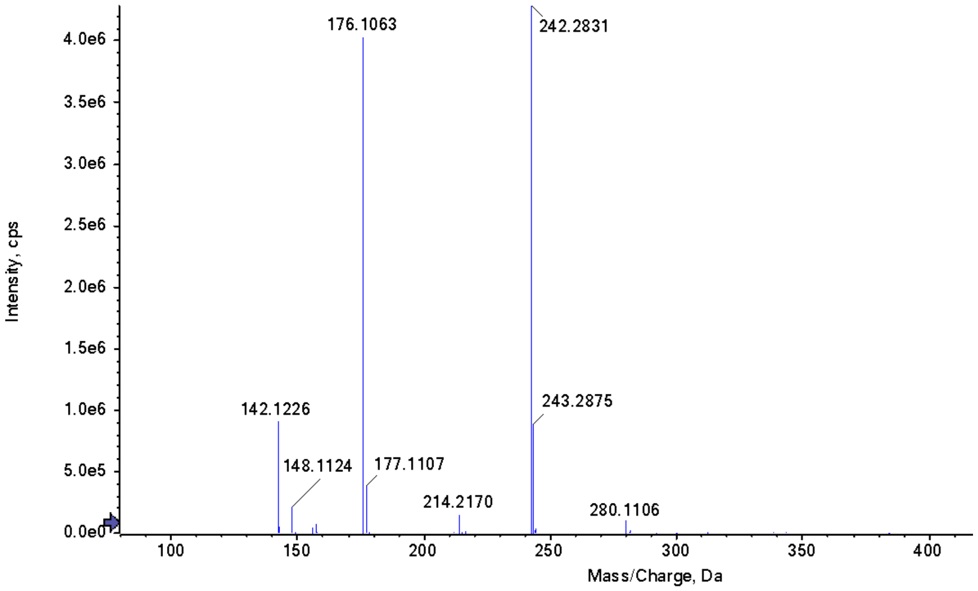

Figure S1: HRMS data of TEMPO adduct

7. "On/off" LED irradiation experiment

An oven dried 10 mL reaction vial was charged with **1a** (0.3 mmol, 1 equiv.), **2a** (1.5 mmol, 5 equiv.), $FeCl_3$ (0.03 mmol, 4.9 mg), TBAB (0.015 mmol, 4.8 mg), DTBP (0.6 mmol, 87.74 mg) and a stir bar. Then MeCN (0.06 M, 5 mL) was added to the glass vial. The reaction vial was sealed, evacuated and backfilled three times with 1 atm of N_2. The mixture was then stirred at 25°C under 390nm blue LED light exposure, following the reaction times specified in Figure S2. After 4 hr of light irradiation, an aliquot portion (10 μL) was taken from the reaction mixture and an analytical sample solution was prepared using biphenyl (1.2 mM) as an internal standard and diluted up to 1 mL with CH_3CN. This resultant solution was analysed in GC to determine the product yield. The results indicated an increase in yield under light exposure, with no significant increase observed after the cessation of light exposure.

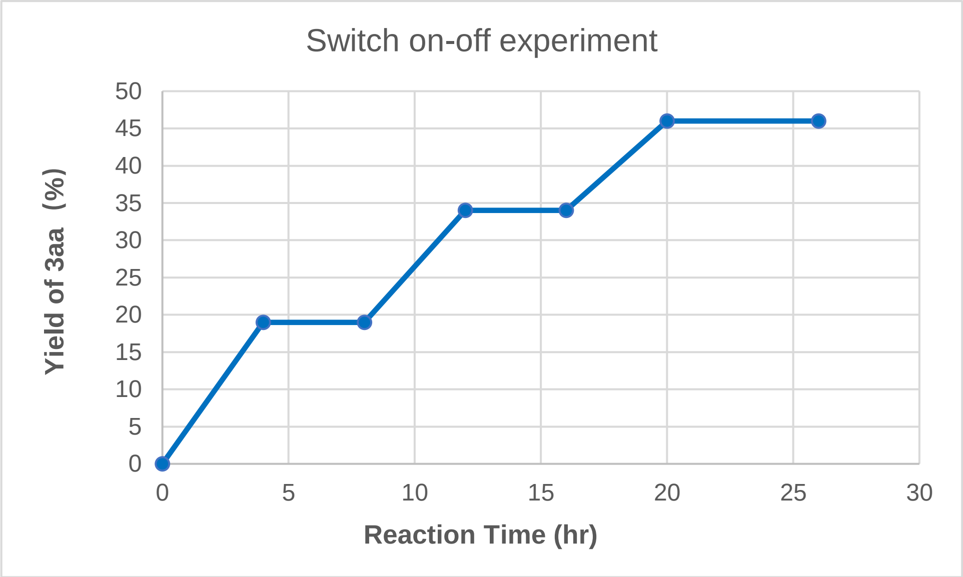

Figure S2: "On/off" LED irradiation experiment for the synthesis of **3aa**

An oven dried 10 mL reaction vial was charged with **4a** (0.3 mmol, 1 equiv.), **5a** (1.5 mmol, 5 equiv.), $FeCl_3$ (0.03 mmol, 4.9 mg), DTBP (1.2 mmol, 0.1743 g) and a stir bar. Then MeCN (0.15 M, 2 mL) and NEt_3 (0.9 mmol, 91.1 mg) were added to the glass vial. The reaction vial was sealed, evacuated and backfilled three times with 1 atm of N_2. The mixture was then stirred at 25°C under 390nm blue LED light exposure, following the reaction times specified in Figure S3. After 6 hr of light irradiation, an aliquot portion (10 µL) was taken from the reaction mixture and an analytical sample solution was prepared using biphenyl (0.9 mM) as an internal standard and diluted up to 1 mL with CH_3CN. This resultant solution was analysed in GC to determine the product yield. The results indicated an increase in yield under light exposure, with no significant increase observed after the cessation of light exposure.

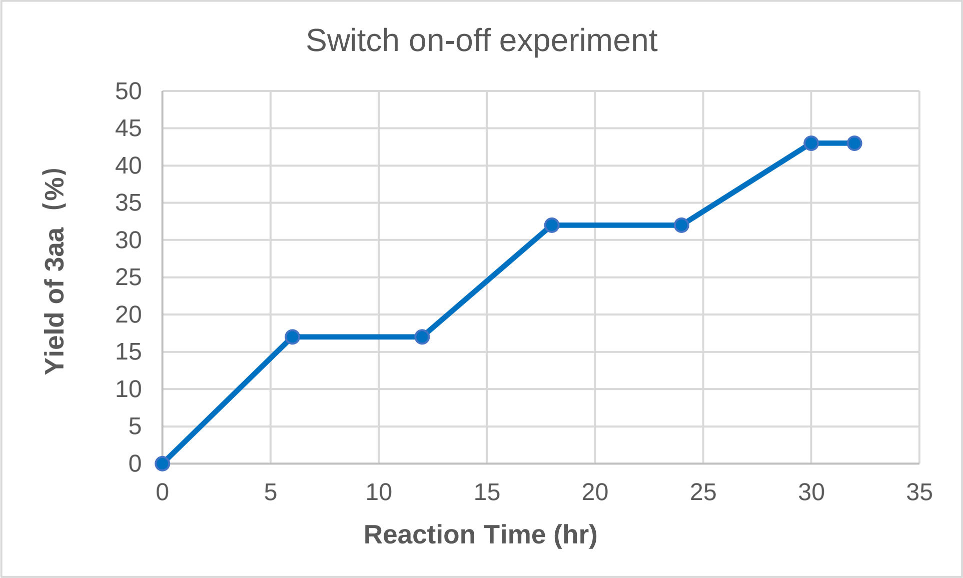

Figure S3: “On/off” LED irradiation experiment for the synthesis of **6aa**

8. UV-vis absorbance experiment

UV-visible absorption Spectra

UV-Vis experiments were performed to analyse the ligand-to-metal-charge-transfer (LMCT) process between Iron salt and alkyl carboxylic acids.

Preparation of a stock solution (solution A): In a glass vial equipped with a teflon-coated stirring bar and a septum, $FeCl_3$ (0.03 mmol) were dissolved in MeCN (25 mL). Dilute 60 μL of the above solution to 3 mL to obtain solution A.

Preparation of a stock solution (solution B): In a glass vial equipped with a teflon-coated stirring bar and a septum, pivalic acid (0.3 mmol) were dissolved in MeCN (25 mL). Dilute 60 μL of the above solution to 3 mL to obtain solution B.

Preparation of a stock solution (solution C): In a glass vial equipped with a teflon-coated stirring bar and a septum, $FeCl_3$ (0.03 mmol) and Pivalic acid (3 mmol) were dissolved in MeCN (25 mL). Dilute 60 μL of the above solution to 3 mL to obtain solution C.

Preparation of a stock solution (solution D): In a glass vial equipped with a teflon-coated stirring bar and a septum, $FeCl_2$ (0.03 mmol) and Pivalic acid (3 mmol) were dissolved in MeCN (25 mL). Dilute 60 μL of the above solution to 3 mL to obtain solution D.

UV-Visible absorption spectra of solution A,solution B and solution C

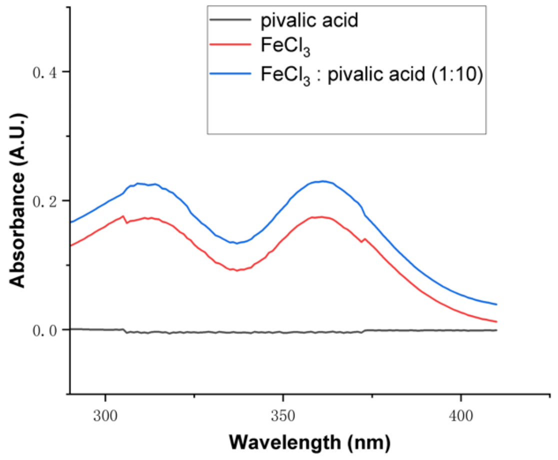

Figure S4: The UV-visible absorption spectra of iron (III) chloride, pivalic acid, and a mixture of iron (III) chloride and pivalic acid solution.

Note: When pivalic acid is added to an iron solution in acetonitrile, the absorption spectrum shows significant enhancement between 300 and 400 nm.

UV-Visible absorption spectra of solution C and solution D

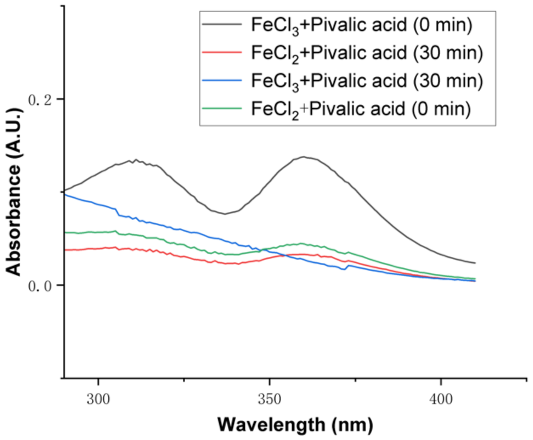

Figure S5: The UV-visible absorption spectra of iron (III) chloride and a mixture of iron (III) chloride and pivalic acid after irradiation with a 390 nm LED.

Note: Under 390 nm irradiation, the UV-visible absorption between 300 and 400 nm decreases, indicating a gradual reduction in the concentration of Fe(III) complexes in the solution.[1]

9. Characterization of products

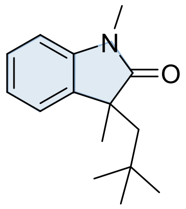

1,3-dimethyl-3-neopentylindolin-2-one (3aa):

The title compound was prepared according to the general procedure-A from **1a** (0.3 mmol, 52.6 mg) and **2a** (1.5 mmol, 0.1862 g). Pure product was obtained as colorless oil in 75% (52.1 mg) yield after flash column chromatography of the crude reaction mixture (silica gel, petroleum ether/ethyl acetate = 20:1-15:1).**^{1}H NMR (400 MHz, DMSO-*d*$_6$)** δ 7.33 (d, *J* = 7.3 Hz, 1H), 7.26 (t, *J* = 7.7 Hz, 1H), 7.06 – 6.98 (m, 2H), 3.13 (s, 3H), 1.93 (s, 2H), 1.18 (s, 3H), 0.54 (s, 9H). **^{13}C{^{1}H} NMR (101 MHz, DMSO-*d*$_6$)** δ 179.9, 142.7, 133.6, 127.6, 123.9, 121.7, 108.4, 50.0, 46.6, 31.5, 30.6, 27.9, 26.0. Data are consistent with reported in the literature.[2]

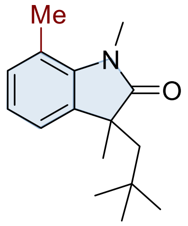

1,3,7-trimethyl-3-neopentylindolin-2-one (3ba):

The title compound was prepared according to the general procedure-A from 1b (0.3 mmol, 56.8 mg) and 2a (1.5 mmol, 0.1862 g). Pure product was obtained as yellow oil in 84% (61.8 mg) yield after flash column chromatography of the crude reaction mixture (silica gel, petroleum ether/ethyl acetate = 20:1-15:1). **^{1}H NMR (400 MHz, CDCl$_3$)** δ 7.02 (d, *J* = 7.1 Hz, 1H), 6.97 (d, *J* = 7.4 Hz, 1H), 6.90 (t, *J* = 7.5 Hz, 1H), 3.50 (s, 3H), 2.59 (s, 3H), 2.13 (d, J = 14.4 Hz, 1H), 1.82 (d, J = 14.4 Hz, 1H), 1.26 (s, 3H), 0.61 (s, 9H). **^{13}C{^{1}H} NMR (101 MHz, CDCl$_3$)** δ 181.8, 140.7, 134.8, 131.2, 121.9, 119.6, 51.0, 46.7, 31.8, 30.9, 29.6, 28.7, 19.1. Data are consistent with reported in the literature.[3]

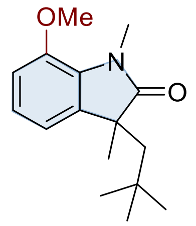

7-methoxy-1,3-dimethyl-3-neopentylindolin-2-one (3ca):

The title compound was prepared according to the general procedure-A from **1c** (0.3 mmol, 61.6 mg) and **2a** (1.5 mmol, 0.1862 g). Pure product was obtained as yellow oil in 75% (58.8 mg) yield after flash column chromatography of the crude reaction mixture (silica gel, petroleum ether/ethyl acetate = 20:1-15:1). **^{1}H NMR (400 MHz, DMSO-*d*$_6$)** δ 6.97 – 6.92 (m, 3H), 3.82 (s, 3H), 3.35 (s, 3H), 1.94 – 1.85 (m, 2H), 1.15 (s, 3H), 0.55 (s, 9H). **^{13}C{^{1}H} NMR (101 MHz, DMSO-*d*$_6$)** δ 180.6, 145.3, 135.6, 130.5, 122.9, 117.3, 112.3, 56.5, 50.6, 47.1, 31.9, 31.0, 29.6, 28.6. **HRMS (ESI/Q-TOF) m/z:** $[M+Na]^+$ calcd. for $C_{16}H_{23}NNaO_2^+$ 284.1621, found 284.1616.

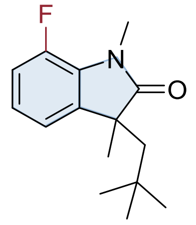

7-fluoro-1,3-dimethyl-3-neopentylindolin-2-one (3da):

The title compound was prepared according to the general procedure-A from **1d** (0.3 mmol, 58.0 mg) and **2a** (1.5 mmol, 0.1862 g). Pure product was obtained as yellow oil in 85% (63.6 mg) yield after flash column chromatography of the crude reaction mixture (silica gel, petroleum ether/ethyl acetate = 20:1-15:1).**^{1}H NMR (400 MHz, $CDCl_3$)** δ 7.00 – 6.89 (m, 3H), 3.43 (s, 3H), 2.15 (d, *J* = 14.4 Hz, 1H), 1.84 (d, *J* = 14.5 Hz, 1H), 1.29 (s, 3H), 0.62 (s, 9H). **^{13}C{^{1}H} NMR (101 MHz, $CDCl_3$)** δ 180.8, 148.0 (d, *J* = 243.3 Hz), 137.4 (d, *J* = 2.9 Hz), 129.6 (d, *J* = 7.9 Hz), 122.6 (d, *J* = 6.3 Hz), 119.9 (d, *J* = 3.2 Hz), 115.5 (d, *J* = 19.3 Hz), 51.1, 47.9 (d, *J* = 1.8 Hz), 31.9, 30.9, 28.8 (d, *J* = 5.8 Hz), 28.62. **^{19}F NMR** (377 MHz, $CDCl_3$) δ -137.19. **HRMS (ESI/Q-TOF) m/z:** $[M+Na]^+$ calcd. for $C_{15}H_{20}FNNaO^+$ 272.1422, found 272.1429.

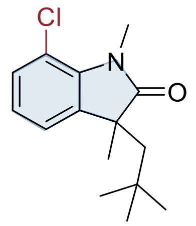

7-chloro-1,3-dimethyl-3-neopentylindolin-2-one (3ea):

The title compound was prepared according to the general procedure-A from **1e** (0.3 mmol, 62.9 mg) and **2a** (1.5 mmol, 0.1862 g). Pure product was obtained as yellow oil in 70% (55.8 mg) yield after flash column chromatography of the crude reaction mixture (silica gel, petroleum ether/ethyl acetate = 20:1-15:1). **^{1}H NMR (400 MHz, DMSO-*d*$_6$)** δ 7.33 (d, *J* = 7.4 Hz, 1H), 7.24 (d, *J* = 8.1 Hz, 1H), 7.05 – 6.97 (m, 1H), 3.46 (s, 3H), 1.94 (s, 2H), 1.19 (s, 3H), 0.54 (s, 9H). **^{13}C{^{1}H} NMR (101 MHz, DMSO-*d*$_6$)** δ 180.2, 138.3, 136.7, 129.6, 123.1, 123.0, 114.1, 50.19, 46.5, 31.4, 30.6, 29.1, 28.0. Data are consistent with reported in the literature.[3]

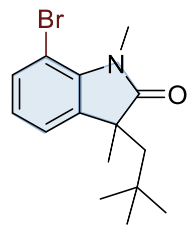

7-bromo-1,3-dimethyl-3-neopentylindolin-2-one (3fa):

The title compound was prepared according to the general procedure-A from **1f** (0.3 mmol, 76.2 mg) and **2a** (1.5 mmol, 0.1862 g). Pure product was obtained as yellow oil in 73% (67.9 mg) yield after flash column chromatography of the crude reaction mixture (silica gel, petroleum ether/ethyl acetate = 20:1-15:1). **^{1}H NMR (400 MHz, DMSO-*d*$_6$)** δ 7.40 (d, *J* = 8.1 Hz, 1H), 7.36 (d, *J* = 7.3 Hz, 1H), 6.94 (t, *J* = 7.7 Hz, 1H), 3.47 (s, 3H), 1.94 (s, 2H), 1.19 (s, 3H), 0.54 (s, 9H). **^{13}C{^{1}H} NMR (101 MHz, DMSO-*d*$_6$)** δ 180.4, 139.7, 137.1, 132.9, 123.5, 123.4, 101.4, 50.2, 46.5, 31.5, 30.6, 29.4, 28.0. Data are consistent with reported in the literature.[4]

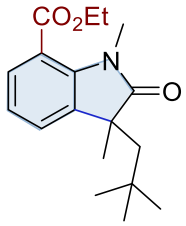

ethyl 1,3-dimethyl-3-neopentyl-2-oxoindoline-7-carboxylate (3ga):

The title compound was prepared according to the general procedure-A from **1g** (0.3 mmol, 74.2 mg) and **2a** (1.5 mmol, 0.1862 g). Pure product was obtained as yellow oil in 85% (77.4 mg) yield after flash column chromatography of the crude reaction mixture (silica gel, petroleum ether/ethyl acetate = 20:1-15:1). **^{1}H NMR (400 MHz, DMSO-*d*$_6$)** δ 7.54 (d, *J* = 7.3 Hz, 1H), 7.48 (d, *J* = 8.0 Hz, 1H), 7.10 (t, *J* = 7.6 Hz, 1H), 4.33 (q, *J* = 7.1 Hz, 2H), 3.15 (s, 3H), 2.01 – 1.93 (m, 2H), 1.33 (s, 3H), 1.21 (s, 3H), 0.54 (s, 9H). **^{13}C{^{1}H} NMR (101 MHz, DMSO-*d*$_6$)** δ 180.9, 166.4, 141.0, 135.5, 128.4, 126.8, 121.3, 115.6, 61.3, 50.2, 45.5, 31.4, 30.6, 29.5, 27.9, 14.0. **HRMS (ESI/Q-TOF) m/z:** [M+Na]$^+$ calcd. for $C_{18}H_{25}NNaO_3^+$ 326.1727, found 326.1725.

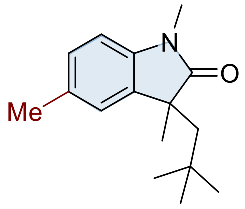

1,3,5-trimethyl-3-neopentylindolin-2-one (3ha):

The title compound was prepared according to the general procedure-A from **1h** (0.3 mmol, 56.8 mg) and **2a** (1.5 mmol, 0.1862 g). Pure product was obtained as White solid in 60% (44.2 mg) yield after flash column chromatography of the crude reaction mixture (silica gel, petroleum ether/ethyl acetate = 20:1-15:1). Mp = 120-121 °C. **^{1}H NMR (400 MHz, DMSO-*d*$_6$)** δ 7.14 (s, 1H), 7.06 (d, *J* = 7.9 Hz, 1H), 6.89 (d, *J* = 7.8 Hz, 1H), 3.10 (s, 3H), 2.27 (s, 3H), 1.90 (d, *J* = 2.8 Hz, 2H), 1.16 (s, 3H), 0.54 (s, 9H). **^{13}C{^{1}H} NMR NMR (101 MHz, DMSO-*d*$_6$)** δ 179.8, 140.3, 133.6, 130.5, 127.7, 124.6, 108.1, 50.0, 46.6, 31.5, 30.6, 27.9, 26.0, 20.8. Data are consistent with reported in the literature.[3]

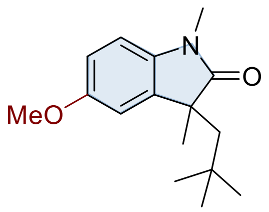

5-methoxy-1,3-dimethyl-3-neopentylindolin-2-one (3ia):

The title compound was prepared according to the general procedure-A from **1i** (0.3 mmol, 61.6 mg) and **2a** (1.5 mmol, 0.1862 g). Pure product was obtained as yellow oil in 58% (45.5 mg) yield after flash column chromatography of the crude reaction mixture (silica gel, petroleum ether/ethyl acetate = 20:1-15:1).**^{1}H NMR (400 MHz, DMSO-*d*$_6$)** δ 7.02 (d, *J* = 2.6 Hz, 1H), 6.92 (d, *J* = 8.4 Hz, 1H), 6.83 – 6.81 (m, 1H), 3.72 (s, 3H), 3.10 (s, 3H), 1.92 (s, 2H), 1.17 (s, 3H), 0.56 (s, 9H). **^{13}C{^{1}H} NMR (101 MHz, DMSO-*d*$_6$)** δ 180.0, 155.6, 136.6, 135.4, 112.4, 111.9, 109.1, 56.0, 50.4, 47.5, 31.9, 31.1, 28.3, 26.5. Data are consistent with reported in the literature.[3]

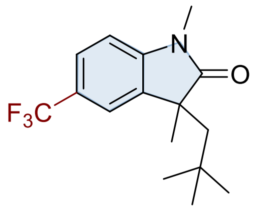

1,3-dimethyl-3-neopentyl-5-(trifluoromethyl)indolin-2-one (3ja):

The title compound was prepared according to the general procedure-A from **1j** (0.3 mmol, 73.0 mg) and **2a** (1.5 mmol, 0.1862 g). Pure product was obtained as yellow oil in 75% (67.4 mg) yield after flash column chromatography of the crude reaction mixture (silica gel, petroleum ether/ethyl acetate = 20:1-15:1).**^{1}H NMR (400 MHz, DMSO-*d*$_6$)** δ 7.76 (s, 1H), 7.64 (d, *J* = 8.2 Hz, 1H), 7.21 (d, *J* = 8.2 Hz, 1H), 3.19 (s, 3H), 2.08 (d, *J* = 14.3 Hz, 1H), 1.93 (d, *J* = 14.3 Hz, 1H), 1.24 (s, 3H), 0.53 (s, 9H). **^{13}C{^{1}H} NMR (101 MHz, DMSO-*d*$_6$)** δ 180.1, 146.1, 134.6, 126.2, 125.3 (q, *J* = 4.0 Hz), 122.4 (q, *J* = 31.7 Hz), 121.1 (q, *J* = 3.7 Hz), 108.6, 49.8, 46.7, 31.4, 30.5, 27.4, 26.2.**^{19}F NMR (377 MHz, DMSO-*d*$_6$)** δ -59.63. Data are consistent with reported in the literature.[3]

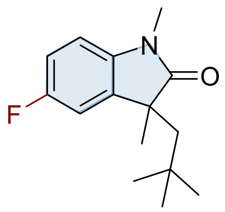

5-fluoro-1,3-dimethyl-3-neopentylindolin-2-one (3ka):

The title compound was prepared according to the general procedure-A from **1k** (0.3 mmol, 58.0 mg) and **2a** (1.5 mmol, 0.1862 g). Pure product was obtained as white solid in 80% (59.8 mg) yield after flash column chromatography of the crude reaction mixture (silica gel, petroleum ether/ethyl acetate = 20:1-15:1). Mp = 121-123 °C. **^{1}H NMR (400 MHz, DMSO-*d*$_6$)** δ 7.31 (dd, *J* = 8.5, 2.6 Hz, 1H), 7.12 – 7.06 (m, 1H), 7.03 – 7.00 (m, 1H), 3.13 (s, 3H), 1.98 – 1.89 (m, 2H), 1.19 (s, 3H), 0.55 (s, 9H). **^{13}C{^{1}H} NMR (101 MHz, DMSO-*d*$_6$)** δ 180.1, 158.8 (d, *J* = 236.9 Hz), 139.3 (d, *J* = 1.7 Hz), 136.1 (d, *J* = 8.3 Hz), 114.1 (d, *J* = 23.5 Hz), 112.5 (d, *J* = 24.7 Hz), 109.6 (d, *J* = 8.2 Hz), 50.3, 47.6 (d, *J* = 1.9 Hz), 31.9, 31.0, 28.1, 26.6. **^{19}F NMR (377 MHz, DMSO-*d*$_6$)** δ -121.78. Data are consistent with reported in the literature.[3]

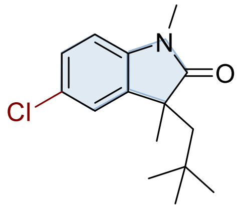

5-chloro-1,3-dimethyl-3-neopentylindolin-2-one (3la)

The title compound was prepared according to the general procedure-A from **1l** (0.3 mmol, 62.9 mg) and **2a** (1.5 mmol, 0.1862 g). Pure product was obtained as white solid in 79% (63.0 mg) yield after flash column chromatography of the crude reaction mixture (silica gel, petroleum ether/ethyl acetate = 20:1-15:1). Mp = 132-134 °C. **^{1}H NMR (400 MHz, DMSO-*d*$_6$)** δ 7.49 (s, 1H), 7.32 (d, *J* = 6.1 Hz, 1H), 7.05 (d, *J* = 8.3 Hz, 1H), 3.14 (s, 3H), 2.01 – 1.88 (m, 2H), 1.20 (s, 3H), 0.56 (s, 9H). **^{13}C{^{1}H} NMR (101 MHz, DMSO-*d*$_6$)** δ 179.5, 141.6, 135.8, 127.4, 126.1, 124.3, 109.8, 49.8, 47.0, 31.5, 30.6, 27.5, 26.2. Data are consistent with reported in the literature.[3]

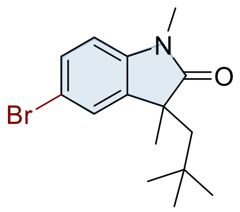

5-bromo-1,3-dimethyl-3-neopentylindolin-2-one (3ma)：

The title compound was prepared according to the general procedure-A from **1l** (0.3 mmol, 76.2 mg) and **2a** (1.5 mmol, 0.1862 g). Pure product was obtained as white solid in 82% (76.3 mg) yield after flash column chromatography of the crude reaction mixture (silica gel, petroleum ether/ethyl acetate = 20:1-15:1). Mp = 147-149 °C. **^{1}H NMR (400 MHz, DMSO-*d*6)** δ 7.59 (s, 1H), 7.44 (d, *J* = 8.3 Hz, 1H), 6.99 (d, *J* = 8.3 Hz, 1H), 3.12 (s, 3H), 1.97 (d, *J* = 6.1 Hz, 1H), 1.90 (d, *J* = 14.3 Hz, 1H), 1.19 (s, 3H), 0.55 (s, 9H). **^{13}C{^{1}H} NMR (101 MHz, DMSO-*d*6)** δ 179.4, 142.0, 136.2, 130.2, 127.0, 113.8, 110.4, 49.8, 47.0, 31.5, 30.6, 27.5, 26.1. Data are consistent with reported in the literature.[3]

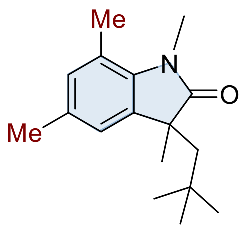

1,3,5,7-tetramethyl-3-neopentylindolin-2-one (3oa)

The title compound was prepared according to the general procedure-A from **1o** (0.3 mmol,61.0 mg) and **2a** (1.5 mmol, 0.1862 g). Pure product was obtained as yellow solid in 86% (66.9 mg) yield after flash column chromatography of the crude reaction mixture (silica gel, petroleum ether/ethyl acetate = 20:1-15:1). Mp = 73-75 °C. **^{1}H NMR (400 MHz, DMSO-*d*6)** δ 6.95 (s, 1H), 6.79 (s, 1H), 3.37 (s, 3H), 2.50 (s, 3H), 2.21 (s, 3H), 1.92 – 1.83 (m, 2H), 1.13 (s, 3H), 0.55 (s, 9H). **^{13}C{^{1}H} NMR (101 MHz, DMSO-*d*6)** δ 180.5, 138.0, 134.3, 131.4, 130.3, 122.4, 119.1, 50.3, 46.0, 31.5, 30.7, 28.9, 28.3, 20.5, 18.4. Data are consistent with reported in the literature.[5]

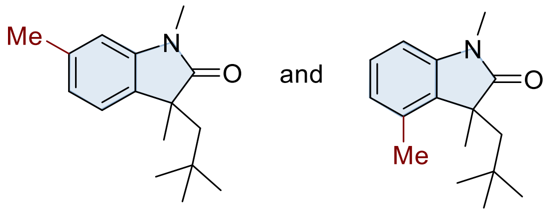

1,3,6-trimethyl-3-neopentylindolin-2-one and 1,3,4-trimethyl-3-neopentylindolin-2-one (3pa+3pa')：

The title compound was prepared according to the general procedure-A from **1o** (0.3 mmol,56.8 mg) and **2a** (1.5 mmol, 0.1862 g). Pure product was obtained as yellow oil in 85% (62.6 mg) yield after flash column chromatography of the crude reaction mixture (silica gel, petroleum ether/ethyl acetate = 20:1-15:1). **^{1}H NMR (400 MHz, DMSO-d_6)** δ 7.17 (t, *J* = 7.7 Hz, 1H), 6.82 (dd, *J* = 23.3, 7.5 Hz, 2H), 3.11 (s, 3H), 2.35 (s, 2H), 2.33 (s, 1H), 2.07 (d, *J* = 14.4 Hz, 0.76H), 1.92 – 1.86 (m, 1.24H), 1.23 (s, 2.14H), 1.15 (s, 0.86H), 0.56 (s, 6.16H), 0.54 (s, 2.77H). **^{13}C{^{1}H} NMR (101 MHz, DMSO-d_6)** δ 180.6, 180.3, 143.4, 143.2, 137.5, 134.8, 131.0, 130.8, 128.1, 125.0, 124.1, 122.6, 109.6, 106.7, 50.4, 48.9, 47.8, 46.8, 31.9, 31.1, 30.2, 28.4, 26.4, 25.5, 21.8, 18.8. Data are consistent with reported in the literature.[3]

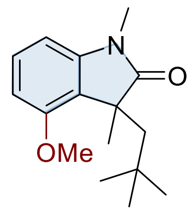

4-methoxy-1,3-dimethyl-3-neopentylindolin-2-one (3qa):

The title compound was prepared according to the general procedure-A from **1o** (0.3 mmol,61.6 mg) and **2a** (1.5 mmol, 0.1862 g). Pure product was obtained as yellow oil in 49% (38.4 mg) yield after flash column chromatography of the crude reaction mixture (silica gel, petroleum ether/ethyl acetate = 20:1-15:1).
^{1}H NMR (400 MHz, DMSO-*d*$_6$) δ 7.25 (t, *J* = 8.0 Hz, 1H), 6.69 – 6.66 (m, 2H), 3.80 (s, 3H), 3.10 (s, 3H), 2.09 (d, *J* = 13.8 Hz, 1H), 1.81 (d, *J* = 13.9 Hz, 1H), 1.20 (s, 3H), 0.55 (s, 9H). **^{13}C{^{1}H} NMR (101 MHz, DMSO-*d*$_6$)** δ 180.1, 156.1, 143.6, 129.1, 118.7, 105.7, 101.9, 55.3, 48.4, 46.6, 31.3, 29.9, 26.2, 25.1. **HRMS (ESI/Q-TOF) m/z:** [M+Na]$^+$ calcd. for $C_{16}H_{23}NNaO_2^+$ 284.1621, found 284.1625.

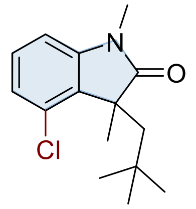

4-chloro-1,3-dimethyl-3-neopentylindolin-2-one (3ra):

The title compound was prepared according to the general procedure-A from **1r** (0.3 mmol,62.9 mg) and **2a** (1.5 mmol, 0.1862 g). Pure product was obtained as yellow oil in 75% (59.8 mg) yield after flash column chromatography of the crude reaction mixture (silica gel, petroleum ether/ethyl acetate = 20:1-15:1).
^{1}H NMR (400 MHz, DMSO-*d*$_6$) δ 7.32 (t, *J* = 8.0 Hz, 1H), 7.06 – 7.01 (m, 2H), 3.15 (s, 3H), 2.26 (d, *J* = 14.3 Hz, 1H), 1.86 (d, *J* = 14.2 Hz, 1H), 1.31 (s, 3H), 0.59 (s, 9H). **^{13}C{^{1}H} NMR NMR (101 MHz, DMSO-*d*$_6$)** δ 179.5, 145.2, 130.7, 130.1, 129.9, 123.4, 108.1, 48.4, 48.2, 31.8, 30.2, 26.8, 24.6. Data are consistent with reported in the literature.[6]

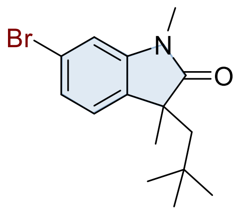

6-bromo-1,3-dimethyl-3-neopentylindolin-2-one (3sa):

The title compound was prepared according to the general procedure-A from **1s** (0.3 mmol,76.2 mg) and **2a** (1.5 mmol, 0.1862 g). Pure product was obtained as yellow oil in 77% (71.7 mg) yield after flash column chromatography of the crude reaction mixture (silica gel, petroleum ether/ethyl acetate = 20:1-15:1). Mp = 135-136 °C. **^{1}H NMR (400 MHz, CDCl$_3$)** δ 7.17 – 7.10 (m, 2H), 6.79 (s, 1H), 3.21 (s, 3H), 2.46 (d, *J* = 14.4 Hz, 1H), 1.94 (d, *J* = 14.4 Hz, 1H), 1.45 (s, 3H), 0.66 (s, 9H). **^{13}C{^{1}H} NMR (101 MHz, CDCl$_3$)** δ 180.4, 145.0, 132.2, 129.1, 126.7, 120.1, 107.1, 49.5, 47.7, 31.7, 30.1, 26.4, 24.2. **HRMS (ESI/Q-TOF) m/z:** [M+Na]$^+$ calcd. for $C_{15}H_{20}BrNNaO^+$ 332.0620, found 332.0627.

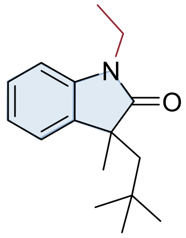

1-ethyl-3-methyl-3-neopentylindolin-2-one (3ta):

The title compound was prepared according to the general procedure-A from **1s** (0.3 mmol,56.8 mg) and **2a** (1.5 mmol, 0.1862 g). Pure product was obtained as yellow solid in 74% (54.5 mg) yield after flash column chromatography of the crude reaction mixture (silica gel, petroleum ether/ethyl acetate = 20:1-15:1). Mp = 60-61 °C. **^{1}H NMR (400 MHz, CDCl$_3$)** δ 7.26 – 7.19 (m, 2H), 7.06 – 6.97 (t, *J* = 7.4 Hz 1H), 6.86 (d, *J* = 7.8 Hz, 1H), 3.92– 3.83 (m, 1H), 3.73– 3.64 (m, 1H), 2.16 (d, *J* = 14.4 Hz, 1H), 1.86 (d, *J* = 14.4 Hz, 1H), 1.28 – 1.25 (m, 6H), 0.63 (s, 9H). **^{13}C{^{1}H} NMR (101 MHz, CDCl$_3$)** δ 180.6, 142.0, 134.5, 127.4, 124.1, 121.7, 108.2, 50.6, 47.4, 34.5, 31.9, 30.9, 28.6, 12.3. Data are consistent with reported in the literature.[3]

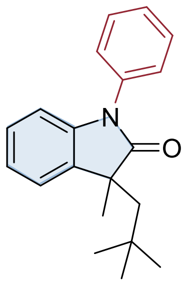

3-methyl-3-neopentyl-1-phenylindolin-2-one (3ua):

The title compound was prepared according to the general procedure-A from **1s** (0.3 mmol,71.2 mg) and **2a** (1.5 mmol, 0.1862 g). Pure product was obtained as white solid in 76% (66.9 mg) yield after flash column chromatography of the crude reaction mixture (silica gel, petroleum ether/ethyl acetate = 20:1-15:1). Mp = 103-104 °C. **^{1}H NMR (400 MHz, CDCl$_3$)** δ 7.55 (t, *J* = 7.7 Hz, 2H), 7.48 – 7.38 (m, 3H), 7.31 (s, 1H), 7.21 (t, *J* = 7.7 Hz, 1H), 7.10 (t, *J* = 7.5 Hz, 1H), 6.89 (d, *J* = 7.9 Hz, 1H), 2.28 (d, *J* = 14.4 Hz, 1H), 1.97 (d, *J* = 14.4 Hz, 1H), 1.45 (s, 3H), 0.76 (s, 9H). **^{13}C{^{1}H} NMR (101 MHz, CDCl$_3$)** δ 180.5, 142.8, 135.0, 134.1, 129.7, 127.9, 127.6, 126.4, 124.4, 122.6, 109.6, 51.1, 47.7, 32.1, 31.2, 29.0. Data are consistent with reported in the literature.[3]

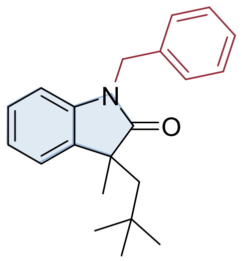

1-benzyl-3-methyl-3-neopentylindolin-2-one (3va)：

The title compound was prepared according to the general procedure-A from **1s** (0.3 mmol,75.4 mg) and **2a** (1.5 mmol, 0.1862 g). Pure product was obtained as white solid in 73% (67.3 mg) yield after flash column chromatography of the crude reaction mixture (silica gel, petroleum ether/ethyl acetate = 20:1-15:1). Mp = 107-108 °C. **^{1}H NMR (400 MHz, $CDCl_3$)** δ 7.36 – 7.22 (m, 6H), 7.17 (t, *J* = 7.7 Hz, 1H), 7.02 (t, *J* = 7.5 Hz, 1H), 6.81 (d, *J* = 7.7 Hz, 1H), 5.09 (d, *J* = 15.5 Hz, 1H), 4.82 (d, *J* = 15.5 Hz, 1H), 2.25 (d, *J* = 14.4 Hz, 1H), 1.94 (d, *J* = 14.4 Hz, 1H), 1.38 (s, 3H), 0.67 (s, 9H). **^{13}C{^{1}H} NMR (101 MHz, $CDCl_3$)** δ 181.1, 142.1, 136.1, 134.2, 128.7, 127.7, 127.6, 127.4, 124.0, 122.0, 109.2, 50.5, 47.5, 43.9, 31.9, 31.0, 29.1. Data are consistent with reported in the literature.[3]

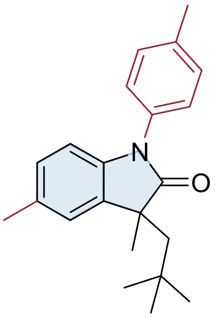

3,5-dimethyl-3-neopentyl-1-(p-tolyl)indolin-2-one (3xa):

The title compound was prepared according to the general procedure-A from **1s** (0.3 mmol,79.6 mg) and **2a** (1.5 mmol, 0.1862 g). Pure product was obtained as yellow solid in 45% (43.4 mg) yield after flash column chromatography of the crude reaction mixture (silica gel, petroleum ether/ethyl acetate = 20:1-15:1). Mp = 104-105 °C. **^{1}H NMR (400 MHz, $CDCl_3$)** δ 7.33 (s, 4H), 7.12 (s, 1H), 7.02 (d, J = 7.9 Hz, 1H), 6.77 (d, J = 7.8 Hz, 1H), 2.45 (s, 3H), 2.39 (s, 3H), 2.27 (d, J = 14.1 Hz, 1H), 1.95 (d, J = 14.4 Hz, 1H), 1.44 (s, 3H), 0.77 (s, 9H). **^{13}C{^{1}H} NMR (101 MHz, $CDCl_3$)** δ 180.4, 140.6, 137.5, 134.1, 132.4, 131.8, 130.1, 127.7, 126.0, 125.0, 109.2, 51.0, 47.6, 32.0, 31.0, 28.9, 21.2, 21.1. **HRMS (ESI/Q-TOF) m/z:** $[M+Na]^+$ calcd. for $C_{22}H_{27}NNaO^+$ 344.1985, found 344.1981.

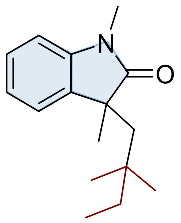

3-(3,3-dimethylbutyl)-1,3-dimethylindolin-2-one (3ab):

The title compound was prepared according to the general procedure-A from **1a** (0.3 mmol,52.6 mg) and **2b** (1.5 mmol, 0.2072 g). Pure product was obtained as colorless oil in 73% (53.7 mg) yield after flash column chromatography of the crude reaction mixture (silica gel, petroleum ether/ethyl acetate = 20:1-15:1). **^{1}H NMR (400 MHz, DMSO-*d*$_6$)** δ 7.33 (d, *J* = 7.5 Hz, 1H), 7.26 (t, *J* = 7.7 Hz, 1H), 7.01 (t, *J* = 6.9 Hz, 2H), 3.13 (s, 3H), 1.91 (s, 2H), 1.18 (s, 3H), 0.94 – 0.83 (m, 2H), 0.67 (t, *J* = 7.4 Hz, 3H), 0.48 (s, 3H), 0.41 (s, 3H). **^{13}C{^{1}H} NMR (101 MHz, DMSO-*d*$_6$)** δ 180.4, 143.0, 134.2, 128.0, 124.2, 122.1, 108.8, 48.0, 46.9, 36.1, 34.1, 28.6, 27.9, 27.2, 26.4, 8.7. Data are consistent with reported in the literature.[7]

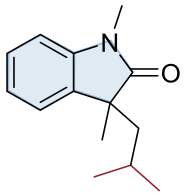

3-isobutyl-1,3-dimethylindolin-2-one (3ac)

The title compound was prepared according to the general procedure-A from **1a** (0.3 mmol,52.6 mg) and **2c** (1.5 mmol, 0.1651 g). Pure product was obtained as yellow oil in 76% (49.6 mg) yield after flash column chromatography of the crude reaction mixture (silica gel, petroleum ether/ethyl acetate = 20:1-15:1). **^{1}H NMR (400 MHz, DMSO-*d*$_6$)** δ 7.30 – 7.24 (m, 2H), 7.08 – 6.98 (m, 2H), 3.12 (s, 3H), 1.82 – 1.70 (m, 2H), 1.20 (s, 3H), 1.16 – 1.07 (m, 1H), 0.55 (dd, *J* = 6.7, 2.1 Hz, 6H). **^{13}C{^{1}H} NMR (101 MHz, DMSO-*d*$_6$)** δ 180.4, 143.4, 133.9, 128.1, 123.3, 122.5, 108.8, 47.7, 46.5, 26.4, 26.3, 25.6, 24.4, 23.1. Data are consistent with reported in the literature.[3]

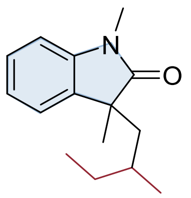

1,3-dimethyl-3-(2-methylbutyl)indolin-2-one (3ad)：

The title compound was prepared according to the general procedure-A from **1a** (0.3 mmol,52.6 mg) and **2d** (1.5 mmol, 0.1862 g). Pure product was obtained as yellow oil in 74% (51.4 mg) yield after flash column chromatography of the crude reaction mixture (silica gel, petroleum ether/ethyl acetate = 20:1-15:1).

^{1}H NMR (400 MHz, DMSO-d_6) δ 7.31 – 7.23 (m, 2H), 7.08 – 6.96 (m, 2H), 3.12 (d, J = 1.3 Hz, 3H), 1.91 – 1.82(m, 1H), 1.69 – 1.63(m, 1H), 1.21 (s, 3H), 1.07 – 1.00 (m, 1H), 0.95 – 0.78 (m, 3H), 0.69 – 0.62 (m, 3H), 0.50 (d, J = 6.6 Hz, 1.5 H), 0.43 (d, J = 6.4 Hz, 1.5 H). **^{13}C{^{1}H} NMR (101 MHz, DMSO-d_6)** δ 180.1&179.7, 143.0&143.0, 133.8&133.3, 127.6, 122.9&122.8, 122.1&122.0, 108.3&108.3, 47.4&47.1, 44.2&43.8, 31.2&31.1, 30.2&29.4, 25.9&25.9, 25.8&25.5, 20.2&19.1, 10.9&10.7. Data are consistent with reported in the literature.[3]

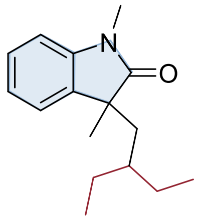

3-(2-ethylbutyl)-1,3-dimethylindolin-2-one (3ae)

The title compound was prepared according to the general procedure-A from **1a** (0.3 mmol,52.6 mg) and **2d** (1.5 mmol, 0.2072 g). Pure product was obtained as colorless oil in 76% (55.9 mg) yield after flash column chromatography of the crude reaction mixture (silica gel, petroleum ether/ethyl acetate = 20:1-15:1). **^{1}H NMR (400 MHz, DMSO-*d*$_6$)** δ 7.31 – 7.23 (m, 2H), 7.09 – 6.97 (m, 2H), 3.13 (s, 3H), 1.75 (d, *J* = 6.1 Hz, 2H), 1.23 (s, 3H), 0.94 – 0.90 (m, 2H), 0.84 (t, *J* = 7.4 Hz, 2H), 0.73 – 0.67 (m, 1H), 0.62 – 0.56(m, 6H). **^{13}C{^{1}H} NMR (101 MHz, DMSO-*d*$_6$)** δ 180.3, 143.5, 134.9, 128.1, 123.3, 122.4, 108.7, 47.7, 41.1, 37.1, 26.3, 26.0, 25.7, 25.4, 10.8, 10.5. Data are consistent with reported in the literature.[7]

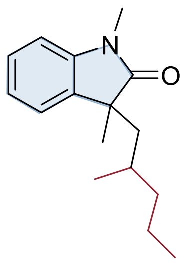

1,3-dimethyl-3-(2-methylpentyl)indolin-2-one (3af)：

The title compound was prepared according to the general procedure-A from **1a** (0.3 mmol,52.6 mg) and **2f** (1.5 mmol, 0.2072 g). Pure product was obtained as yellow oil in 73% (53.7 mg) yield after flash column chromatography of the crude reaction mixture (silica gel, petroleum ether/ethyl acetate = 20:1-15:1).

^{1}H NMR (400 MHz, DMSO-*d*$_6$) δ 7.30 - 7.24 (m, 2H), 7.06 - 7.00 (m, 2H), 3.12 (s, 3H), 1.91 - 1.81 (m, 1H), 1.71 - 1.62 (m, 1H), 1.21 (s, 3H), 1.03 - 0.82 (m, 5H), 0.68 – 0.62 (m, 3H), 0.52 (d, *J* = 6.2 Hz, 1.5H), 0.45 (d, *J* = 6.5 Hz, 1.5H).

^{13}C{^{1}H} NMR (101 MHz, DMSO-d$_6$) δ 180.5&180.2, 143.5&143.4, 134.2&133.8, 128.1, 123.4&123.3, 122.5&122.5, 108.8&108.7, 47.2&47.6, 45.0&44.6, 29.7&29.6, 26.4&26.3, 26.2, 26.0, 21.2, 20.2, 19.5&19.4, 14.0&14.3. Data are consistent with reported in the literature.[3]

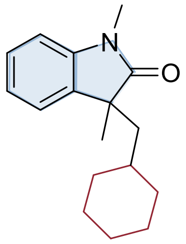

3-(cyclohexylmethyl)-1,3-dimethylindolin-2-one (3ag):

The title compound was prepared according to the general procedure-A from **1a** (0.3 mmol,52.6 mg) and **2g** (1.5 mmol, 0.2237 g). Pure product was obtained as yellow oil in 73% (56.4 mg) yield after flash column chromatography of the crude reaction mixture (silica gel, petroleum ether/ethyl acetate = 20:1-15:1). **^{1}H NMR (400 MHz, DMSO-*d*$_6$)** δ 7.30 – 7.23 (m, 2H), 7.06 – 7.00(m, 2H), 3.13 (s, 3H), 1.77 – 7.15 (m, 2H), 1.50 – 1.37 (m, 3H), 1.27 – 1.15 (m, 5H), 1.00 – 0.66 (m, 6H). **^{13}C{^{1}H} ^{13}C NMR (101 MHz, DMSO-*d*$_6$)** δ 179.9, 142. 9, 133.7, 127.6, 122.7, 122.0, 108.3, 47.0, 44.6, 34.3, 33.8, 32.9, 25.9, 25.8, 25.6, 25.5. Data are consistent with reported in the literature.[3]

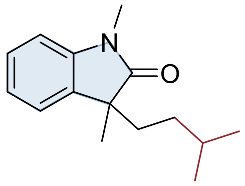

3-isopentyl-1,3-dimethylindolin-2-one (3ah):

The title compound was prepared according to the general procedure-A from **1a** (0.3 mmol, 52.6 mg) and **2h** (1.5 mmol, 0.1862 g). Pure product was obtained as yellow oil in 45% (31.2 mg) yield after flash column chromatography of the crude reaction mixture (silica gel, petroleum ether/ethyl acetate = 20:1-15:1).**^{1}H NMR (400 MHz, DMSO-*d*$_6$)** δ 7.31 – 7.21 (m, 2H), 7.08 – 6.96 (m, 2H), 3.12 (s, 3H), 1.77 – 1.68 (m, 2H), 1.38 – 1.25 (m, 1H), 1.23 (s, 3H), 1.05 (t, J = 7.0 Hz, 1H), 0.72 (t, J = 6.8 Hz, 7H).**^{13}C{^{1}H} NMR (101 MHz, DMSO-*d*$_6$)** δ 180.0, 143.6, 134.0, 128.1, 122.9, 122.6, 108.7, 48.0, 36.0, 33.4, 28.0, 26.3, 24.2, 22.8, 22.7. Data are consistent with reported in the literature.[3]

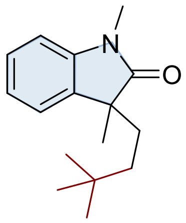

3-(3,3-dimethylbutyl)-1,3-dimethylindolin-2-one (3ai):

The title compound was prepared according to the general procedure-A from **1a** (0.3 mmol, 52.6 mg) and **2i** (1.5 mmol, 0.2072 g). Pure product was obtained as yellow oil in 55% (40.5 mg) yield after flash column chromatography of the crude reaction mixture (silica gel, petroleum ether/ethyl acetate = 20:1-15:1). **^{1}H NMR (400 MHz, DMSO-*d*$_6$)** δ 7.28 – 7.24 (m, 2H), 7.06 – 6.98 (m, 2H), 3.12 (s, 3H), 1.74 – 1.69 (m, 2H), 1.24 (s, 3H), 0.90 – 0.82 (m, 2H), 0.73 (s, 9H). **^{13}C{^{1}H} NMR (101 MHz, DMSO-*d*$_6$)** δ 179.6, 143.2, 133.6, 127.6, 122.4, 122.2, 108.3, 47.5, 37.6, 32.6, 29.6, 29.0, 25.9, 24.0. **HRMS (ESI/Q-TOF) m/z:** $[M+Na]^+$ calcd. for $C_{16}H_{23}NNaO^+$ 268.1672, found 268.1663.

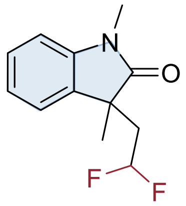

3-(2,2-difluoroethyl)-1,3-dimethylindolin-2-one (3aj)

The title compound was prepared according to the general procedure-A from **1a** (0.3 mmol, 52.6 mg) and **2j** (1.5 mmol, 0.1770 g). Pure product was obtained as yellow oil in 34% (23.0 mg) yield after flash column chromatography of the crude reaction mixture (silica gel, petroleum ether/ethyl acetate = 15:1-10:1). **^{1}H NMR (400 MHz, DMSO-*d*$_6$)** δ 7.45 (d, *J* = 7.3 Hz, 1H), 7.31 (t, *J* = 7.1 Hz, 1H), 7.09 – 7.03 (m, 2H), 5.81 – 5.51 (m, 1H), 3.14 (s, 3H), 2.60 – 2.52 (m, 2H), 1.30 (s, 3H). **^{13}C{^{1}H} NMR (101 MHz, DMSO-*d*$_6$)** δ 178.5, 142.7, 131.7, 128.3, 123.3, 122.3, 115.8 (t, *J* = 238.1 Hz), 108.7, 44.0 (dd, *J* = 7.5, 3.4 Hz), 40.49 – 40.04 (m), 26.13, 24.37. Data are consistent with reported in the literature.[8]

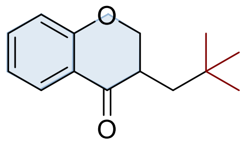

3-neopentylchroman-4-one (6aa):

The title compound was prepared according to the general procedure-B from **4a** (0.3 mmol, 48.7 mg) and **5a** (1.5 mmol, 0.1532 g). Pure product was obtained as yellow solid in 80% (52.4 mg) yield after flash column chromatography of the crude reaction mixture (silica gel, petroleum ether/ethyl acetate = 50:1 - 30:1). Mp = 54 - 56 °C. **^{1}H NMR** (400 MHz, $CDCl_3$) δ 7.89 (dd, *J* = 7.9, 1.8 Hz, 1H), 7.50 – 7.41 (m, 1H), 7.05 – 6.96 (m, 1H), 6.95 (d, *J* = 8.3 Hz, 1H), 4.49 (dd, *J* = 11.3, 5.0 Hz, 1H), 4.18 (t, *J* = 11.1 Hz, 1H), 2.77 – 2.66 (m, 1H), 2.06 (dd, *J* = 14.3, 3.8 Hz, 1H), 1.10 – 1.00 (m, 1H), 0.97 (s, 9H). **^{13}C{^{1}H} NMR (101 MHz, $CDCl_3$)** δ 194.8, 161.5, 135.6, 127. 6, 121.3, 120.8, 117.6, 72.0, 42.8, 38.3, 30.7, 29.5. Data are consistent with reported in the literature.[9]

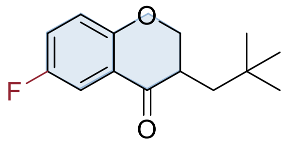

6-fluoro-3-neopentylchroman-4-one (6ba):

The title compound was prepared according to the general procedure-B from **4b** (0.3 mmol, 54.1 mg) and **5a** (1.5 mmol, 0.1532 g). Pure product was obtained as yellow solid in 83% (58.4 mg) yield after flash column chromatography of the crude reaction mixture (silica gel, petroleum ether/ethyl acetate = 50:1 - 30:1). Mp = 76 - 78 °C. **^{1}H NMR (400 MHz, $CDCl_3$)** δ 7.52 (dd, *J* = 8.4, 3.2 Hz, 1H), 7.19 - 7.14 (m, 1H), 6.92 (dd, *J* = 9.0, 4.2 Hz, 1H), 4.49 - 4.45 (m, 1H), 4.15 (t, *J* = 11.1 Hz, 1H), 2.73 - 2.67 (m, 1H), 2.03 (dd, *J* = 14.3, 3.8 Hz, 1H), 1.04 (d, *J* = 8.6 Hz, 1H), 0.96 (s, 9H). **^{13}C{^{1}H} NMR (101 MHz, $CDCl_3$)** δ 194.1 (d, *J* = 1.8 Hz), 157.83 (d, J = 2.0 Hz), 157.3 (d, J = 242.4 Hz), 123.2 (d, *J* = 24.6 Hz), 121.3 (d, *J* = 6.3 Hz), 119.3 (d, *J* = 7.3 Hz), 112.6 (d, *J* = 23.2 Hz), 72.2, 42.8, 38.4, 30.8, 29.6. **^{19}F NMR (377 MHz, $CDCl_3$)** δ -121.75. Data are consistent with reported in the literature.[9]

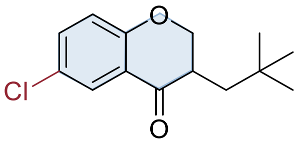

6-chloro-3-neopentylchroman-4-one (6ca):

The title compound was prepared according to the general procedure-B from **4c** (0.3 mmol, 59.0 mg) and **5a** (1.5 mmol, 0.1532 g). Pure product was obtained as yellow solid in 75% (56.9 mg) yield after flash column chromatography of the crude reaction mixture (silica gel, petroleum ether/ethyl acetate = 50:1 - 30:1). Mp = 43-46 °C. **^{1}H NMR** (400 MHz, $CDCl_3$) δ 7.85 – 7.80 (m, 1H), 7.41 – 7.34 (m, 1H), 6.90 (d, *J* = 8.9 Hz, 1H), 4.49 (dd, *J* = 11.4, 5.0 Hz, 1H), 4.16 (t, *J* = 11.1 Hz, 1H), 2.75 – 2.64 (m, 1H), 2.03 (dd, *J* = 14.4, 3.9 Hz, 1H), 1.04 (d, *J* = 8.9 Hz, 1H), 0.96 (s, 9H). **^{13}C{^{1}H} NMR (101 MHz, $CDCl_3$)** δ 193.7, 160.0, 135.5, 127.0, 126.9, 121.7, 119.5, 72.2, 42.7, 38.38, 30.8, 29.6. Data are consistent with reported in the literature.[9]

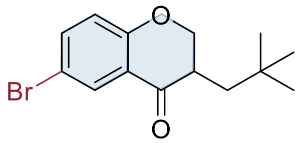

6-bromo-3-neopentylchroman-4-one (6da):

The title compound was prepared according to the general procedure-B from **4d** (0.3 mmol, 72.3 mg) and **5a** (1.5 mmol, 0.1532 g). Pure product was obtained as yellow solid in 79% (70.4 mg) yield after flash column chromatography of the crude reaction mixture (silica gel, petroleum ether/ethyl acetate = 50:1 - 30:1). Mp = 52 - 54 °C.**^{1}H NMR** (400 MHz, $CDCl_3$) δ 7.77 (d, J = 2.5 Hz, 1H), 7.31 (q, *J* = 3.2 Hz, 1H), 6.87 – 6.82 (m, 1H), 4.52 – 4.47 (m, 1H), 4.13 – 4.07 (m, 1H), 2.67 – 2.60 (m, 1H), 1.97 (d, *J* = 14.6 Hz, 1H), 0.98 (dd, *J* = 14.1, 3.5 Hz, 1H), 0.89 (s, 9H). **^{13}C{^{1}H} NMR (101 MHz, $CDCl_3$)** δ 193.8, 160.0, 135.5, 127.0, 126.9, 121.7, 119.5, 72.2, 42.8, 38.4, 30.9, 29.6. Data are consistent with reported in the literature.[9]

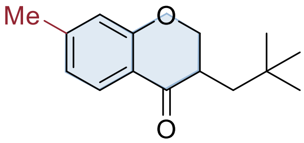

7-methyl-3-neopentylchroman-4-one (6ea):

The title compound was prepared according to the general procedure-B from **4e** (0.3 mmol, 52.9 mg) and **5a** (1.5 mmol, 0.1532 g). Pure product was obtained as yellow oil in 65% (45.3 mg) yield after flash column chromatography of the crude reaction mixture (silica gel, petroleum ether/ethyl acetate = 50:1 - 30:1). **^{1}H NMR** (400 MHz, $CDCl_3$) δ 7.77 (d, *J* = 8.0 Hz, 1H), 6.81 (dd, *J* = 8.0, 1.6 Hz, 1H), 6.74 (s, 1H), 4.46 (dd, *J* = 11.3, 5.0 Hz, 1H), 4.14 (t, *J* = 11.0 Hz, 1H), 2.70 – 2.64 (m, 1H), 2.34 (s, 3H), 2.04 (dd, *J* = 14.3, 3.7 Hz, 1H), 1.06 – 1.01 (m, 1H), 0.96 (s, 9H). **^{13}C{^{1}H} NMR (101 MHz, $CDCl_3$)** δ 194.5, 161.5, 147.0, 127.4, 122.6, 118.6, 117.6, 72.0, 42.8, 38.4, 30.7, 29.5, 21.9. **HRMS (ESI/Q-TOF) m/z:** $[M+Na]^+$ calcd. for $C_{15}H_{20}NaO_2^+$ 255.1356, found 255.1352.

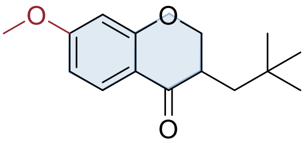

7-methoxy-3-neopentylchroman-4-one (6fa):

The title compound was prepared according to the general procedure-B from **4f** (0.3 mmol, 57.7 mg) and **5a** (1.5 mmol, 0.1532 g). Pure product was obtained as yellow solid in 54% (40.2 mg) yield after flash column chromatography of the crude reaction mixture (silica gel, petroleum ether/ethyl acetate = 50:1 - 30:1). Mp = 66 - 68 °C.^{1}**H NMR** (400 MHz, $CDCl_3$) δ 7.31 (d, *J* = 3.2 Hz, 1H), 7.06 (dd, *J* = 9.0, 3.2 Hz, 1H), 6.87 (d, *J* = 9.0 Hz, 1H), 4.45 (dd, *J* = 11.3, 4.9 Hz, 1H), 4.13 (t, *J* = 11.0 Hz, 1H), 3.79 (s, 3H), 2.71 – 2.65 (m, 1H), 2.03 (dd, *J* = 14.3, 3.8 Hz, 1H), 1.08 – 1.02 (m, 1H), 0.96 (s, 9H). **$^{13}C\{^{1}H\}$ NMR (101 MHz, $CDCl_3$)** δ 194.6, 151.4, 148.6, 121.4, 120.7, 118.7, 116.2, 72.4, 56.2, 42.7, 38.2, 30.7, 29.4. Data are consistent with reported in the literature.[9]

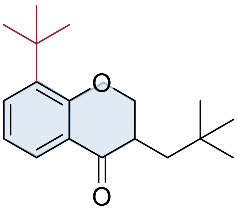

8-(tert-butyl)-3-neopentylchroman-4-one (6ga):

The title compound was prepared according to the general procedure-B from **4g** (0.3 mmol, 65.5 mg) and **5a** (1.5 mmol, 0.1532 g). Pure product was obtained as yellow oil in 65% (53.5 mg) yield after flash column chromatography of the crude reaction mixture (silica gel, petroleum ether/ethyl acetate = 50:1 - 30:1). **^{1}H NMR (400 MHz, $CDCl_3$)** δ 7.80 (d, *J* = 7.8 Hz, 1H), 7.45 (d, *J* = 7.7 Hz, 1H), 6.94 (t, *J* = 7.7 Hz, 1H), 4.55 (dd, *J* = 11.2, 5.2 Hz, 1H), 4.17 (t, *J* = 11.0 Hz, 1H), 2.74 – 2.67 (m, 1H), 2.05 (dd, *J* = 14.3, 4.0 Hz, 1H), 1.40 (s, 9H), 1.09 – 1.04 (m, 1H), 0.99 (s, 9H). **$^{13}C\{^{1}H\}$ NMR (101 MHz, $CDCl_3$)** δ 195.5, 160.7, 138.7, 132.5, 125.7, 121.6, 120.7, 71.4, 42.8, 38.6, 34.9, 30.7, 29.6, 29.6. **HRMS (ESI/Q-TOF) m/z:** $[M+H]^+$ calcd. for $C_{18}H_{27}O_2^+$ 275.2006, found 275.1995.

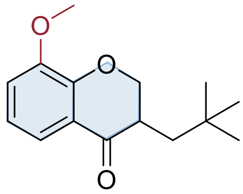

7-methoxy-3-neopentylchroman-4-one (6ha)

The title compound was prepared according to the general procedure-B from **4h** (0.3 mmol, 57.7 mg) and **5a** (1.5 mmol, 0.1532 g). Pure product was obtained as yellow oil in 54% (40.2 mg) yield after flash column chromatography of the crude reaction mixture (silica gel, petroleum ether/ethyl acetate = 50:1 - 30:1). Mp = 66 - 68 °C. **^{1}H NMR (400 MHz, $CDCl_3$)** δ 7.49 (dd, = 8.0, 1.6 Hz, 1H), 7.03 (dd, *J* = 7.9, 1.6 Hz, 1H), 6.95 (t, *J* = 7.9 Hz, 1H), 4.60 (dd, *J* = 11.3, 4.9 Hz, 1H), 4.25 (t, *J* = 11.0 Hz, 1H), 3.91 (s, 3H), 2.76 – 2.69 (m, 1H), 2.04 (dd, *J* = 14.3, 3.7 Hz, 1H), 1.09 (dd, *J* = 14.3, 5.8 Hz, 1H), 0.96 (s, 9H). **^{13}C NMR (101 MHz, $CDCl_3$)** δ 194.9, 156.1, 154.0, 124.7, 120.6, 118.9, 108.0, 72.1, 55.8, 42.8, 38.4, 30.7, 29.5. HRMS (ESI/Q-TOF) m/z: $[M+Na]^+$ calcd. for $C_{15}H_{20}NaO_3^+$ 271.1305, found 271.1296.

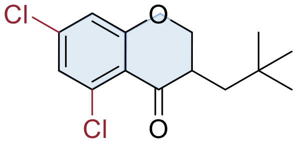

5,7-dichloro-3-neopentylchroman-4-one (6ia):

The title compound was prepared according to the general procedure-B from **4i** (0.3 mmol, 69.3 mg) and **5a** (1.5 mmol, 0.1532 g). Pure product was obtained as yellow solid in 70% (60.3 mg) yield after flash column chromatography of the crude reaction mixture (silica gel, petroleum ether/ethyl acetate = 50:1 - 30:1). Mp = 60 - 62 °C. **^{1}H NMR** (400 MHz, $CDCl_3$) δ 7.77 (s, 1H), 7.53 (s, 1H), 4.64 (dd, *J* = 11.4, 5.0 Hz, 1H), 4.25 (t, *J* = 11.4 Hz, 1H), 2.78 – 2.72(m, 1H), 2.06 – 2.01 (m, 1H), 1.07 – 1.03 (m, 1H), 0.97 (s, 9H). **^{13}C{^{1}H} NMR (101 MHz, $CDCl_3$)** δ 192.7, 155.6, 135.1, 126.5, 125.7, 123.5, 122.4, 72.6, 42.4, 38.1, 30.7, 29.4. Data are consistent with reported in the literature.[9]

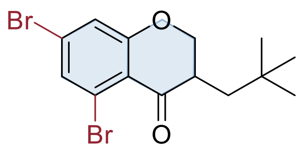

5,7-dibromo-3-neopentylchroman-4-one (6ja):

The title compound was prepared according to the general procedure-B from **4j** (0.3 mmol, 96.0 mg) and **5a** (1.5 mmol, 0.1532 g). Pure product was obtained as yellow oil in 82% (92.5 mg) yield after flash column chromatography of the crude reaction mixture (silica gel, petroleum ether/ethyl acetate = 50:1 - 30:1). **^{1}H NMR** (400 MHz, $CDCl_3$) δ 7.96 (s, 1H), 7.82 (s, 1H), 4.64 (dd, *J* = 11.5, 5.1 Hz, 1H), 4.25 (t, *J* = 11.3 Hz, 1H), 2.79 – 2.69 (m, 1H), 2.05 (dd, *J* = 14.4, 2.3 Hz, 1H), 1.05 (dd, *J* = 8.6, 1.6 Hz, 1H), 0.97 (s, 9H). **^{13}C{^{1}H} NMR (101 MHz, $CDCl_3$)** δ 192.6, 156.9, 140.7, 129.5, 122.7, 113.8, 112.4, 72.6, 42.2, 38.1, 30.7, 29.4, 29.4. **HRMS (ESI/Q-TOF) m/z:** $[M+H]^+$ calcd. for $C_{14}H_{17}Br_2O_2^+$ 376.3175, found 376.3180.

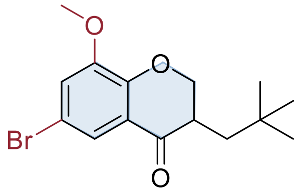

7-bromo-5-methoxy-3-neopentylchroman-4-one (6ka)

The title compound was prepared according to the general procedure-B from 4k (0.3 mmol, 81.3 mg) and 5a (1.5 mmol, 0.1532 g). Pure product was obtained as white solid in 73% (71.7 mg) yield after flash column chromatography of the crude reaction mixture (silica gel, petroleum ether/ethyl acetate = 50:1 - 30:1). Mp = 89-90 °C. **^{1}H NMR (400 MHz, $CDCl_3$)** δ 7.60 (s, 1H), 7.10 (s, 1H), 4.59 (dd, J = 11.4, 4.8 Hz, 1H), 4.24 (t, *J* = 11.3 Hz, 1H), 3.90 (s, 3H), 2.71 (dd, *J* = 10.6, 5.2 Hz, 1H), 2.01 (dd, *J* = 14.1, 3.1 Hz, 1H), 1.08 (d, *J* = 8.9 Hz, 1H), 0.95 (s, 9H). **^{13}C{^{1}H} NMR (101 MHz, $CDCl_3$)** δ 193.4, 150.7, 149.7, 122.2, 121.2, 119.4, 113.4, 72.6, 56.6, 42.7, 38.3, 30.9, 29.5. **HRMS (ESI/Q-TOF) m/z:** $[M+Na]^+$ calcd. for $C_{15}H_{19}BrNaO_3^+$ 349.0410, found 349.0393.

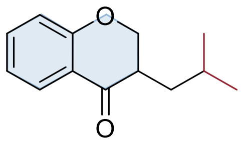

3-isobutylchroman-4-one (6ab)

The title compound was prepared according to the general procedure-B from 4a (0.3 mmol, 48.7 mg) and 5b (1.5 mmol, 0.1322 g). Pure product was obtained as yellow oil in 75% (46.0 mg) yield after flash column chromatography of the crude reaction mixture (silica gel, petroleum ether/ethyl acetate = 100:1 - 80:1). ^{1}H NMR (400 MHz, $CDCl_3$) δ 7.89 (dd, J = 7.8, 1.8 Hz, 1H), 7.50 – 7.41 (m, 1H), 7.03 – 6.99 (m, 1H), 6.97 – 6.94 (m, 1H), 4.50 (dd, J = 11.4, 4.4 Hz, 1H), 4.26 – 4.21 (m, 1H), 2.76 – 2.70 (m, 1H), 1.77 – 1.75 (m, 1H), 1.29 – 1.22 (m, 2H), 0.98 (d, J = 6.4 Hz, 3H), 0.94 (d, J = 6.4 Hz, 3H). ^{13}C{^{1}H} NMR (101 MHz, $CDCl_3$) δ 194.8, 161.4, 135.7, 127.4, 121.3, 120.6, 117.6, 70.6, 44.0, 35.1, 25.6, 23.0, 22.0. **HRMS (ESI/Q-TOF) m/z:** $[M+Na]^+$ calcd. for $C_{13}H_{16}NaO_2^+$ 227.1043, found 227.1040.

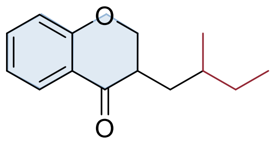

3-(2-methylbutyl)chroman-4-one (6ac):

The title compound was prepared according to the general procedure-B from **4a** (0.3 mmol, 48.7 mg) and **5c** (1.5 mmol, 0.1532 g). Pure product was obtained as yellow oil in 70% (45.8 mg) yield after flash column chromatography of the crude reaction mixture (silica gel, petroleum ether/ethyl acetate = 100:1 - 80:1). **^{1}H NMR (400 MHz, $CDCl_3$)** δ 7.88 (d, *J* = 10.5 Hz, 1H), 7.45 (t, *J* = 7.8 Hz, 1H), 7.00 (t, *J* = 7.5 Hz, 1H), 6.94 (d, *J* = 8.4 Hz, 1H), 4.52 – 4.47 (m, 1H), 4.27 – 4.19 (m, 1H), 2.79 – 2.68 (m, 1H), 1.46 – 1.34 (m, 3H), 1.27 – 1.12 (m, 3H), 0.95 – 0.87 (m, 6H).**^{13}C{^{1}H} NMR (101 MHz, $CDCl_3$)** δ 195.1,&195.0, 161.5&161.5, 135.8&135.7, 127.5,&127.5, 121.4&121.4, 120.8&120.7, 117.7&117.7, 71.1&70.4, 43.9&43.8, 33.5&32.8, 32.0&31.9, 30.0&29.0, 19.5&18.7, 11.4&11.1 **HRMS (ESI/Q-TOF) m/z:** $[M+Na]^+$ calcd. for $C_{14}H_{18}NaO_2^+$ 241.1199, found 241.1185.

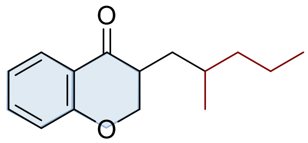

3-(2-methylpentyl)chroman-4-one (6ad):

The title compound was prepared according to the general procedure-B from **4a** (0.3 mmol, 48.7 mg) and **5d** (1.5 mmol, 0.1742 g). Pure product was obtained as yellow oil in 50% (34.9 mg) yield after flash column chromatography of the crude reaction mixture (silica gel, petroleum ether/ethyl acetate = 100:1 - 80:1). **^{1}H NMR (400 MHz, $CDCl_3$)** δ 7.88 (d, J = 7.8 Hz, 1H), 7.45 (t, J = 7.5 Hz, 1H), 7.00 (t, J = 7.5 Hz, 1H), 6.95 (d, J = 8.4 Hz, 1H), 4.54 – 4.45 (m, 1H), 4.28 – 4.17 (m, 1H), 2.80 – 2.68 (m, 1H), 1.78 – 1.65 (m, 1H), 1.38 – 1.20 (m, 6H), 0.94 – 0.86 (m, 6H). **^{13}C{^{1}H} NMR (101 MHz, $CDCl_3$)** δ 195.0&194.9, 161.5&161.4, 135.7&135.7, 127.5&127.4, 121.3, 120.7&120.6, 117.7&117.6, 71.0&70.3, 43.8&43,7, 39.7&38.7, 33.7&33.1, 30.2&30.0, 20.1&19.1, 19.9&19.8, 14,3&14.3. **HRMS (ESI/Q-TOF) m/z:** $[M+Na]^+$ calcd. for $C_{15}H_{20}NaO_2^+$ 255.1356, found 255.1349.

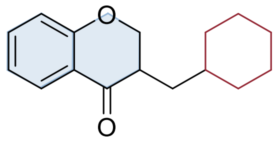

3-(cyclohexylmethyl)chroman-4-one (6ae):

The title compound was prepared according to the general procedure-B from **4a** (0.3 mmol, 48.7 mg) and **5e** (1.5 mmol, 0.1923 g). Pure product was obtained as yellow oil in 53% (38.9 mg) yield after flash column chromatography of the crude reaction mixture (silica gel, petroleum ether/ethyl acetate = 100:1 - 80:1). **^{1}H NMR (400 MHz, $CDCl_3$)** δ 7.88 (d, J = 7.7 Hz, 1H), 7.44 (t, J = 7.7 Hz, 1H), 6.99 (t, J = 7.6 Hz, 1H), 6.94 (d, J = 8.4 Hz, 1H), 4.51 – 4.46 (m, 1H), 4.25 – 4.19 (m, 1H), 2.80 – 2.73 (m, 1H), 1.77 – 1.71 (m, 4H), 1.33 – 1.17 (m, 6H), 1.00 – 0.87 (m, 3H). **^{13}C{^{1}H} NMR (101 MHz, $CDCl_3$)** δ 195.0, 161.4, 135.6, 127.4, 121.3, 120.6, 117.6, 70.7, 43.2, 35.0, 33.8, 33.6, 32.8, 26.5, 26.2, 26.1. **HRMS (ESI/Q-TOF) m/z:** $[M+H]^+$ calcd. for $C_{15}H_{21}O_2^+$ 245.1537, found 245.1530.

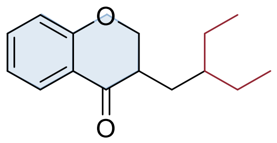

3-(2-ethylbutyl)chroman-4-one (6af):

The title compound was prepared according to the general procedure-B from **4a** (0.3 mmol, 48.7 mg) and **5e** (1.5 mmol, 0.1742 g). Pure product was obtained as yellow oil in 62% (43.2 mg) yield after flash column chromatography of the crude reaction mixture (silica gel, petroleum ether/ethyl acetate = 100:1 - 80:1). **^{1}H NMR (400 MHz, $CDCl_3$)** δ 7.92 (dd, J = 7.9, 1.8 Hz, 1H), 7.49 (s, 1H), 7.04 (t, *J* = 7.5 Hz, 1H), 6.99 (d, *J* = 8.3 Hz, 1H), 4.54 (dd, *J* = 11.4, 4.4 Hz, 1H), 4.27 (dd, *J* = 11.4, 8.5 Hz, 1H), 2.75 - 2.68 (m, 1H), 1.87 (dd, *J* = 12.6, 5.7 Hz, 1H), 1.42 – 1.35 (m, 6H), 0.95 – 0.86 (m, 6H). **^{13}C{^{1}H} NMR (101 MHz, $CDCl_3$)** δ 195.0, 161.4, 135.6, 127.4, 121.3, 120.7, 117.6, 70.7, 43.8, 37.7, 29.6, 25.5, 24.9, 10.8, 10.3. **HRMS (ESI/Q-TOF) m/z:** $[M+Na]^+$ calcd. for $C_{15}H_{20}NaO_2^+$ 255.1356, found 255.1346.

10. References

1. Jin, Y.; Zhang, Q.; Wang, L.; Wang, X.; Meng, C.; Duan, C., Convenient C(sp^3)–H bond functionalisation of light alkanes and other compounds by iron photocatalysis. *Green Chemistry* **2021,** *23*, 6984-6989.
2. Muralirajan, K.; Kancherla, R.; Gimnkhan, A.; Rueping, M., Unactivated Alkyl Chloride Reactivity in Excited-State Palladium Catalysis. *Organic Letters* **2021,** *23*, 6905-6910.
3. Yang, L.; Lu, W.; Zhou, W.; Zhang, F., Metal-free cascade oxidative decarbonylative alkylarylation of acrylamides with aliphatic aldehydes: a convenient approach to oxindoles via dual C(sp^2)–H bond functionalization. *Green Chemistry* **2016,** *18*, 2941-2945.
4. Zhao, Y.; Li, Z.; Sharma, U. K.; Sharma, N.; Song, G.; Van der Eycken, E. V., Copper-catalyzed alkylarylation of activated alkenes using isocyanides as the alkyl source: an efficient radical access to 3,3-dialkylated oxindoles. *Chemical Communications* **2016,** *52*, 6395-6398.
5. Wu, T.; Zhang, H.; Liu, G., Organocatalyzed arylalkylation of activated alkenes via decarboxylation of $PhI(O_2CR)_2$: efficient synthesis of oxindoles. *Tetrahedron* **2012,** *68*, 5229-5233.
6. Tang, Q.; Liu, X.; Liu, S.; Xie, H.; Liu, W.; Zeng, J.; Cheng, P., *N*-(Acyloxy)phthalimides as tertiary alkyl radical precursors in the visible light photocatalyzed tandem radical cyclization of N-arylacrylamides to 3,3-dialkyl substituted oxindoles. *RSC Advances* **2015,** *5*, 89009-89014.
7. Xie, J.; Xu, P.; Li, H.; Xue, Q.; Jin, H.; Cheng, Y.; Zhu, C., A room temperature decarboxylation/C–H functionalization cascade by visible-light photoredox catalysis. *Chemical Communications* **2013,** *49*, 5672-5674.
8. Gui, Q.-W.; Teng, F.; Li, Z.-C.; Xiong, Z.-Y.; Jin, X.-F.; Lin, Y.-W.; Cao, Z.; He, W.-M., Visible-light-initiated tandem synthesis of difluoromethylated oxindoles in 2-MeTHF under additive-, metal catalyst, external photosensitizer-free and mild conditions. *Chinese Chemical Letters* **2021,** *32*, 1907-1910.
9. Hu, H.; Chen, X.; Sun, K.; Wang, J.; Liu, Y.; Liu, H.; Yu, B.; Sun, Y.; Qu, L.; Zhao, Y., Silver-catalyzed decarboxylative cascade radical cyclization of tert-carboxylic acids and *o*-(allyloxy)arylaldehydes towards chroman-4-one derivatives. *Organic Chemistry Frontiers* **2018,** *5*, 2925-2929.

11. NMR Spectra

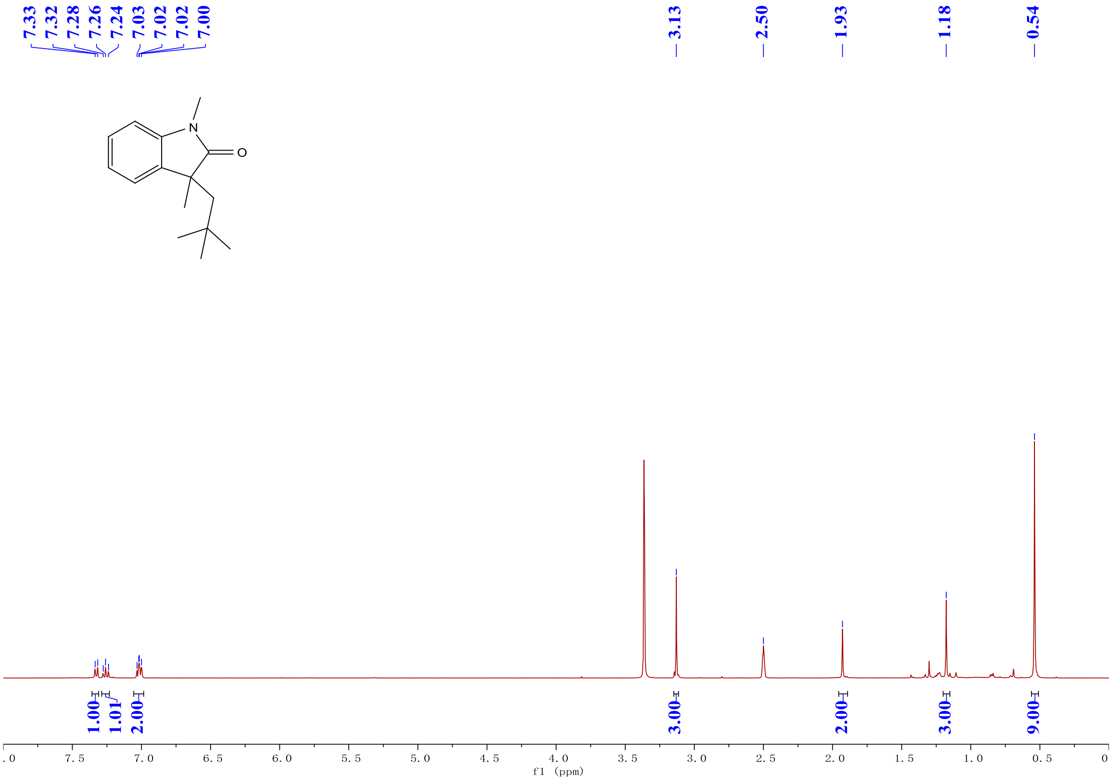

^{1}H NMR spectrum of **3aa** (400 MHz, DMSO-d_6)

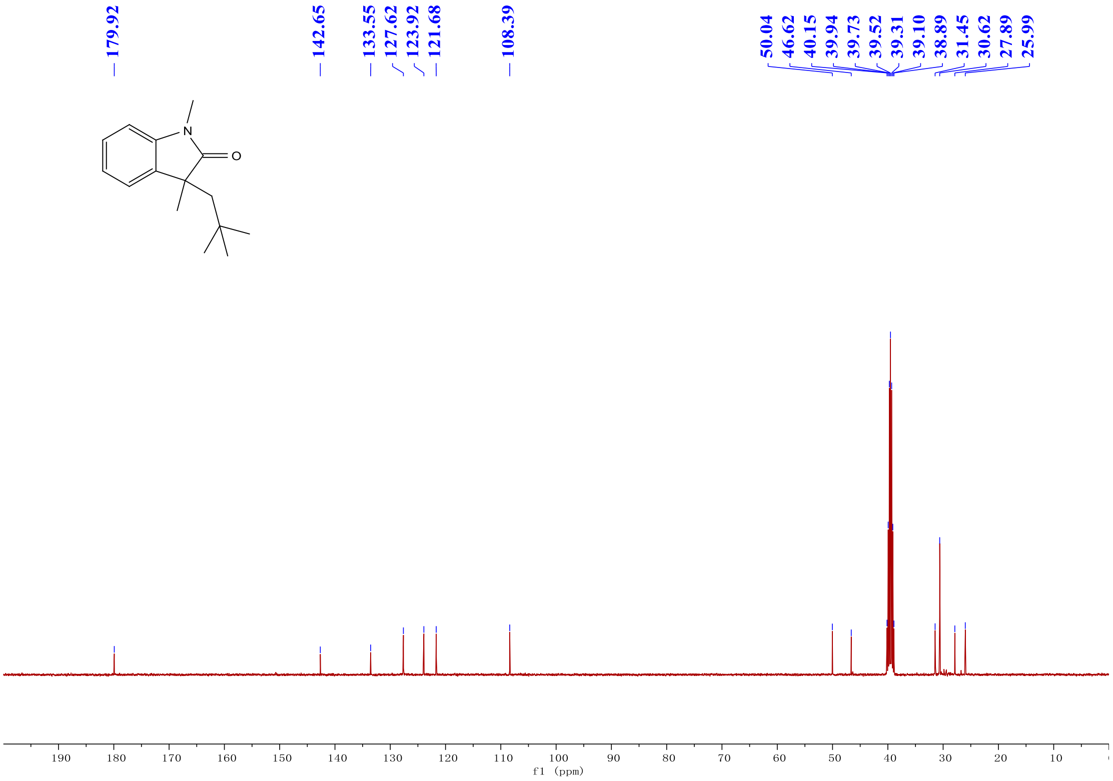

^{13}C {^{1}H} NMR spectrum of **3aa** (101 MHz, DMSO-d_6)

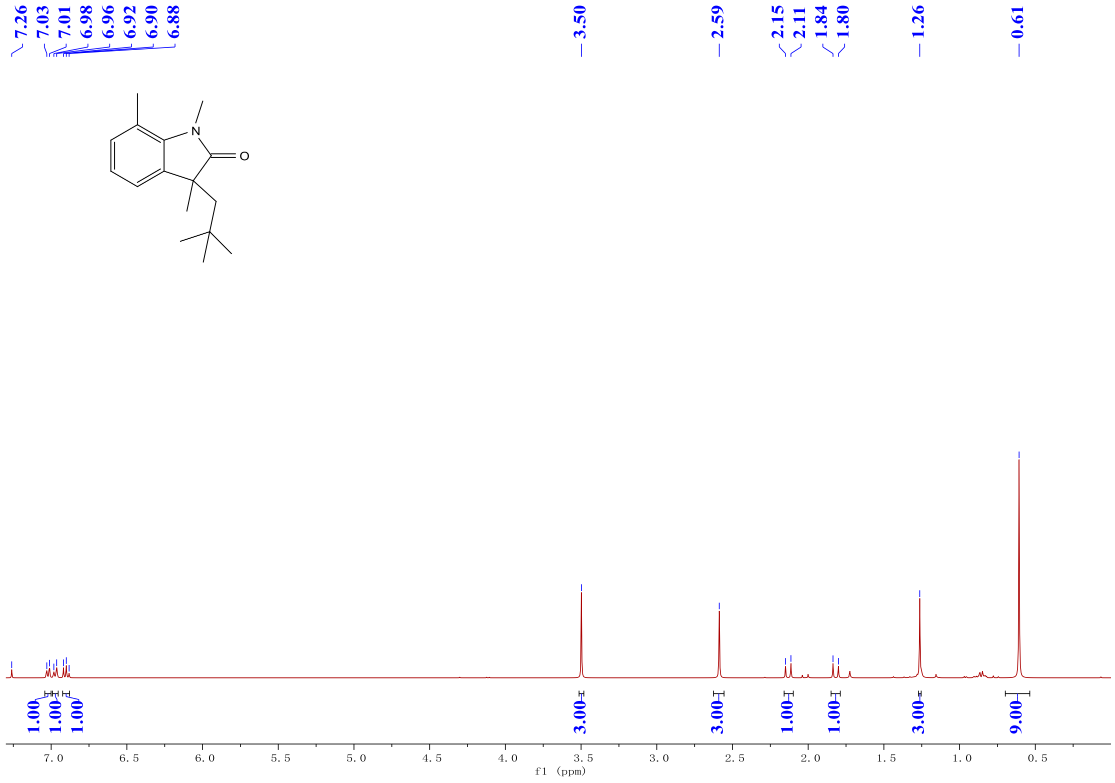

^{1}H NMR spectrum of **3ba** (400 MHz, $CDCl_3$)

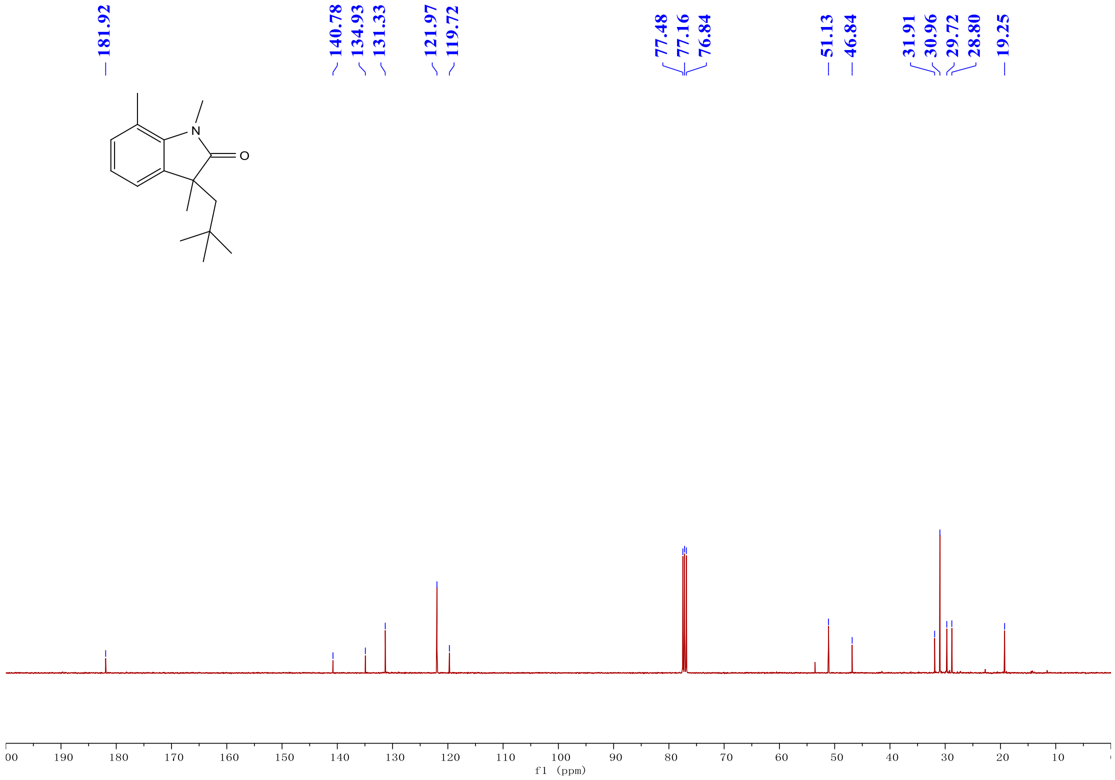

^{13}C {^{1}H} NMR spectrum of Compound **3ba** (101 MHz, $CDCl_3$)

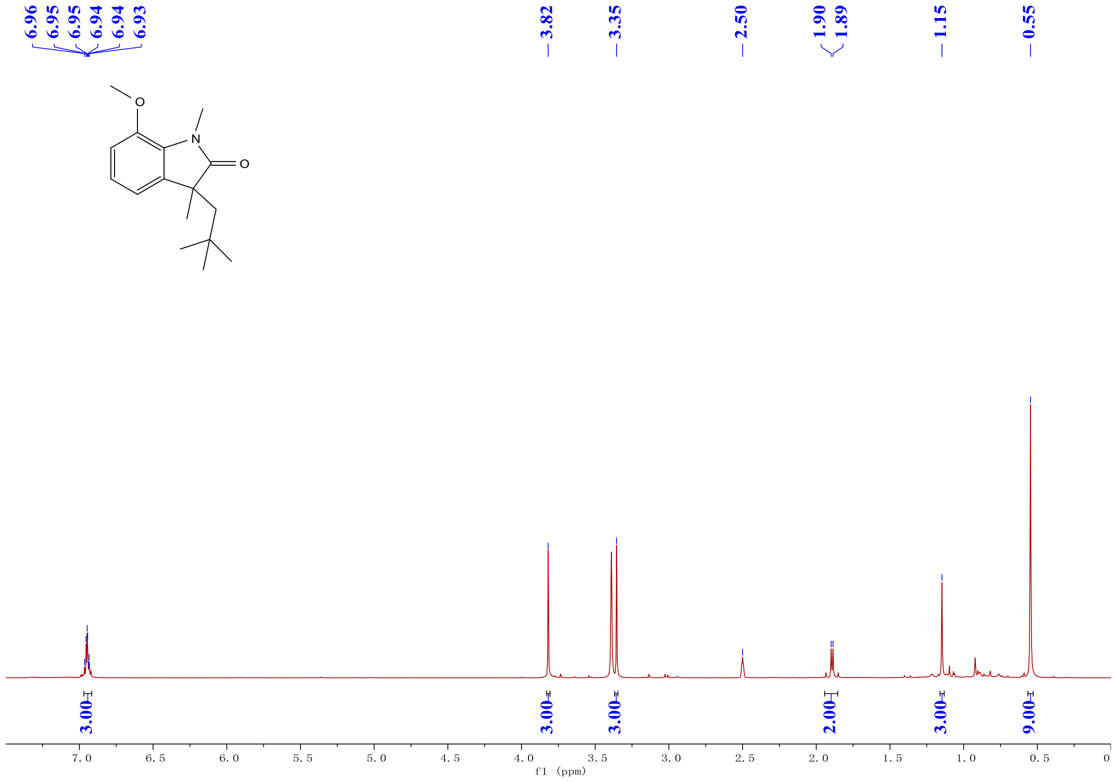

^{1}H NMR spectrum of Compound **3ca** (400 MHz, DMSO-d_6)

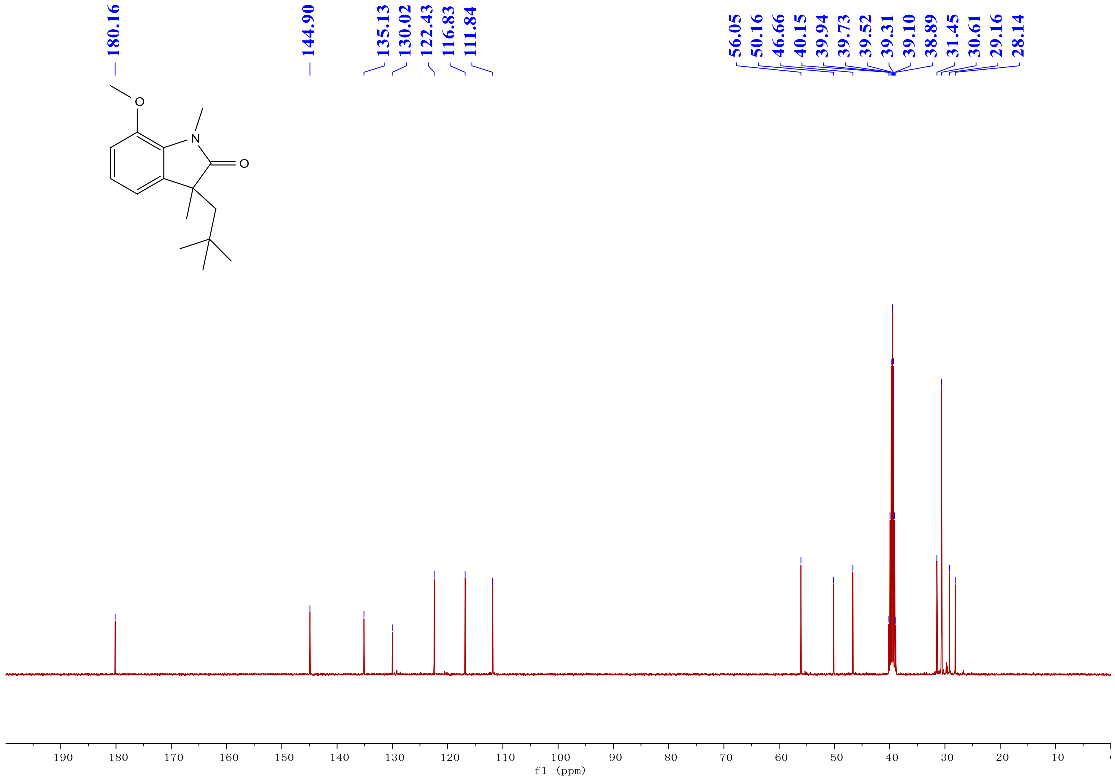

^{13}C {^{1}H} NMR spectrum of Compound **3ca** (101 MHz, DMSO-d_6)

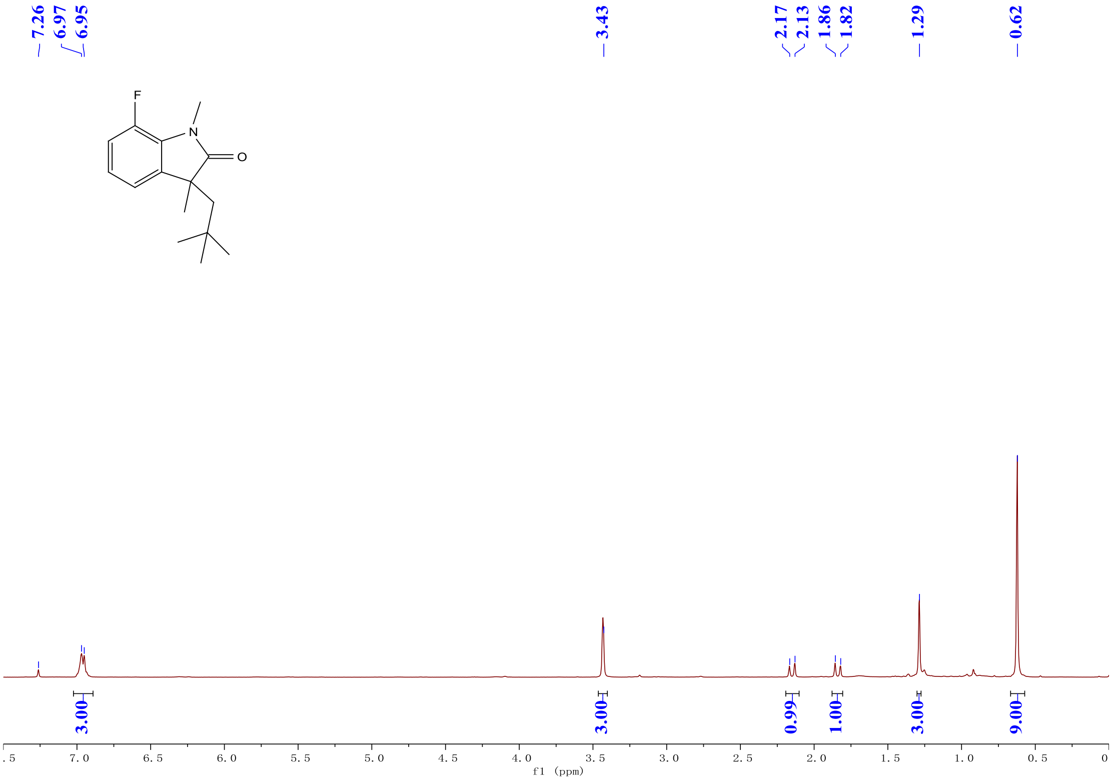

^{1}H NMR spectrum of **3da** (400 MHz, $CDCl_3$)

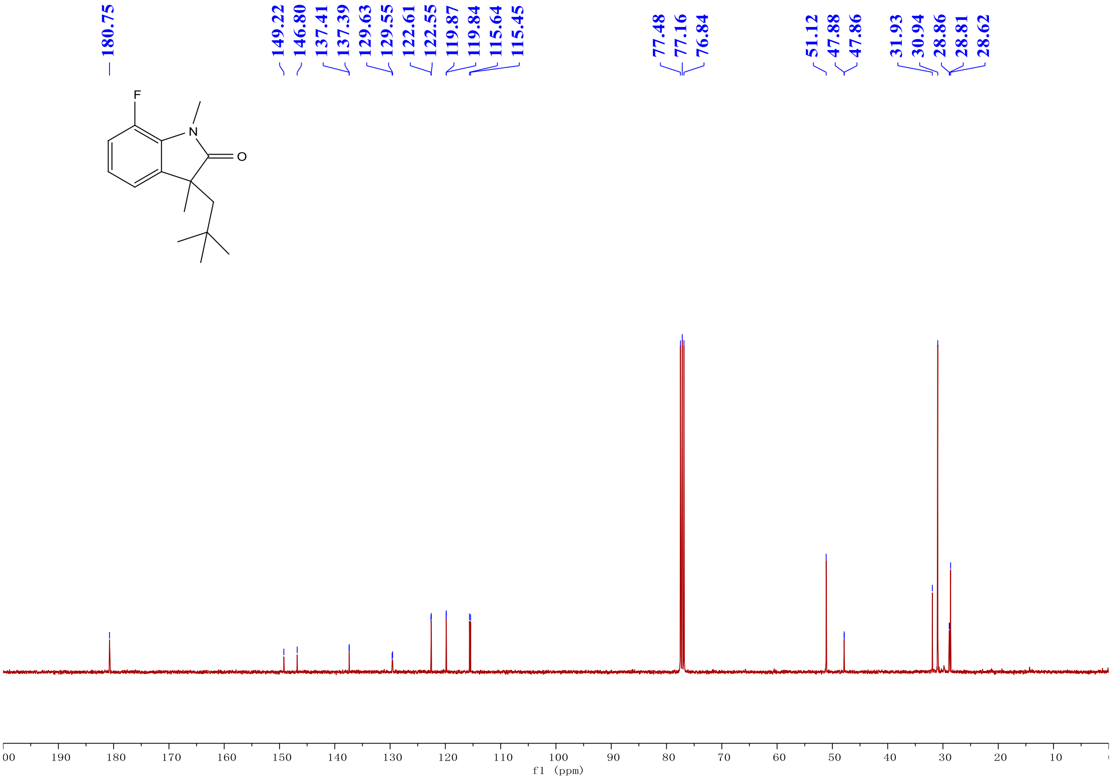

^{13}C {^{1}H} NMR spectrum of Compound **3da** (101 MHz, $CDCl_3$)

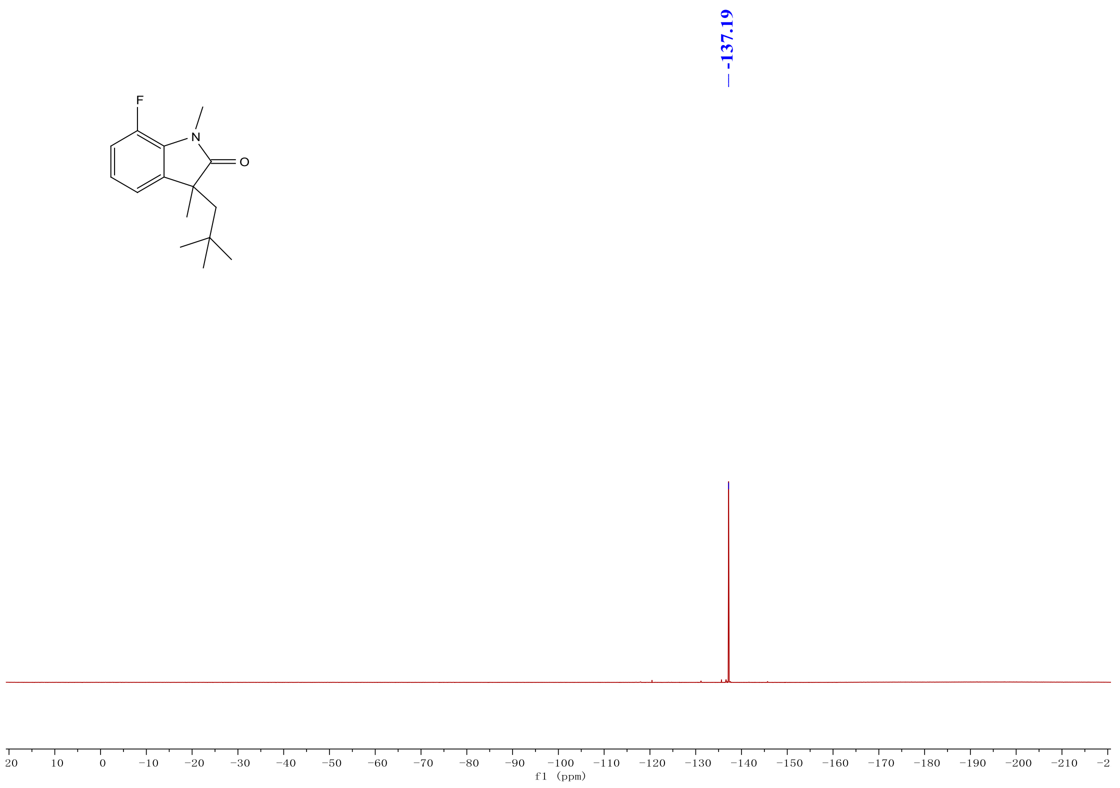

^{19}F NMR spectrum of Compound **3da** (377 MHz, $CDCl_3$)

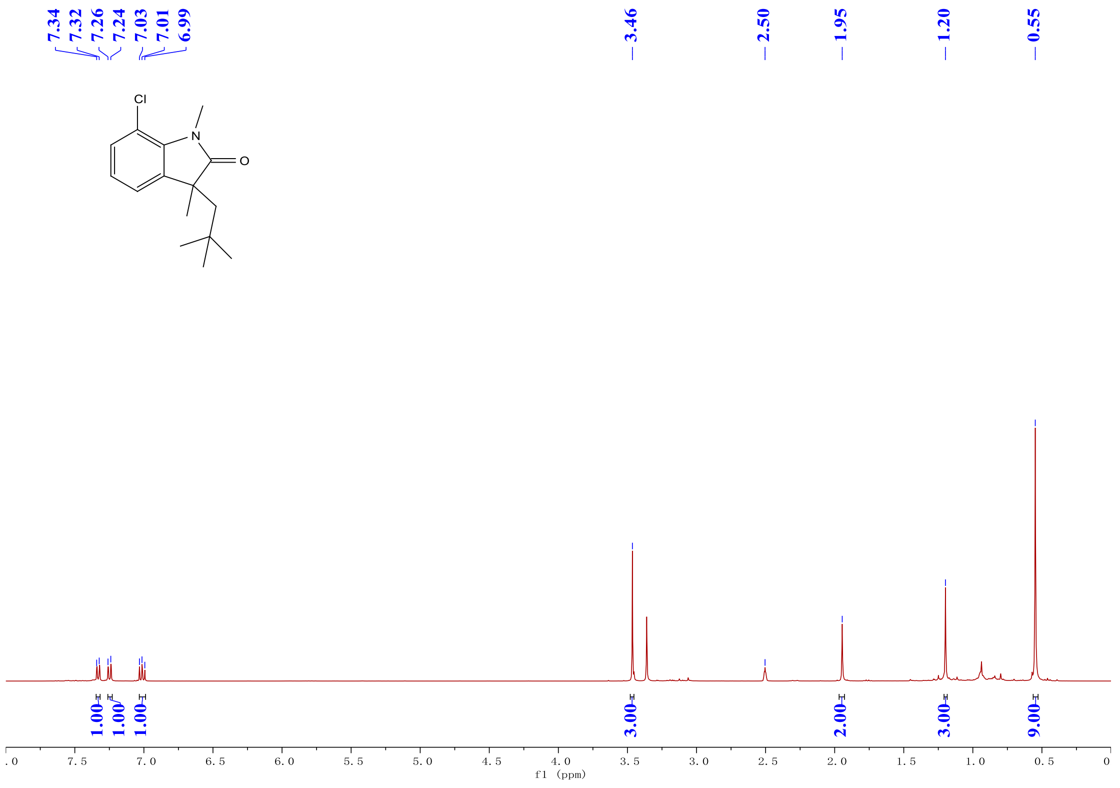

^{1}H NMR spectrum of Compound **3ea** (400 MHz, DMSO-d_6)

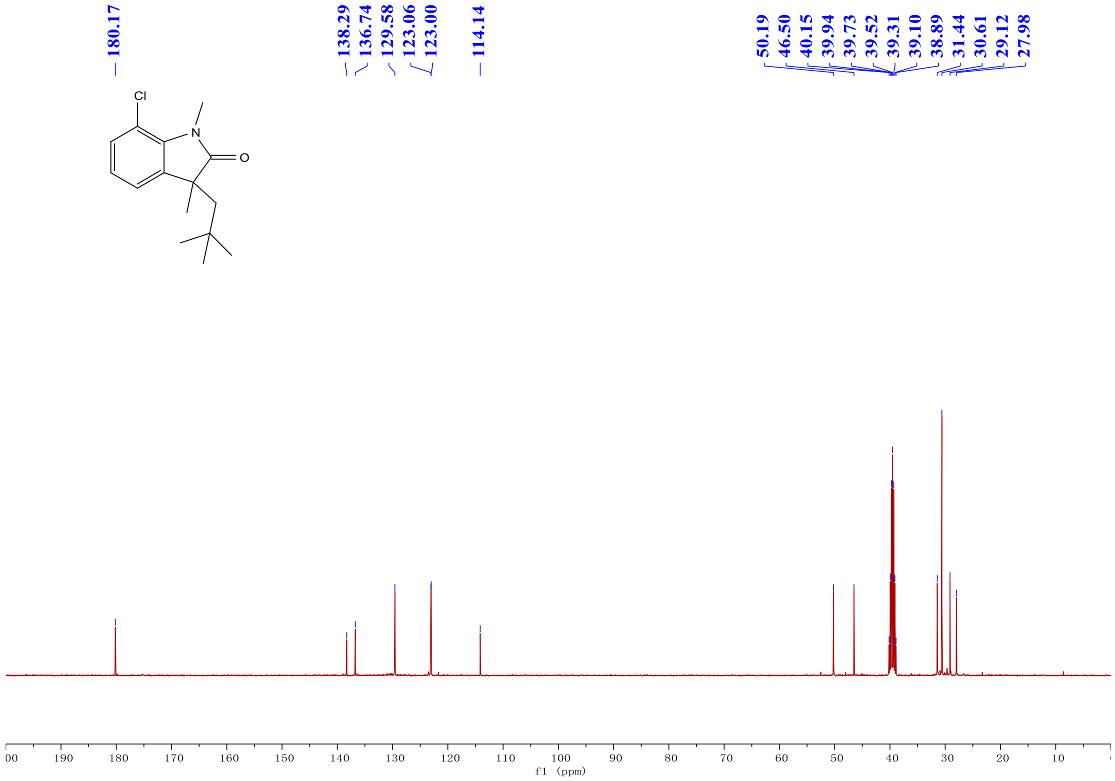

^{13}C {^{1}H} NMR spectrum of Compound **3ea** (101 MHz, DMSO-d_6)

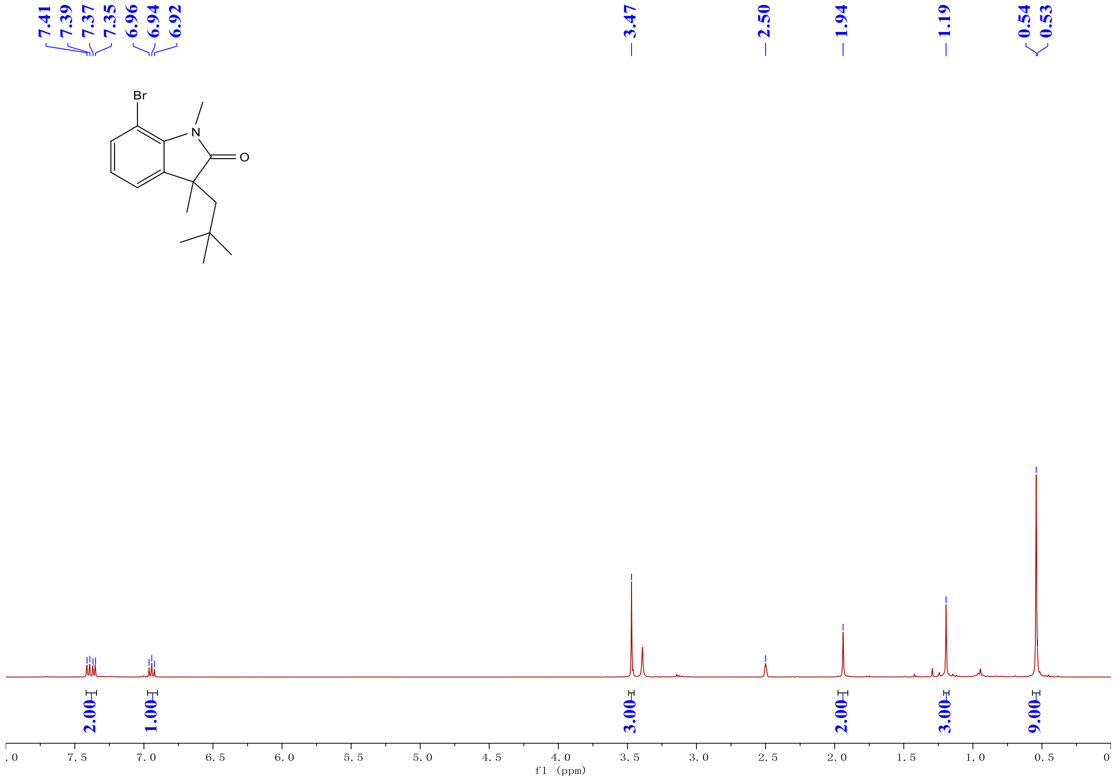

^{1}H NMR spectrum of Compound **3fa** (400 MHz, DMSO-d_6)

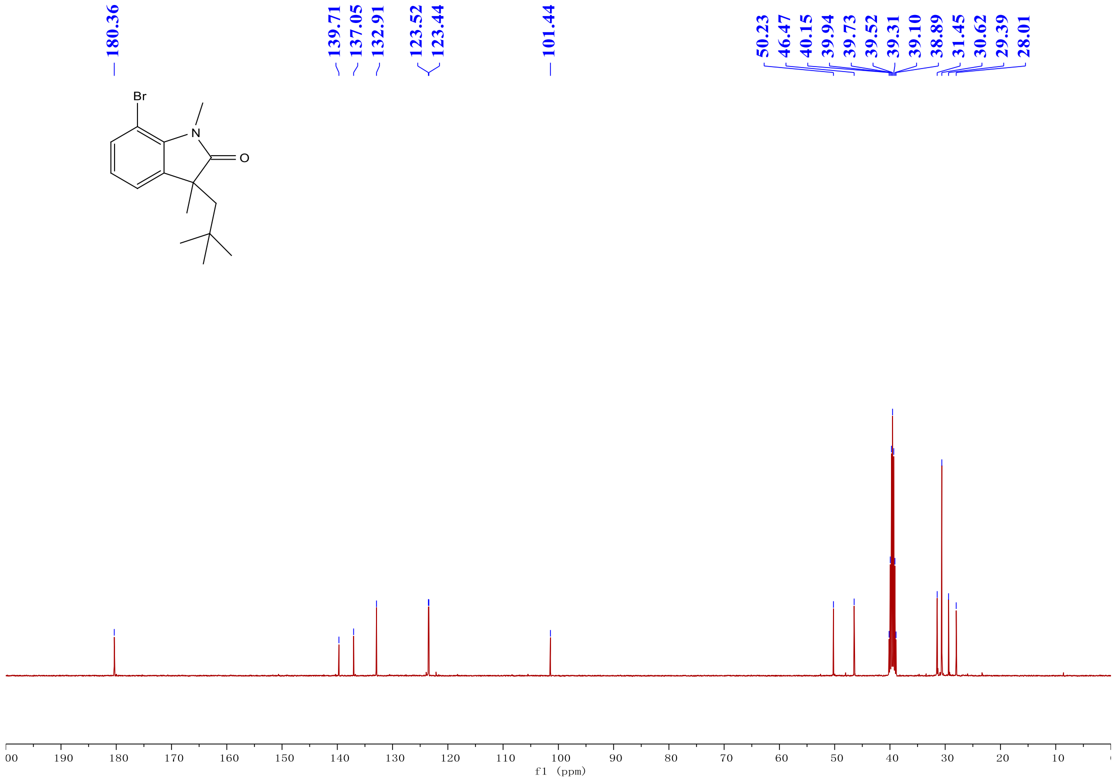

^{13}C {^{1}H} NMR spectrum of Compound **3fa** (101 MHz, DMSO-d_6)

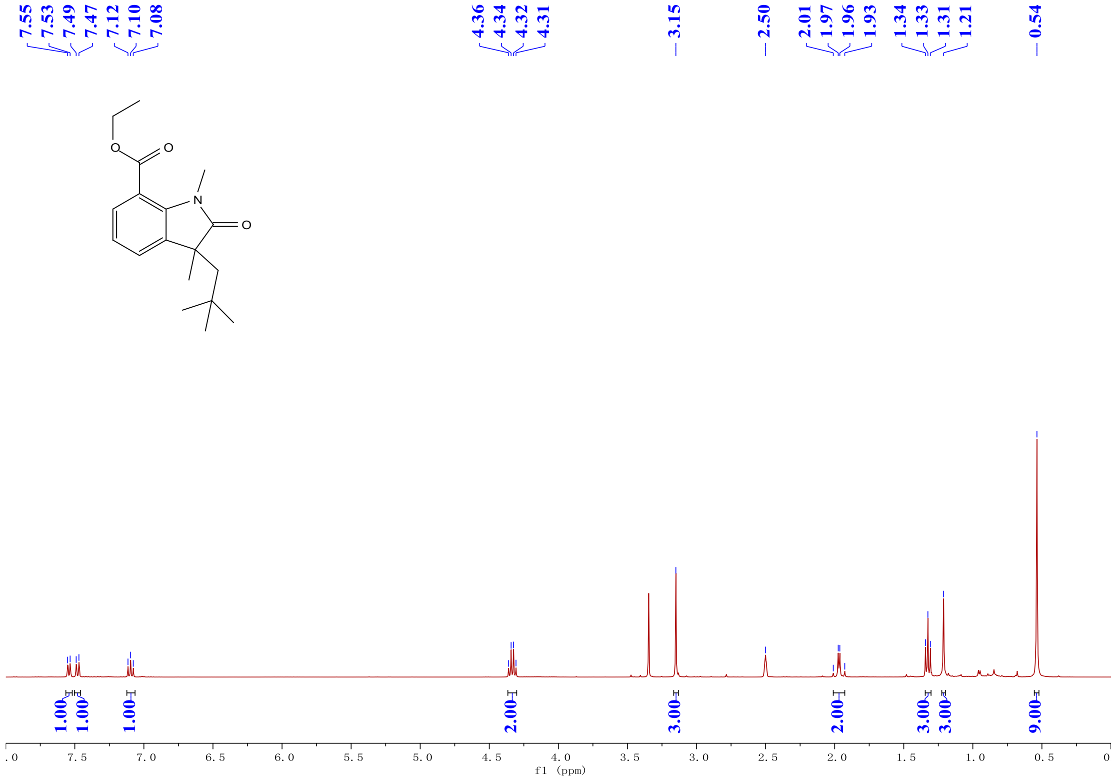

^{1}H NMR spectrum of Compound **3ga** (400 MHz, DMSO-d_6)

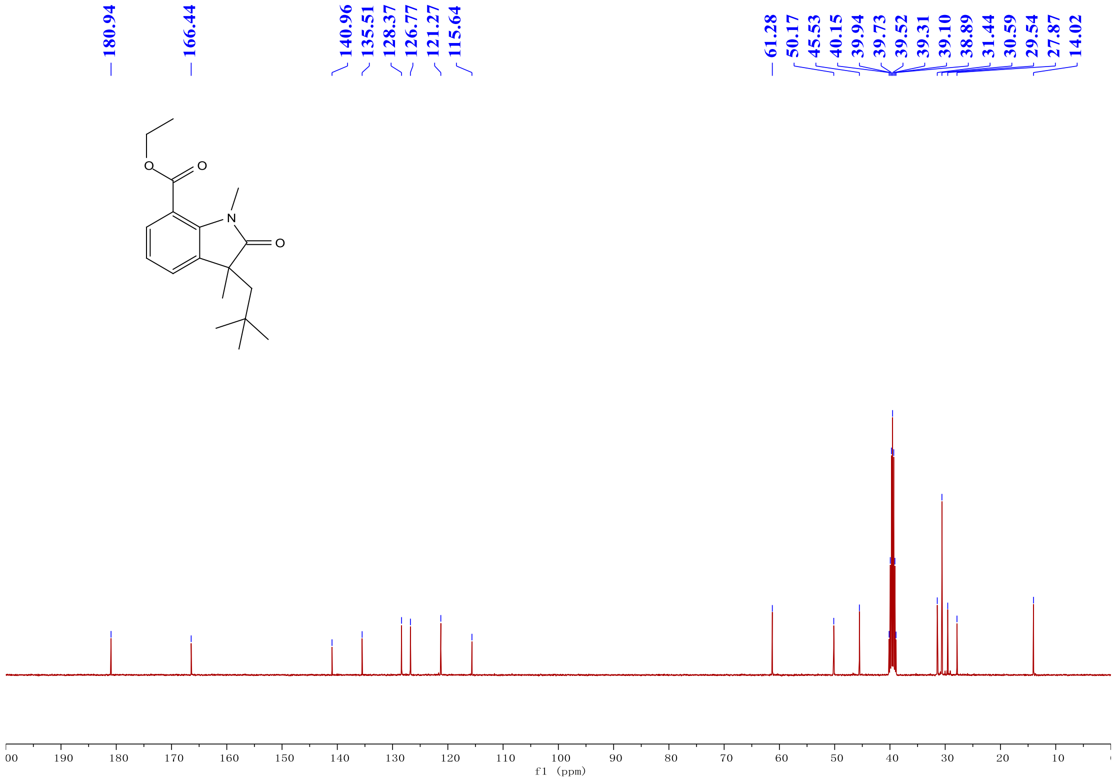

^{13}C {^{1}H} NMR spectrum of Compound **3ga** (101 MHz, DMSO-d_6)

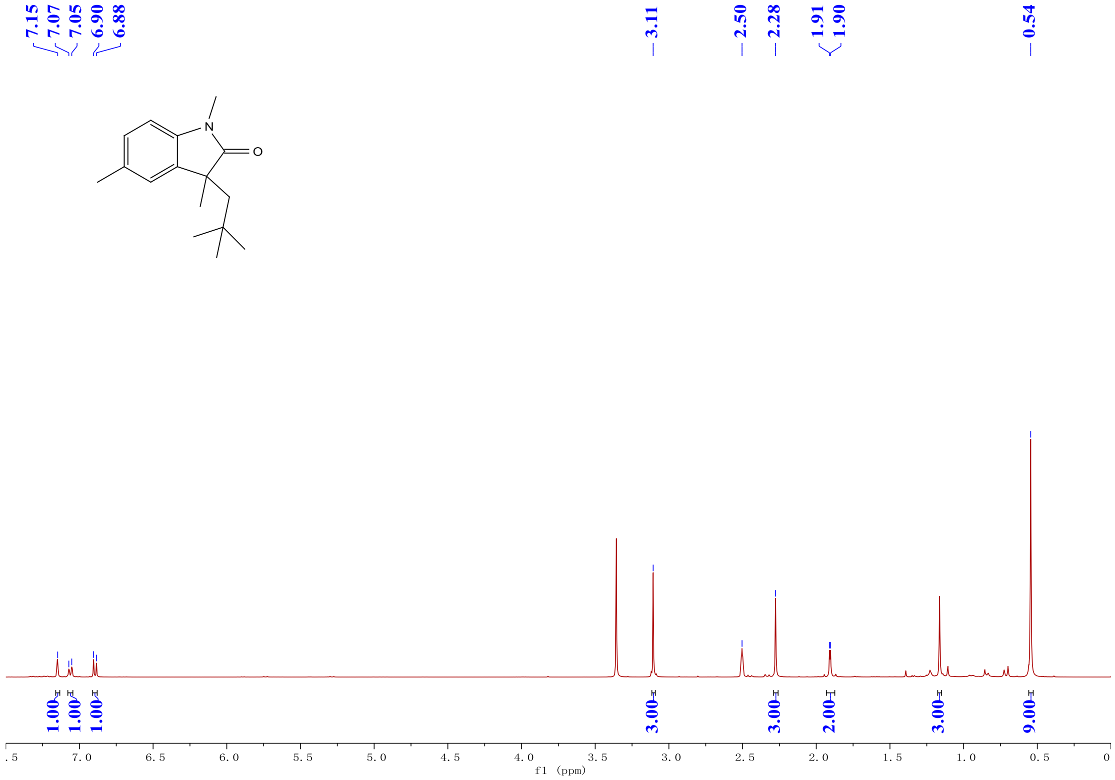

^{1}H NMR spectrum of Compound **3ha** (400 MHz, DMSO-d_6)

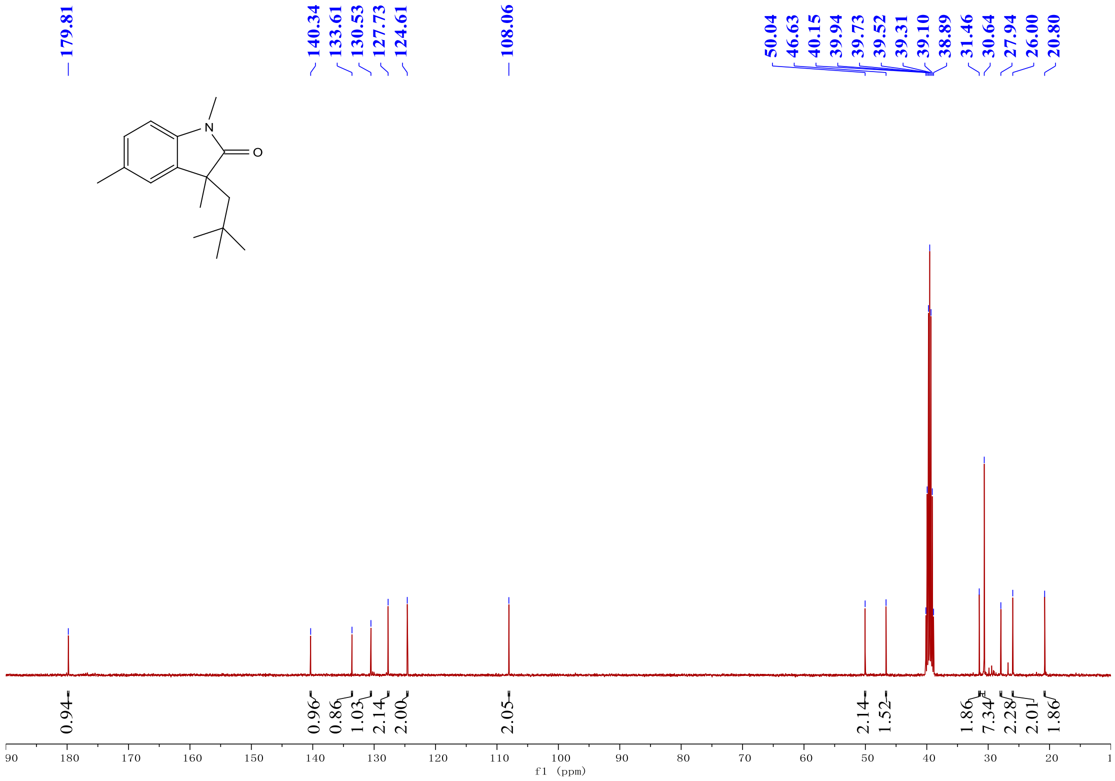

^{13}C {^{1}H} NMR spectrum of Compound **3ha** (101 MHz, DMSO-d_6)

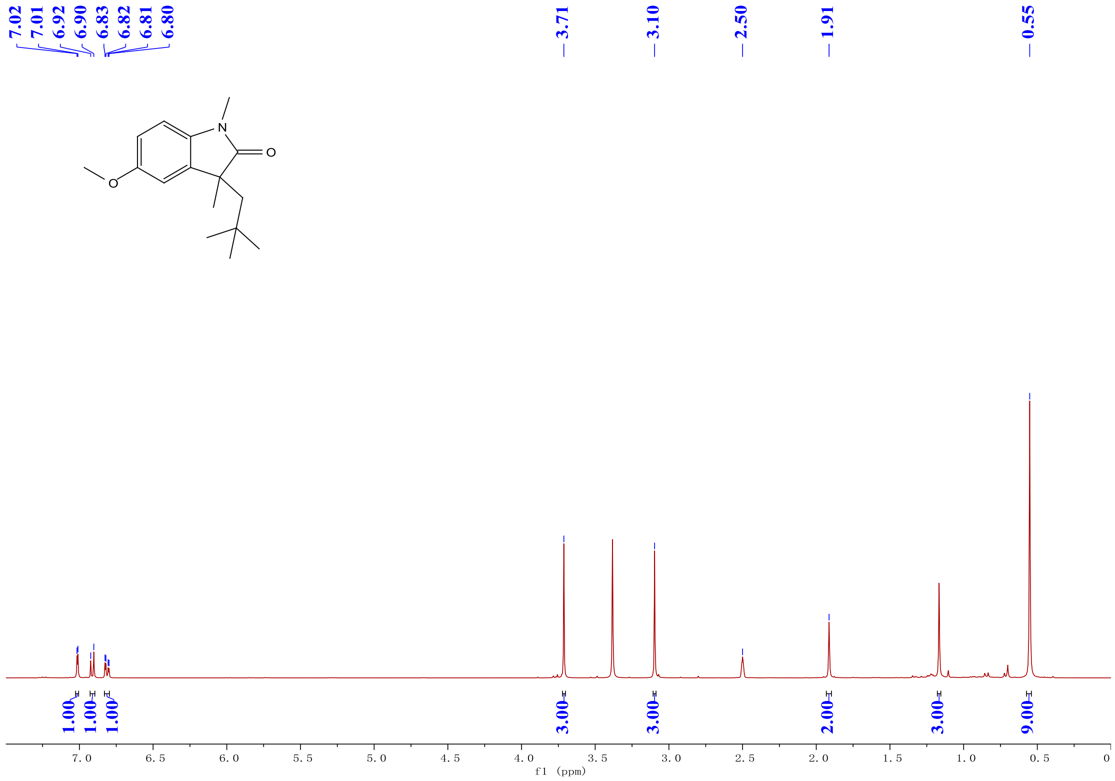

^{1}H NMR spectrum of Compound **3ia** (400 MHz, DMSO-d_6)

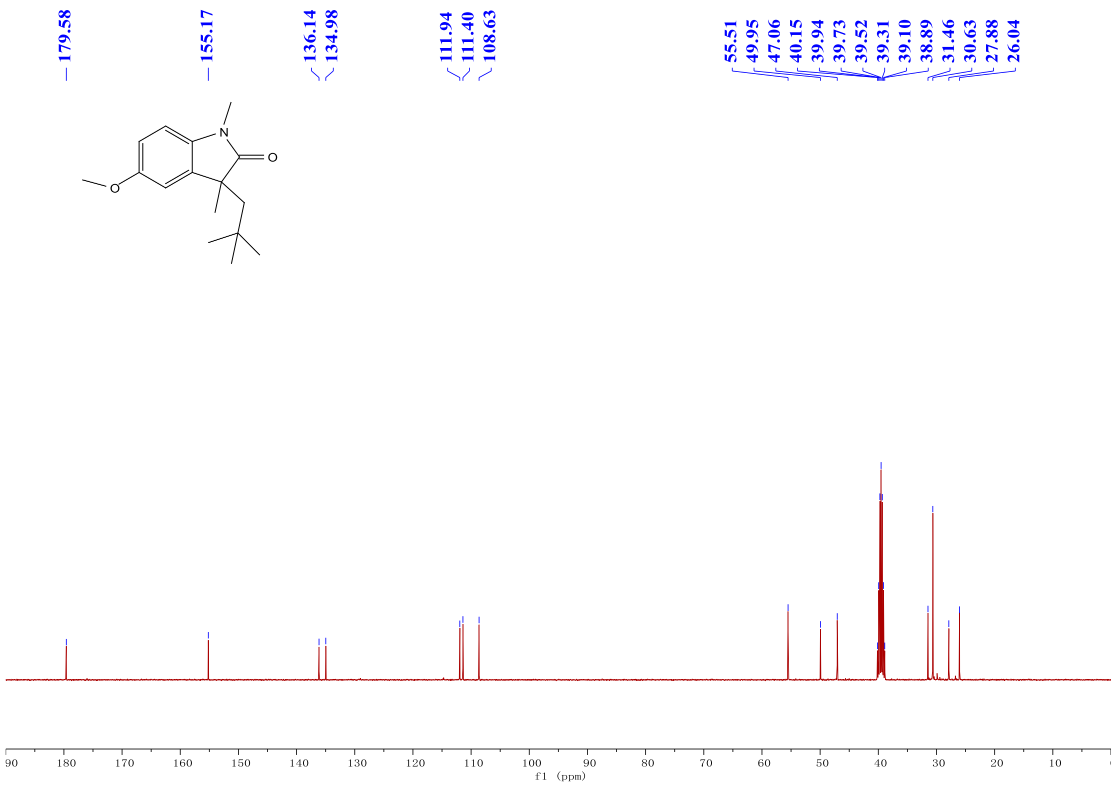

^{13}C {^1H} NMR spectrum of Compound **3ia** (101 MHz, DMSO-d_6)

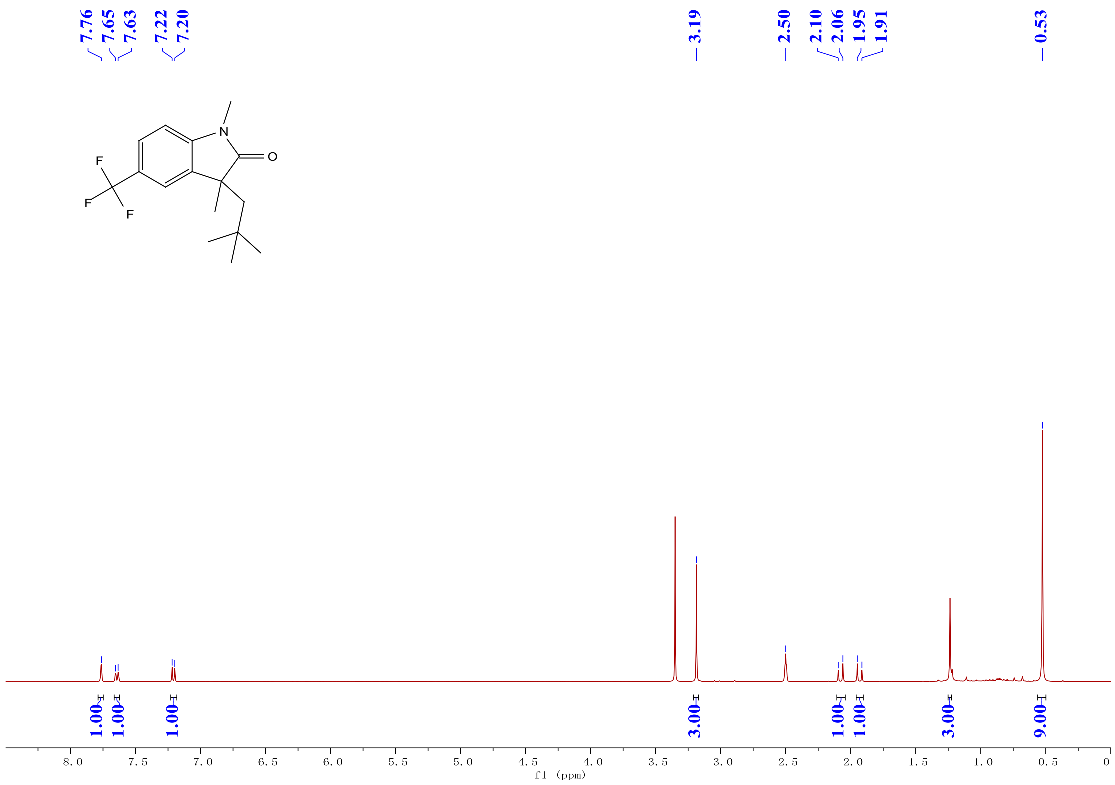

^1H NMR spectrum of Compound **3ja** (400 MHz, DMSO-d_6)

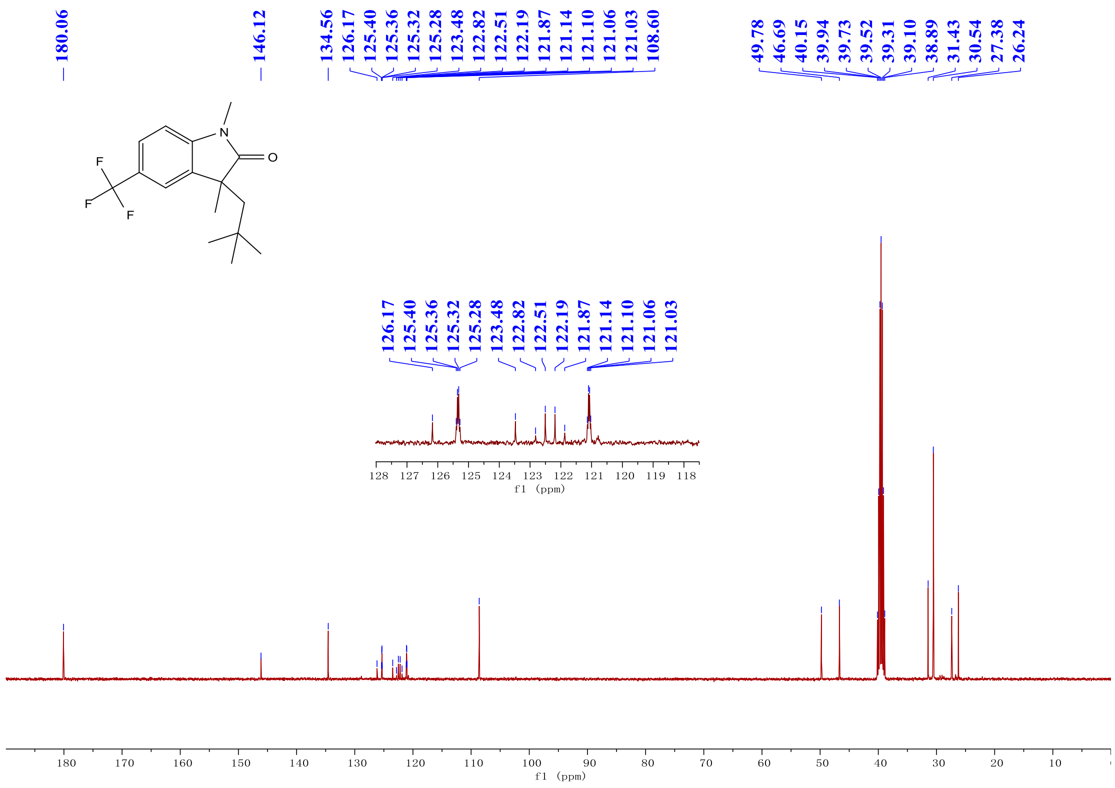

^{13}C {^{1}H} NMR spectrum of Compound **3ja** (101 MHz, DMSO-d_6)

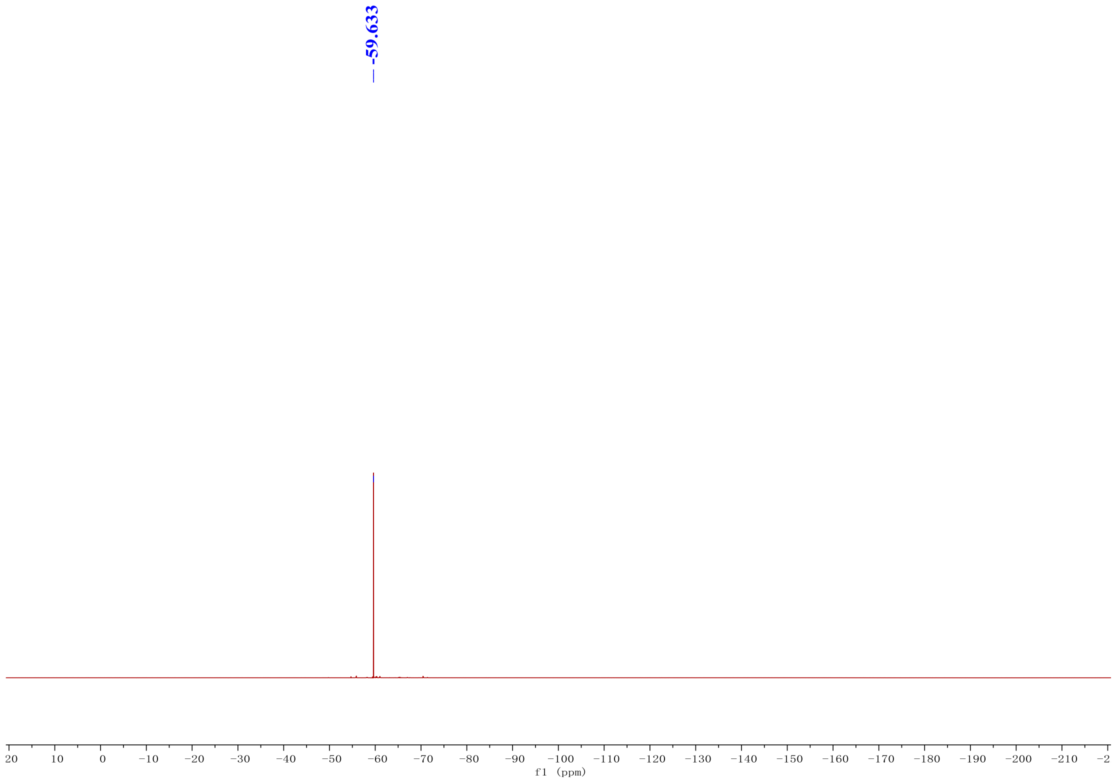

^{19}F NMR spectrum of Compound **3ja** (377 MHz, $CDCl_3$)

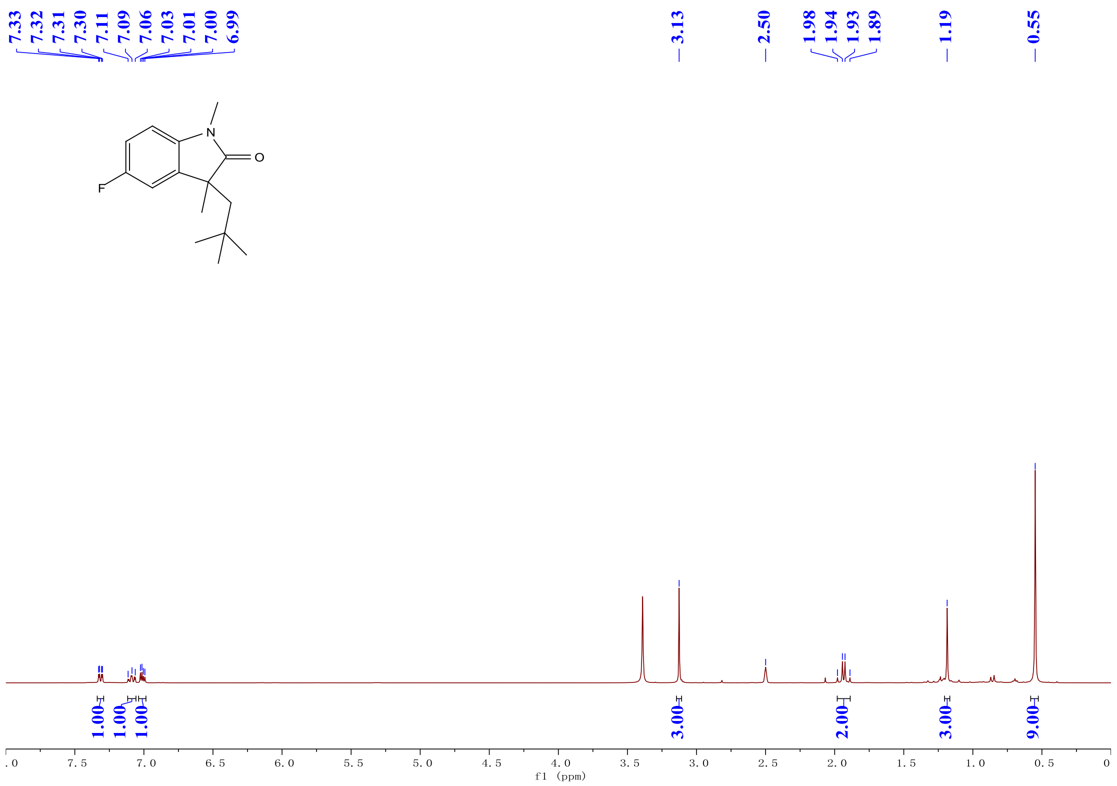

^{1}H NMR spectrum of Compound **3ka** (400 MHz, DMSO-d_6)

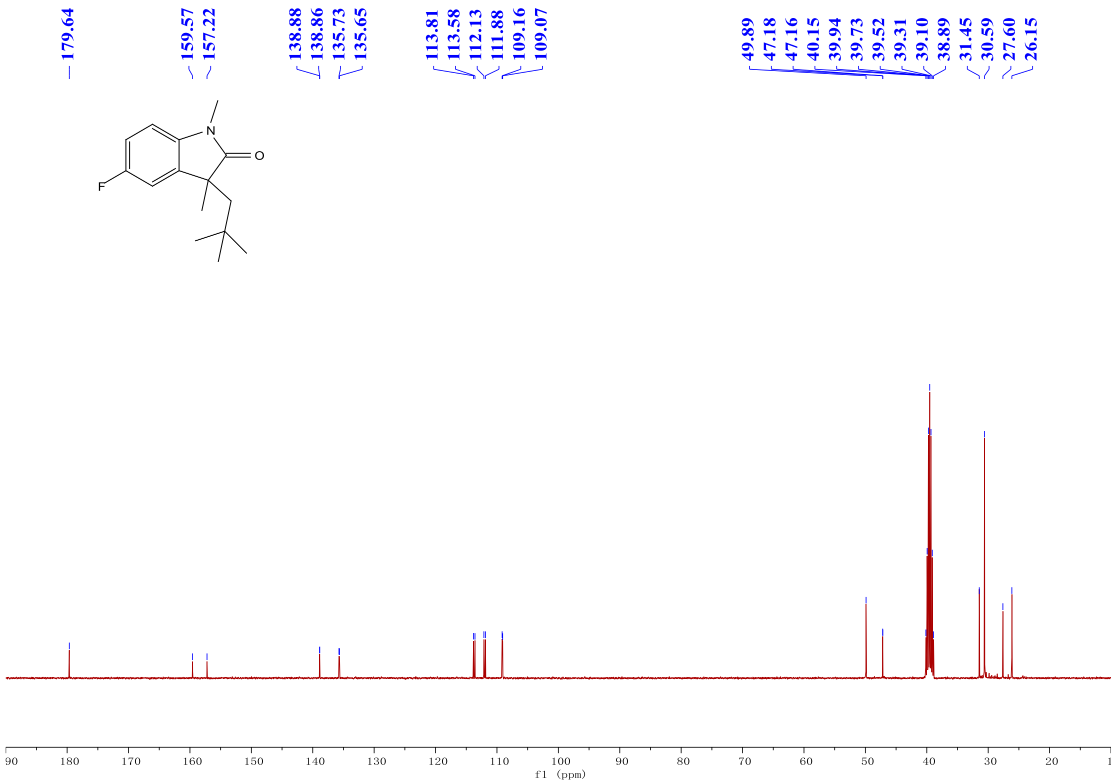

^{13}C {^{1}H} NMR spectrum of Compound **3ka** (101 MHz, DMSO-d_6)

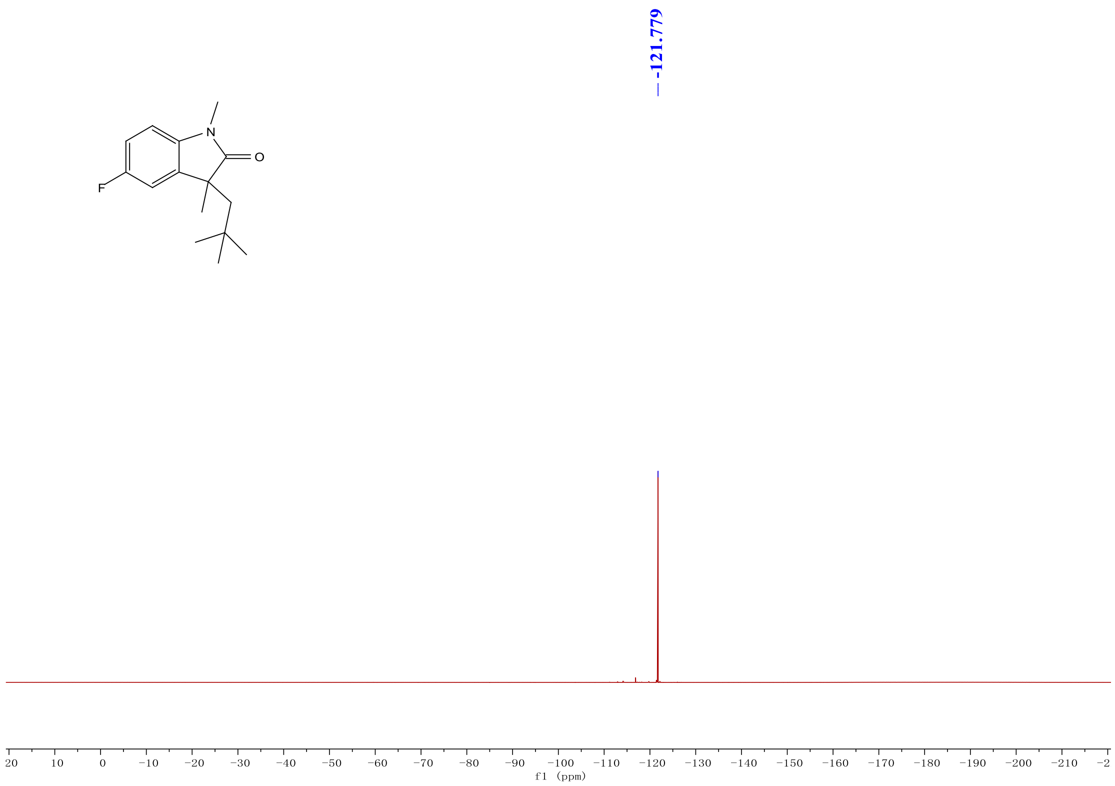

^{19}F NMR spectrum of Compound **3ka** (377 MHz, DMSO-d_6)

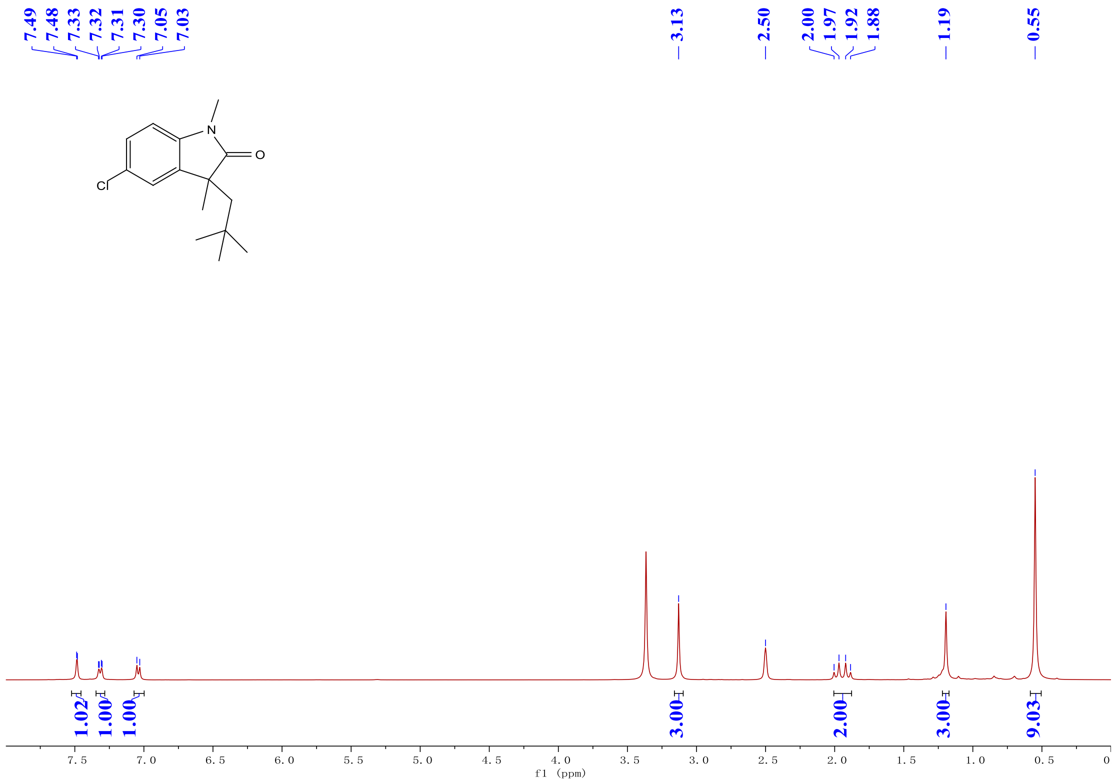

^{1}H NMR spectrum of Compound **3la** (400 MHz, DMSO-d_6)

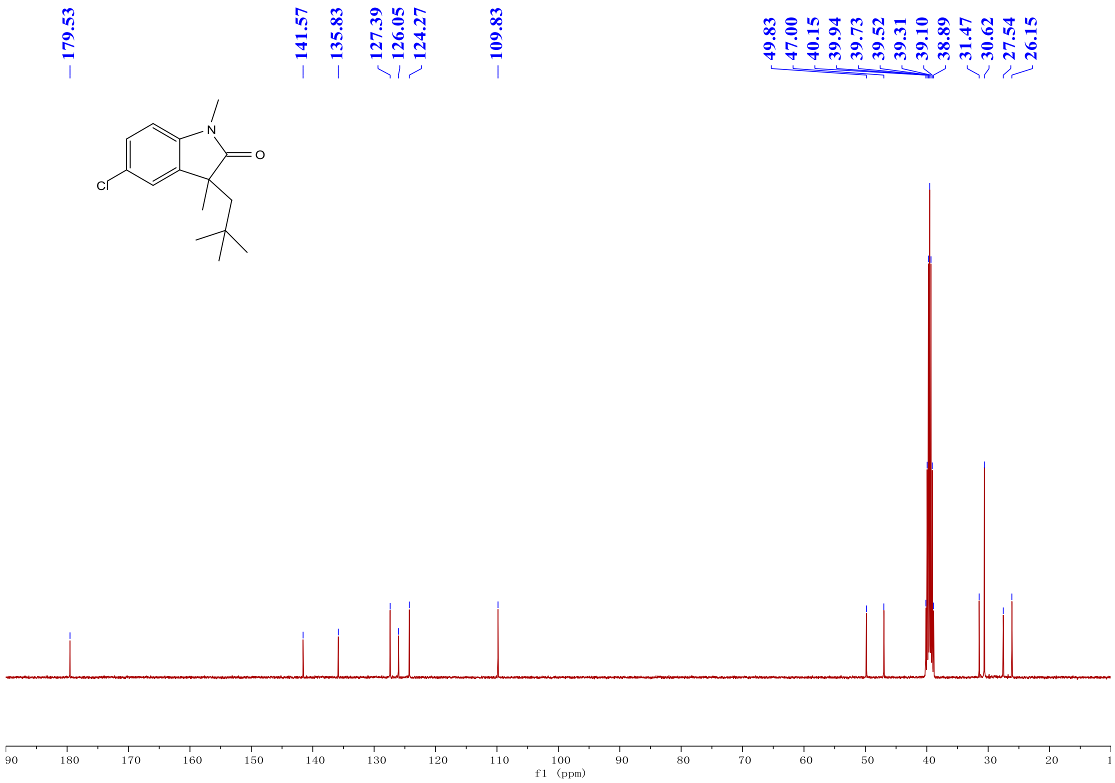

^{13}C {^1H} NMR spectrum of **3la** (101 MHz, DMSO-d_6)

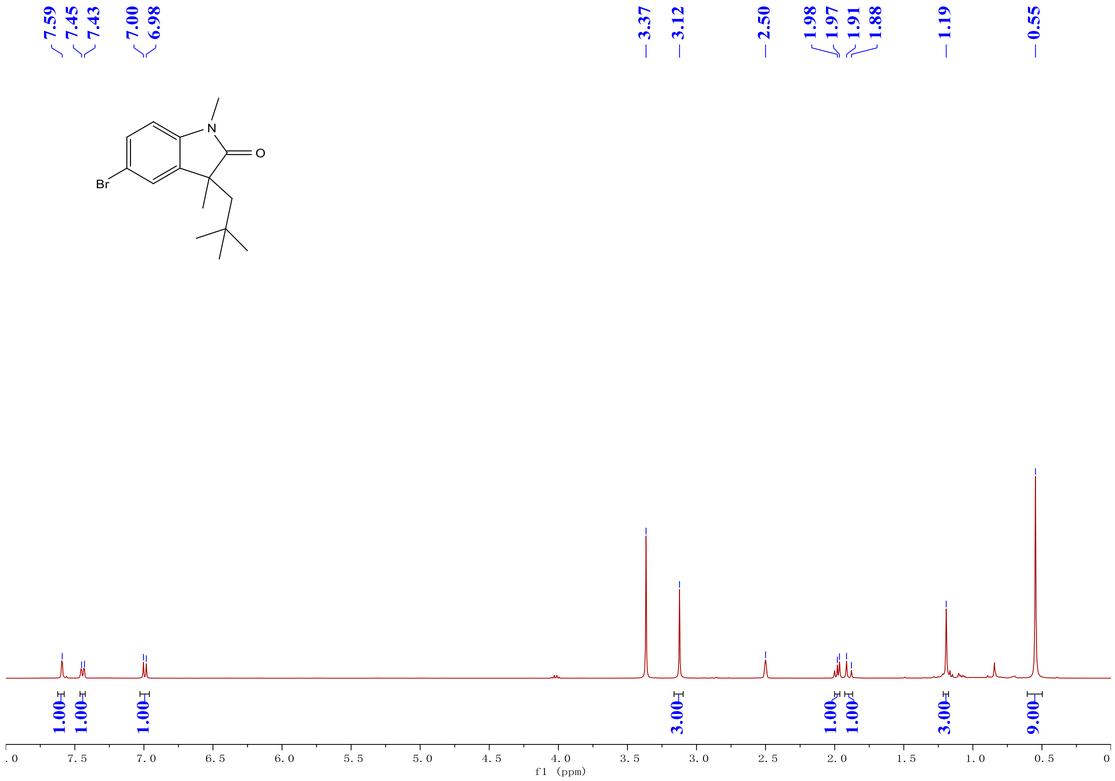

^1H NMR spectrum of **3ma** (400 MHz, DMSO-d_6)

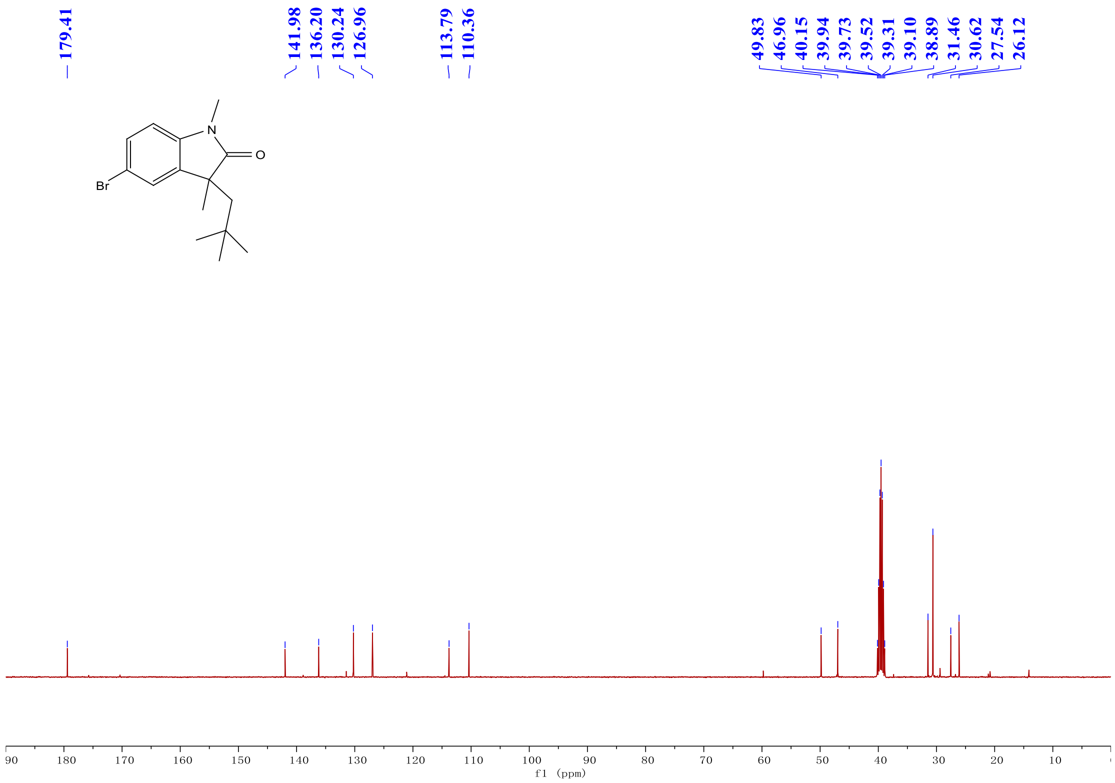

^{13}C {^{1}H} NMR spectrum of **3ma** (101 MHz, DMSO-d_6)

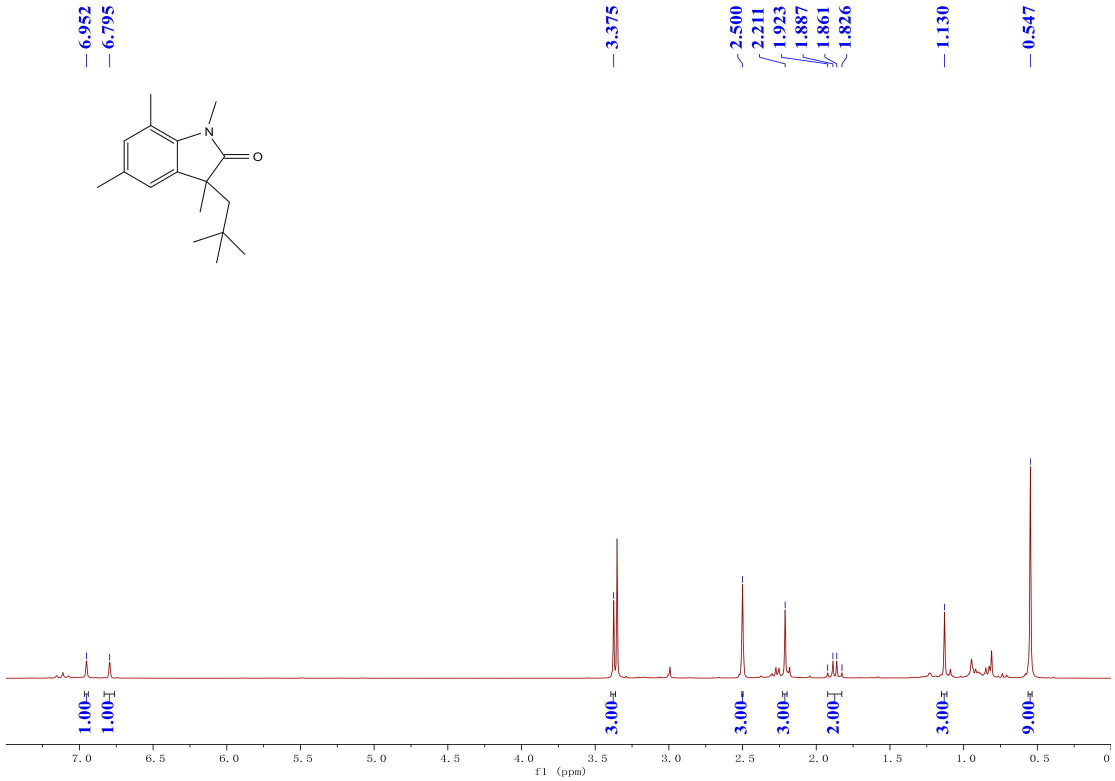

^{1}H NMR spectrum of **3oa** (400 MHz, DMSO-d_6)

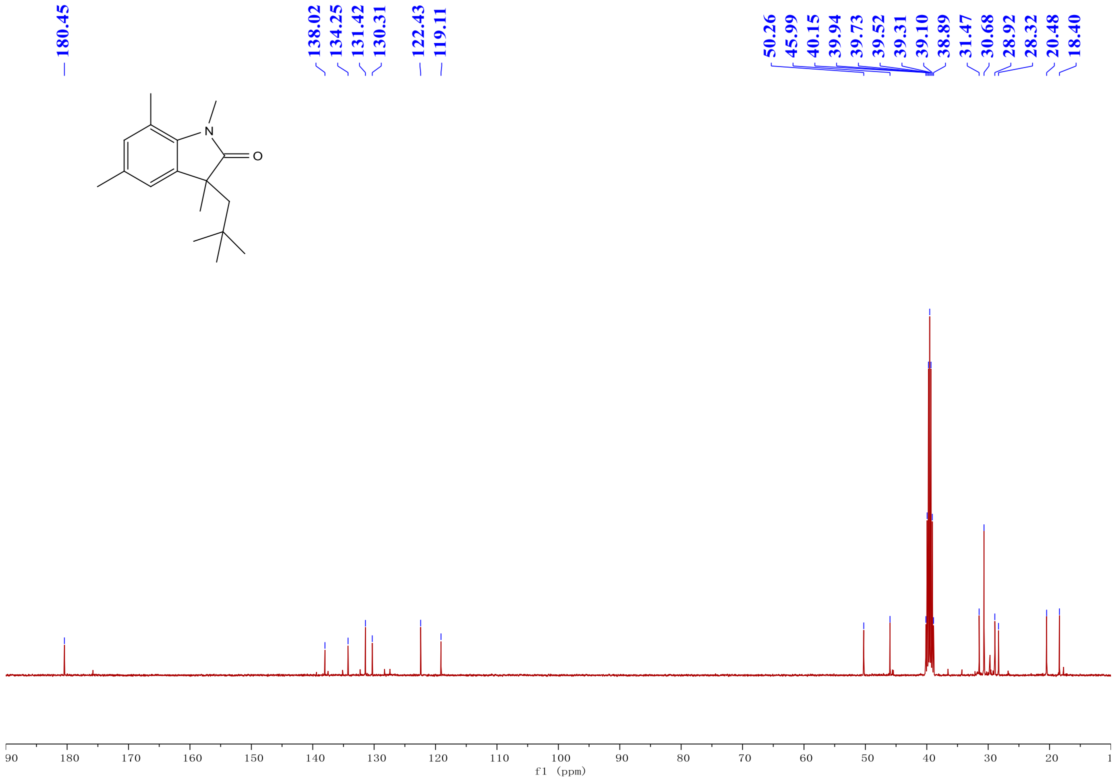

^{13}C {^{1}H} NMR spectrum of **3oa** (101 MHz, DMSO-d_6)

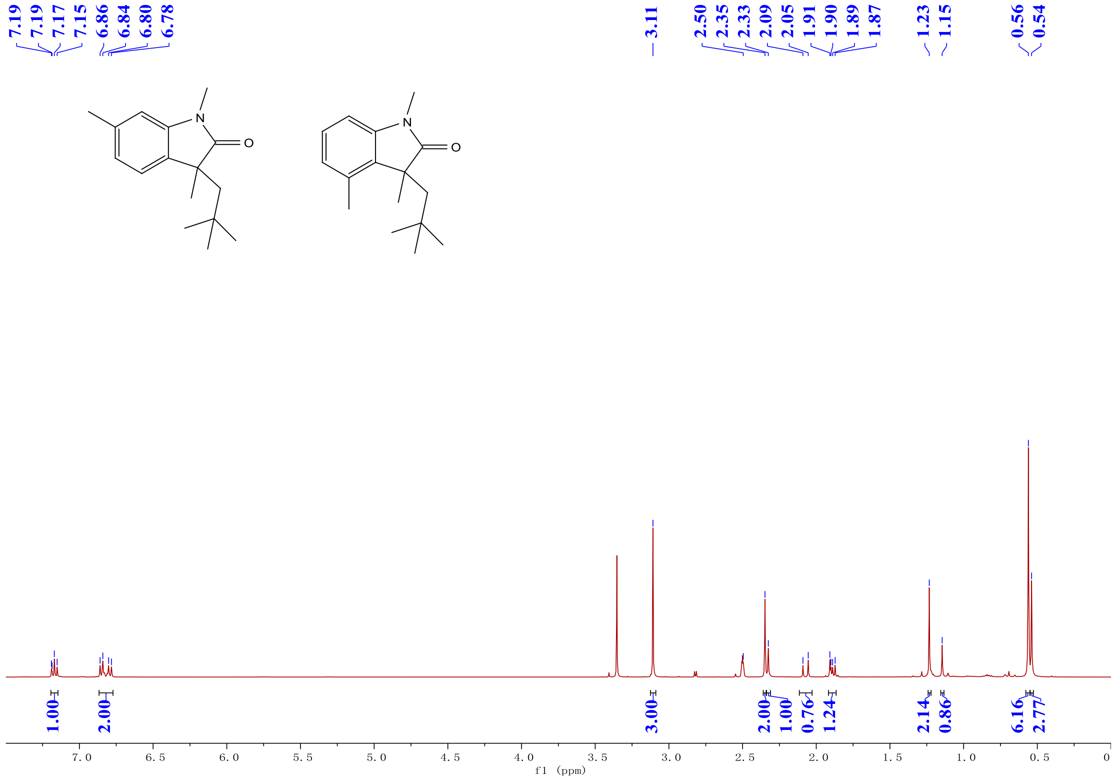

^{1}H NMR spectrum of **3pa+3pa'** (400 MHz, DMSO-d_6)

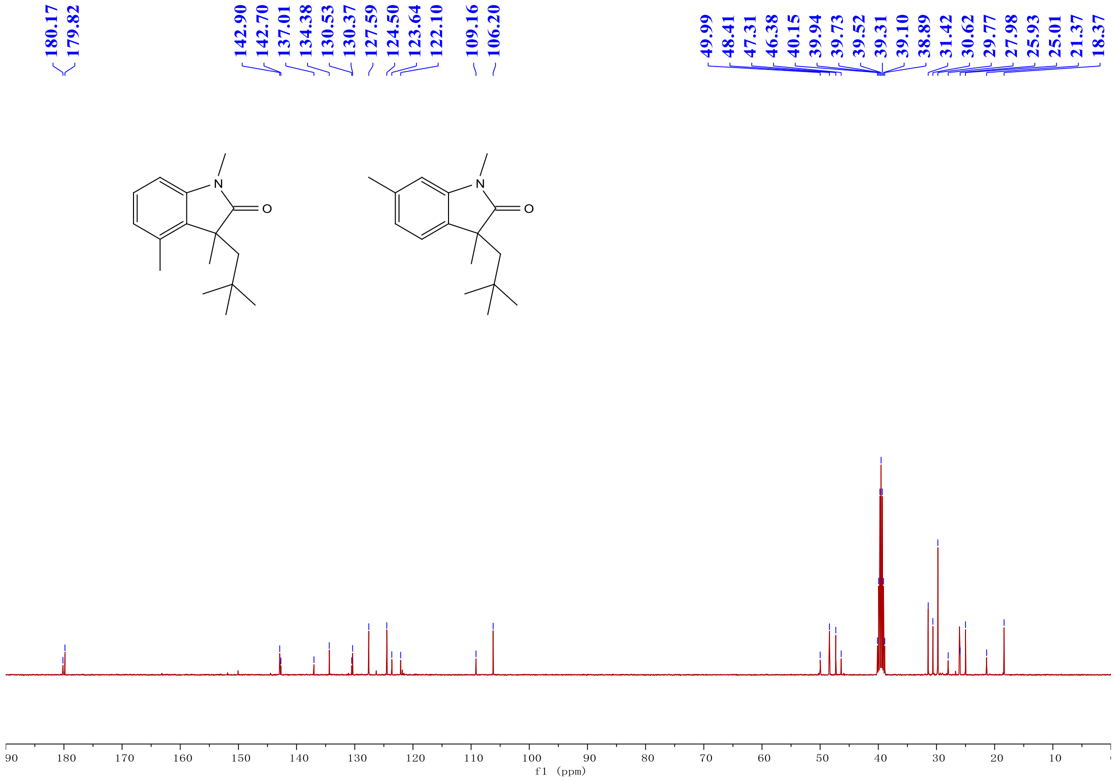

^{13}C {^{1}H} NMR spectrum of **3pa+3pa'** (101 MHz, DMSO-d_6)

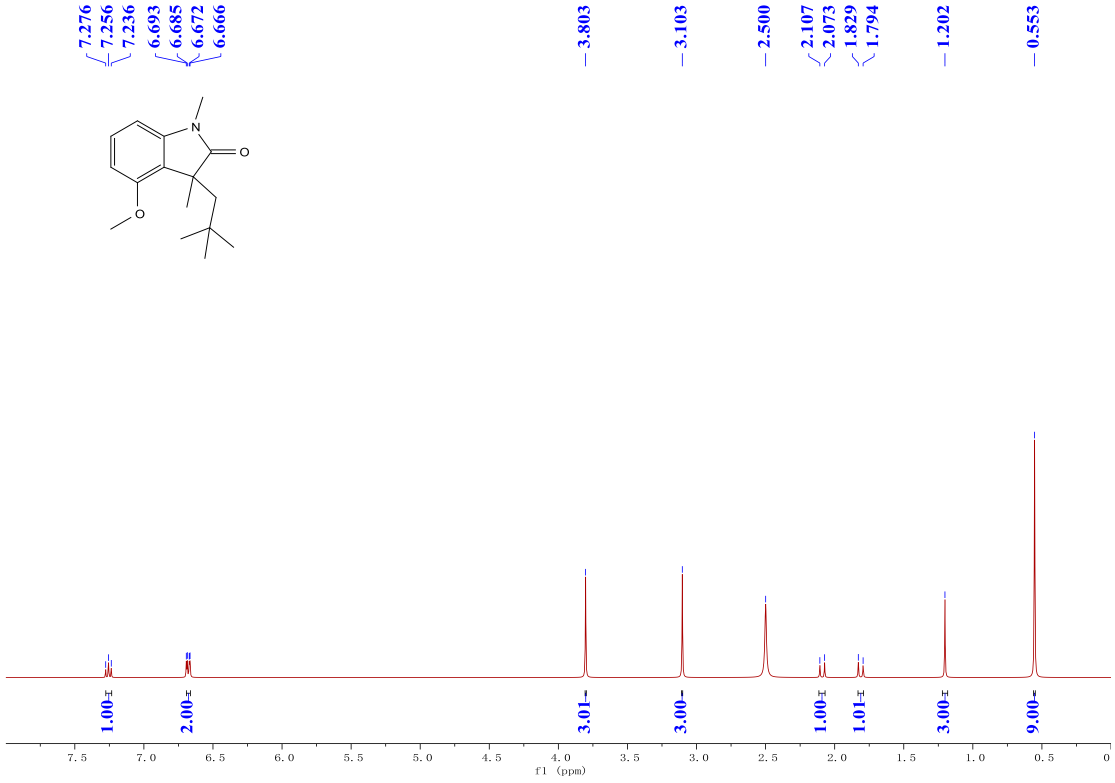

^{1}H NMR spectrum of **3qa** (400 MHz, DMSO-d_6)

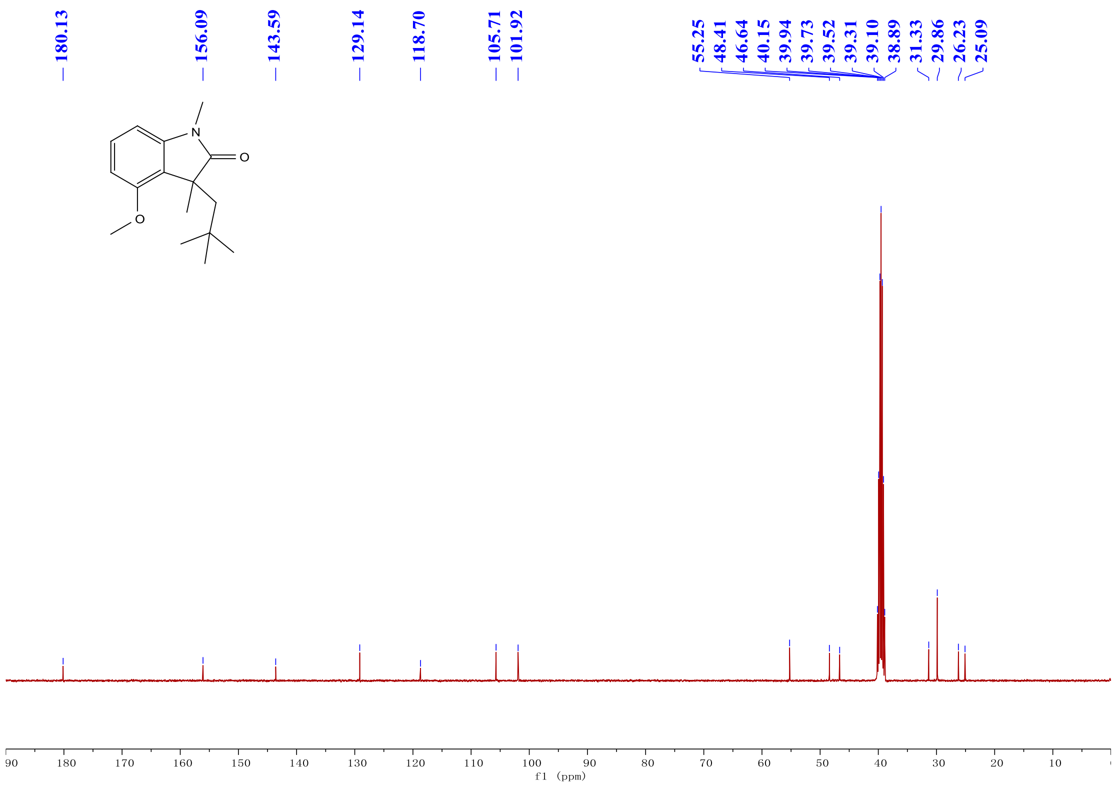

^{13}C {^{1}H} NMR spectrum of **3qa** (101 MHz, DMSO-d_6)

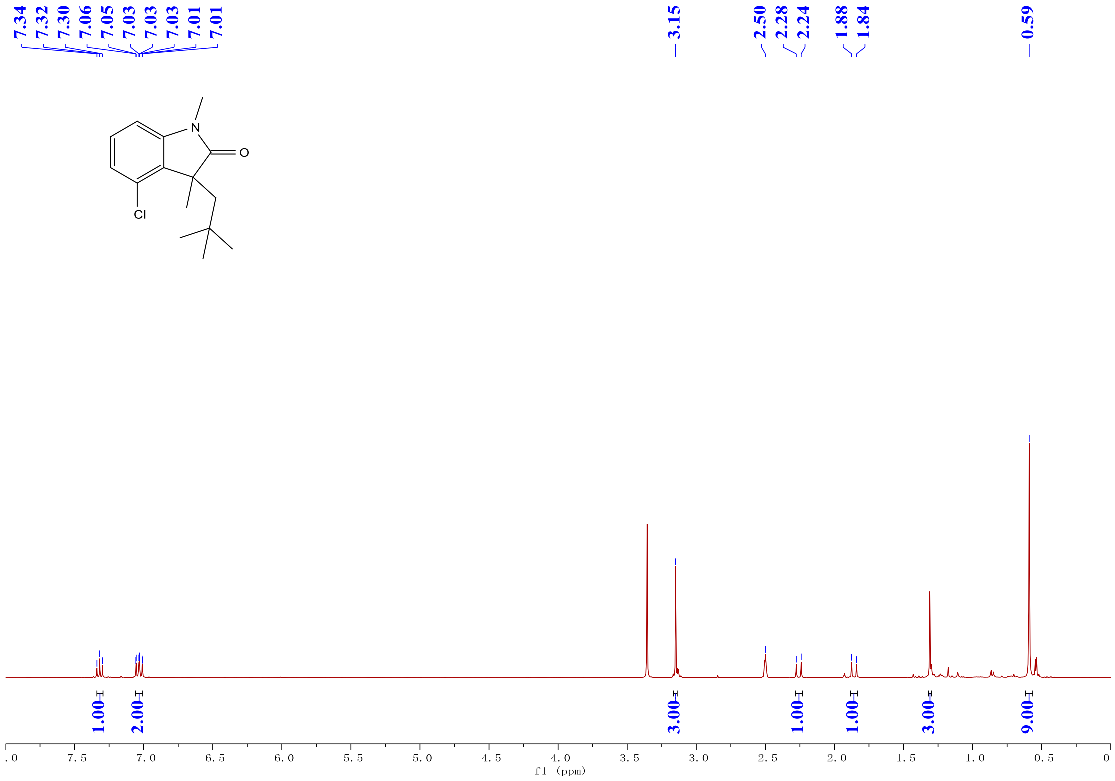

^{1}H NMR spectrum of **3ra** (400 MHz, DMSO-d_6)

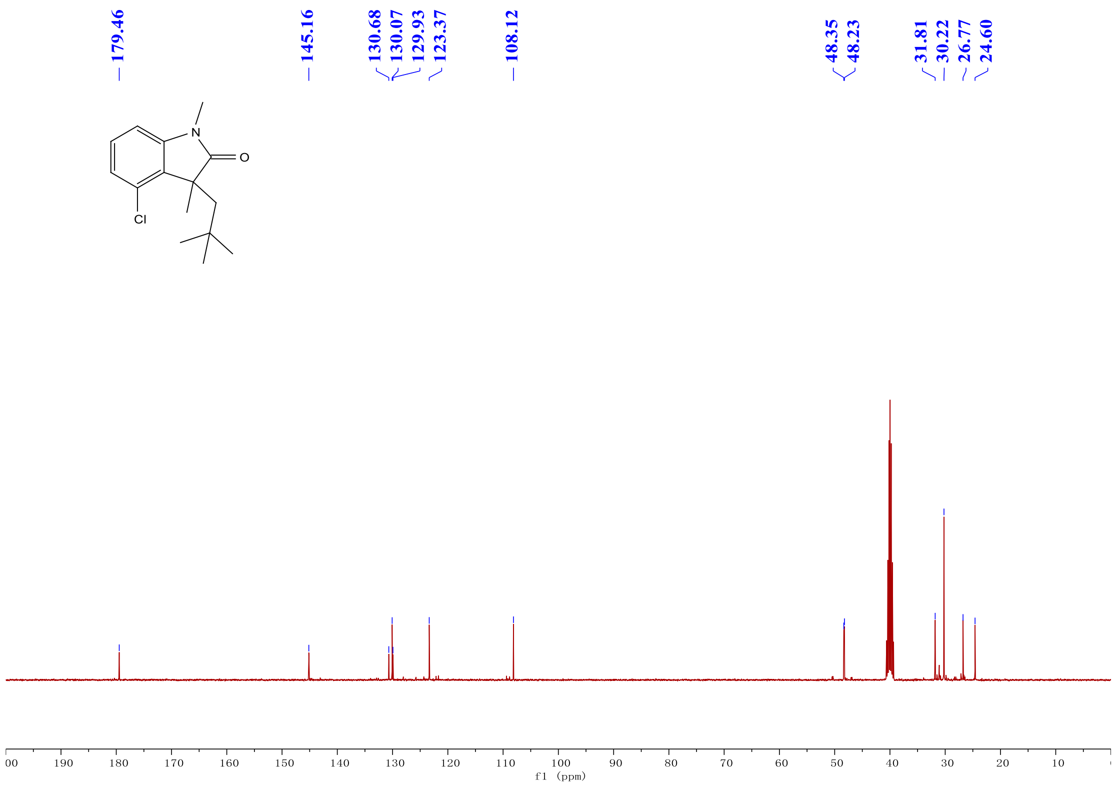

^{13}C {^{1}H} NMR spectrum of **3ra** (101 MHz, DMSO-d_6)

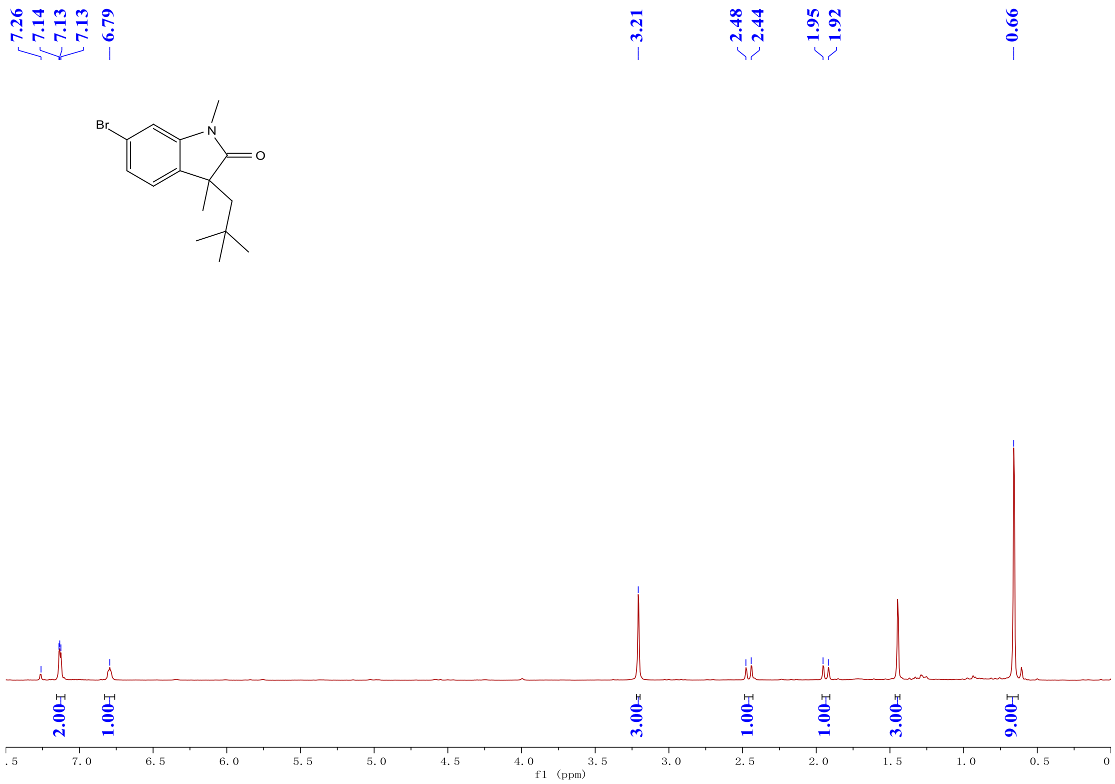

^{1}H NMR spectrum of **3sa** (400 MHz, $CDCl_3$)

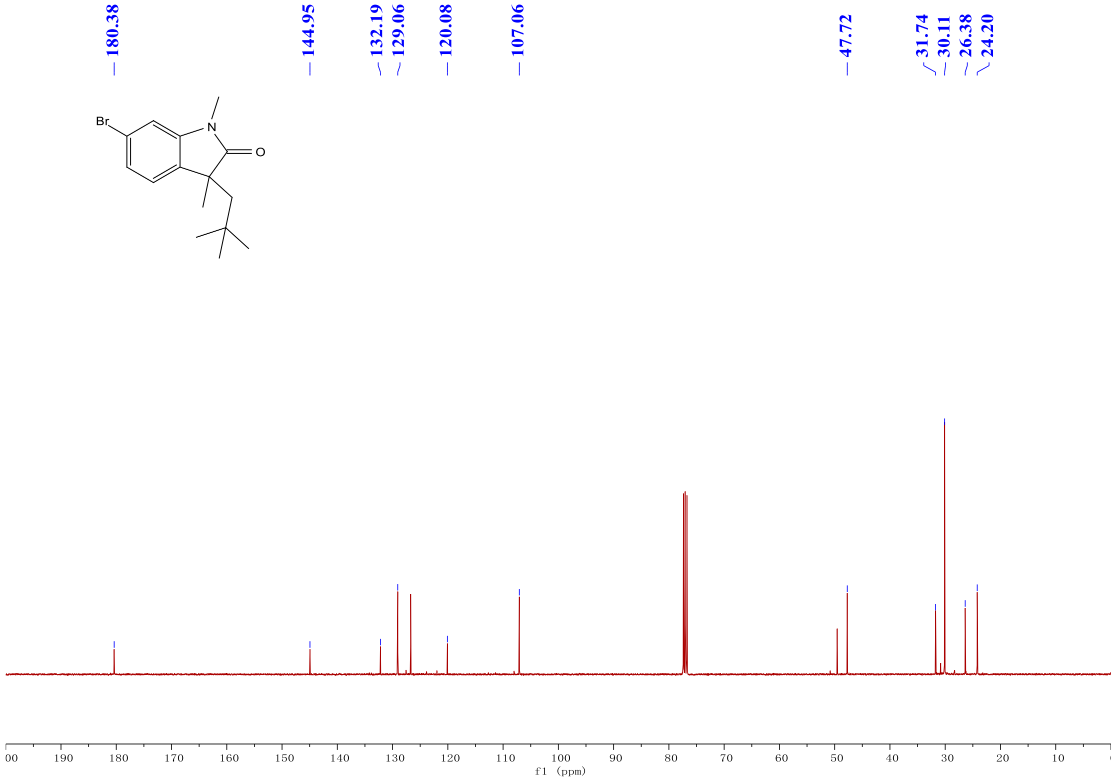

^{13}C {^{1}H} NMR spectrum of Compound **3sa** (101 MHz, $CDCl_3$)

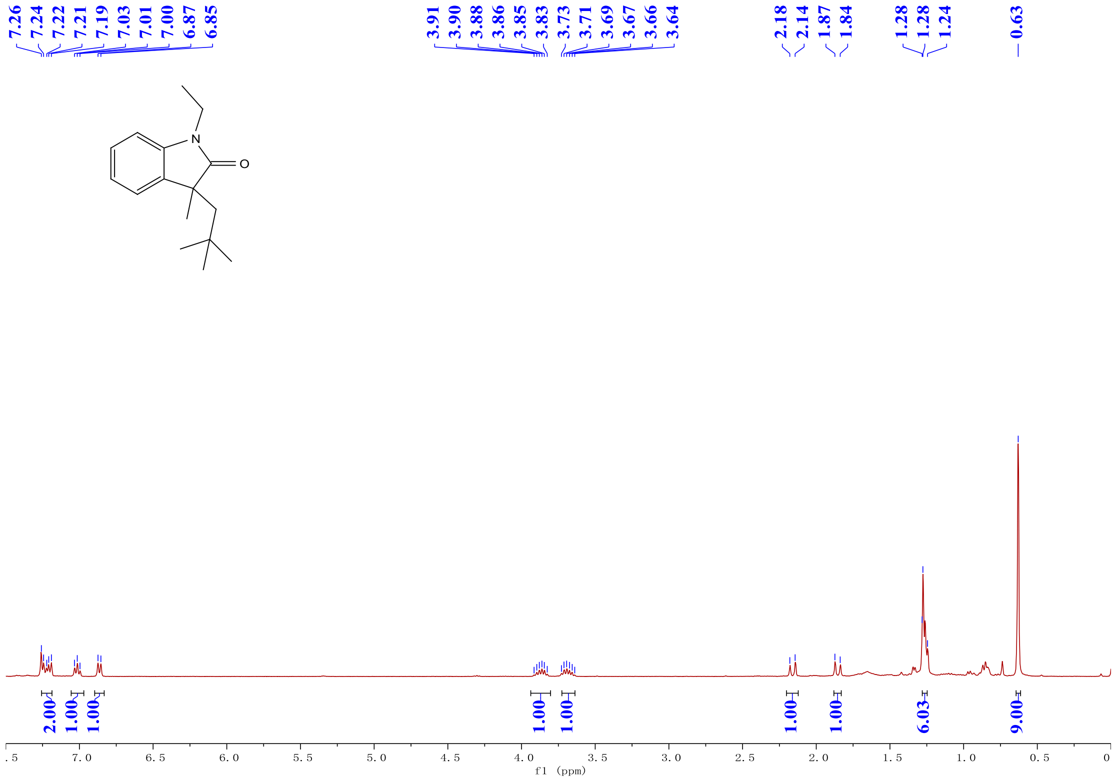

^{1}H NMR spectrum of **3ta** (400 MHz, $CDCl_3$)

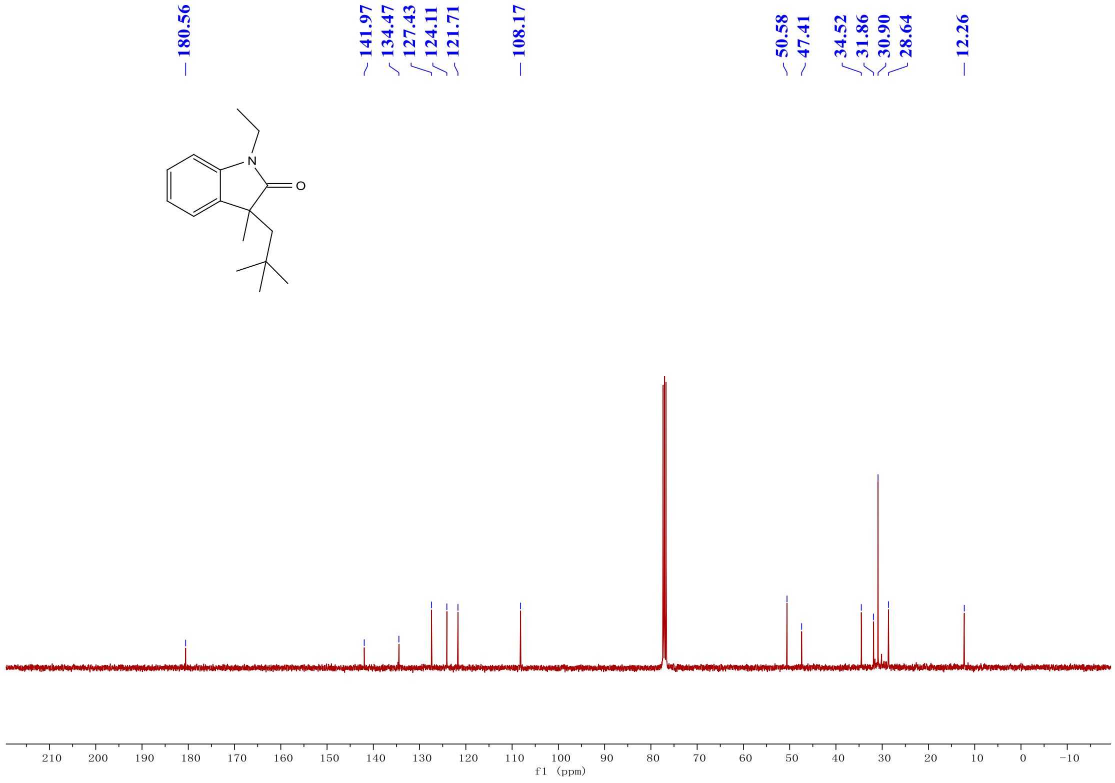

^{13}C {^{1}H} NMR spectrum of Compound **3ta** (101 MHz, $CDCl_3$)

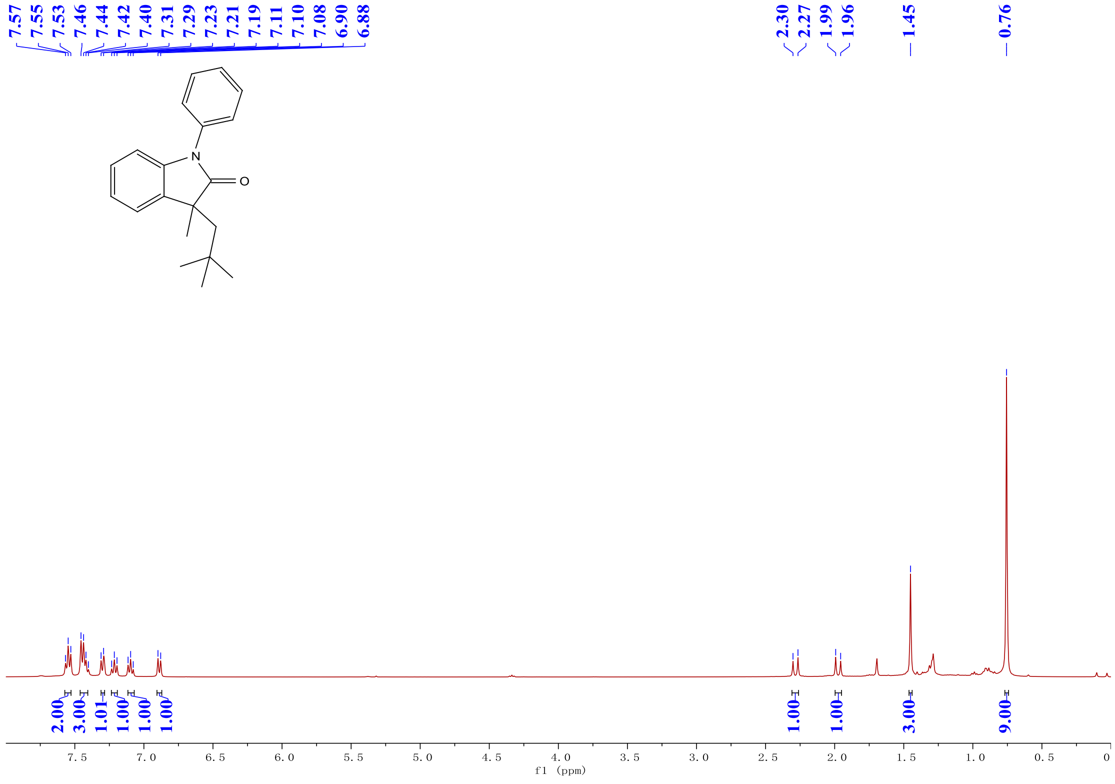

^{1}H NMR spectrum of **3ua** (400 MHz, $CDCl_3$)

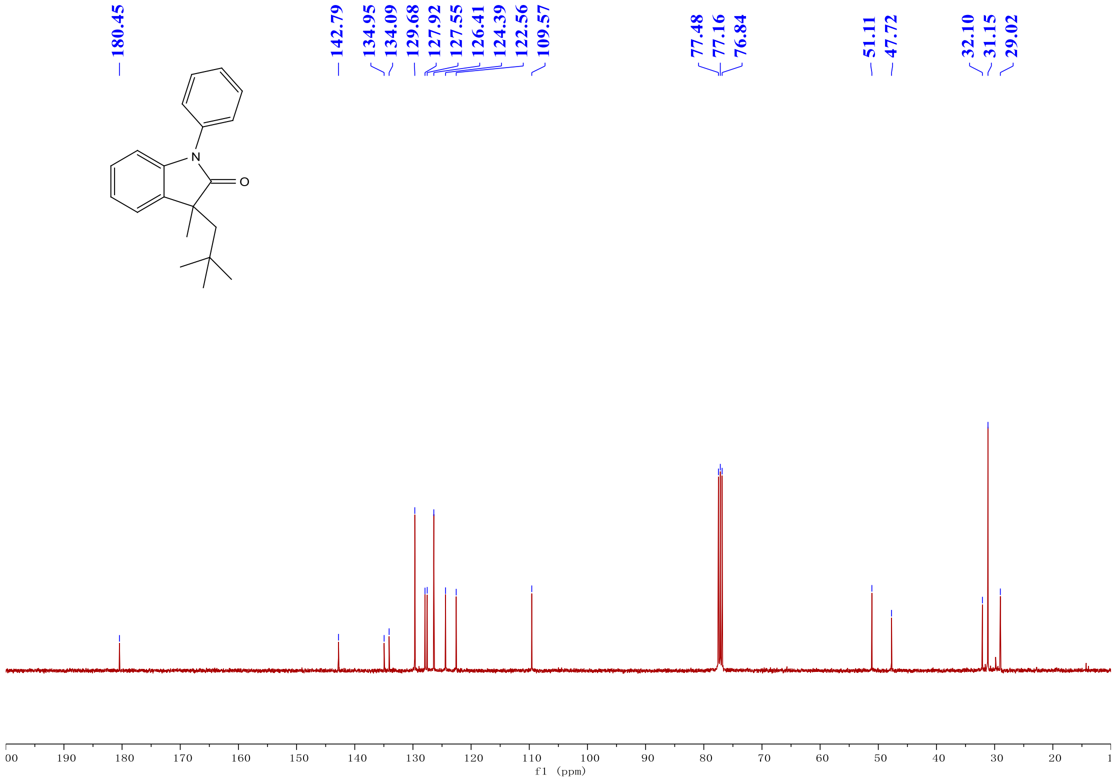

^{13}C {^{1}H} NMR spectrum of Compound **3ua** (101 MHz, $CDCl_3$)

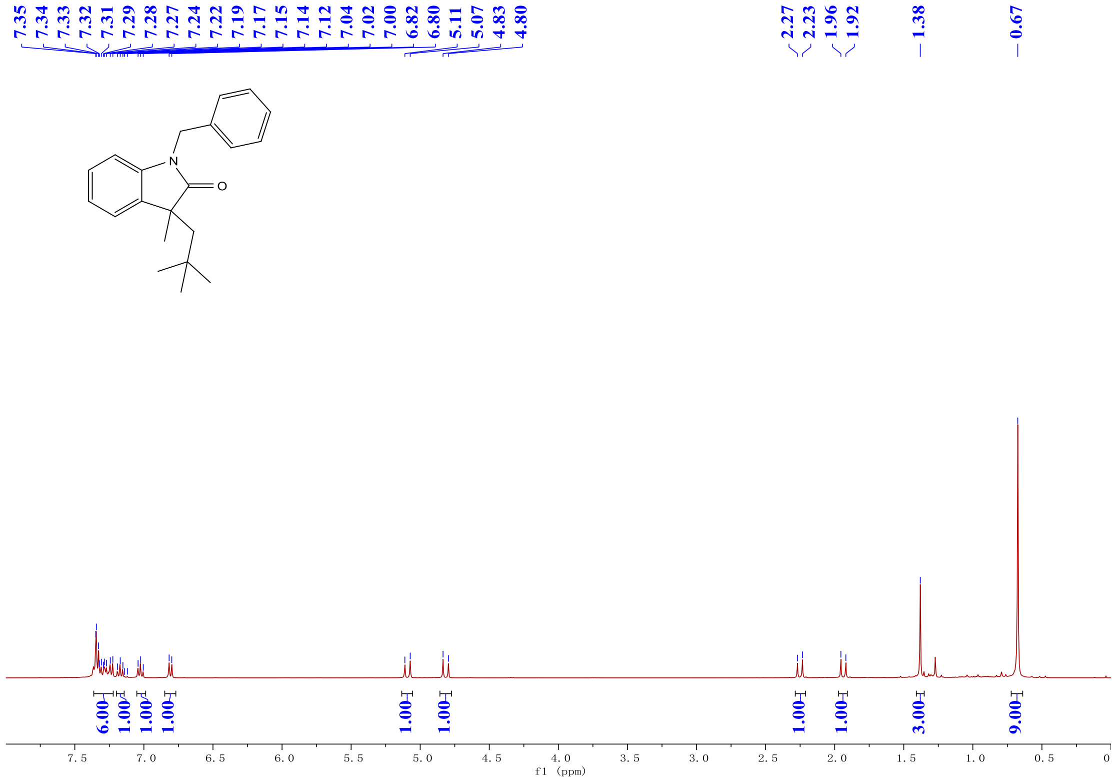

^{13}C {^{1}H} NMR spectrum of Compound **3va** (101 MHz, $CDCl_3$)

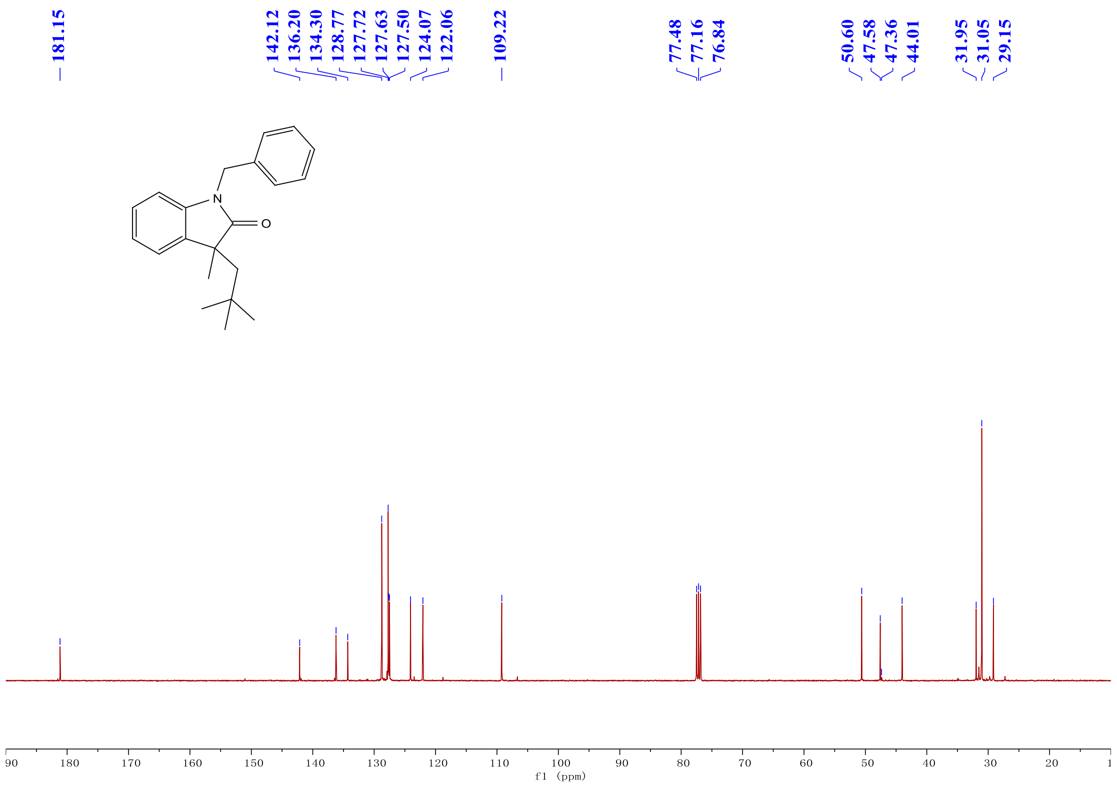

^{13}C {^{1}H} NMR spectrum of Compound **3va** (101 MHz, $CDCl_3$)

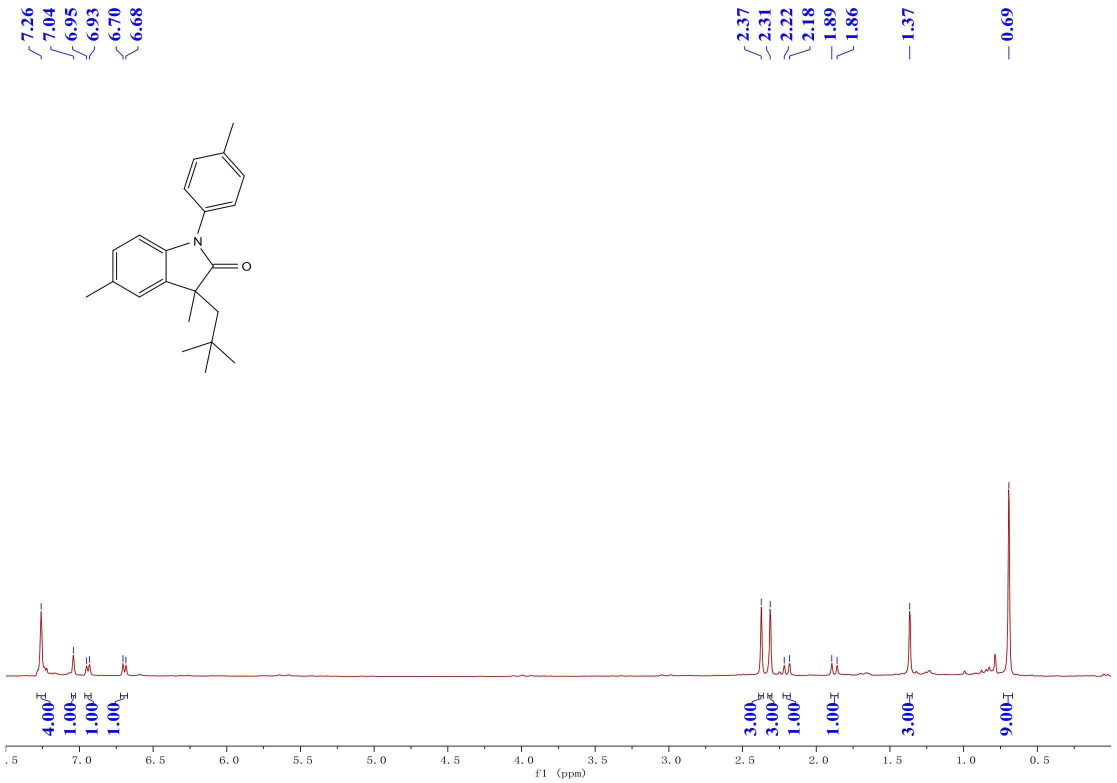

^{1}H NMR spectrum of **3xa** (400 MHz, $CDCl_3$)

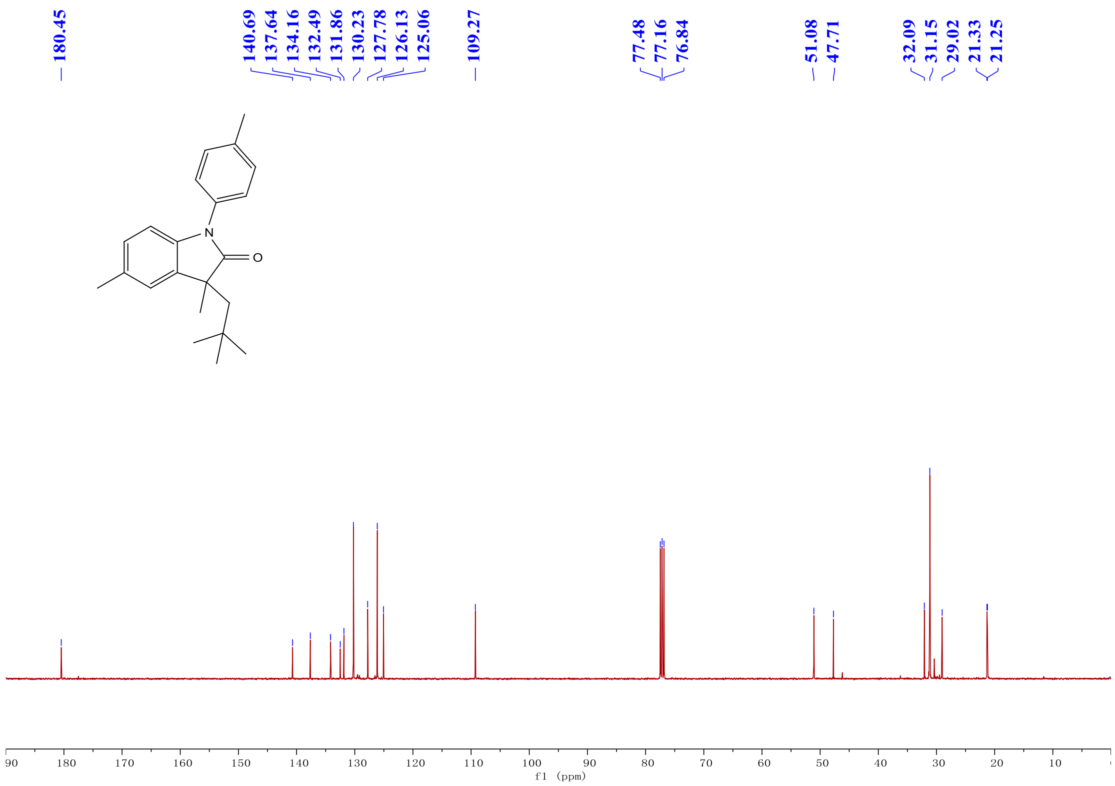

^{13}C {^{1}H} NMR spectrum of Compound **3xa** (101 MHz, $CDCl_3$)

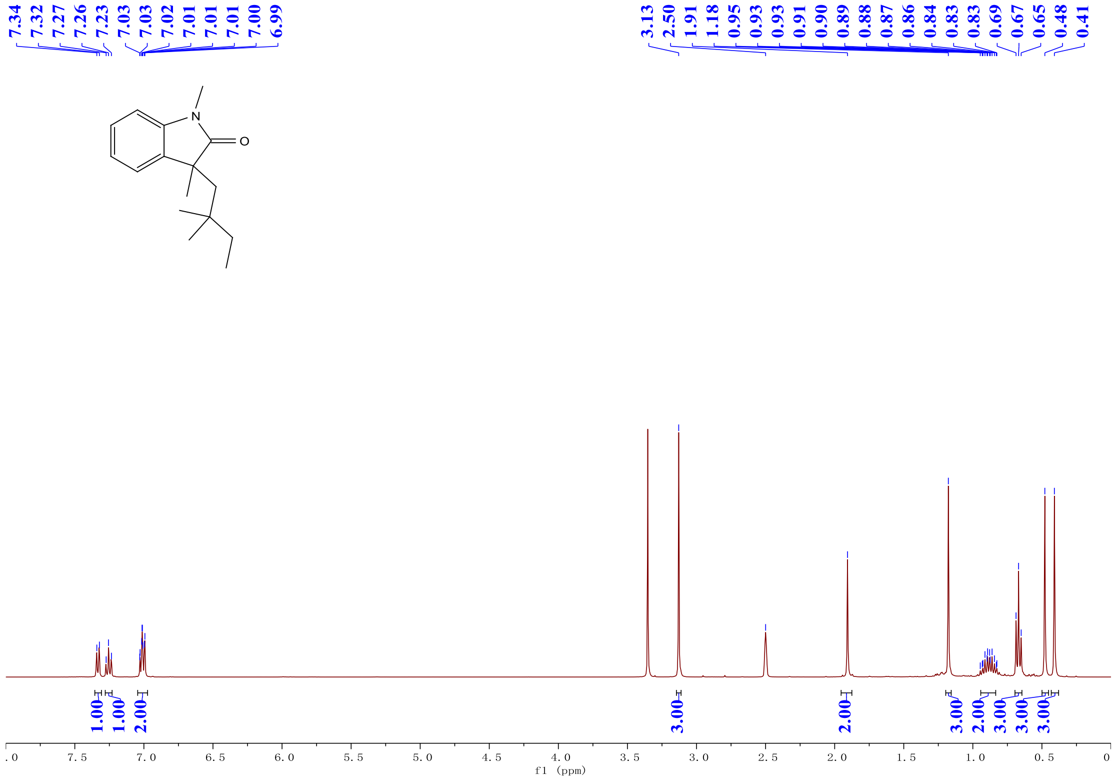

^{1}H NMR spectrum of **3ab** (400 MHz, DMSO-d_6)

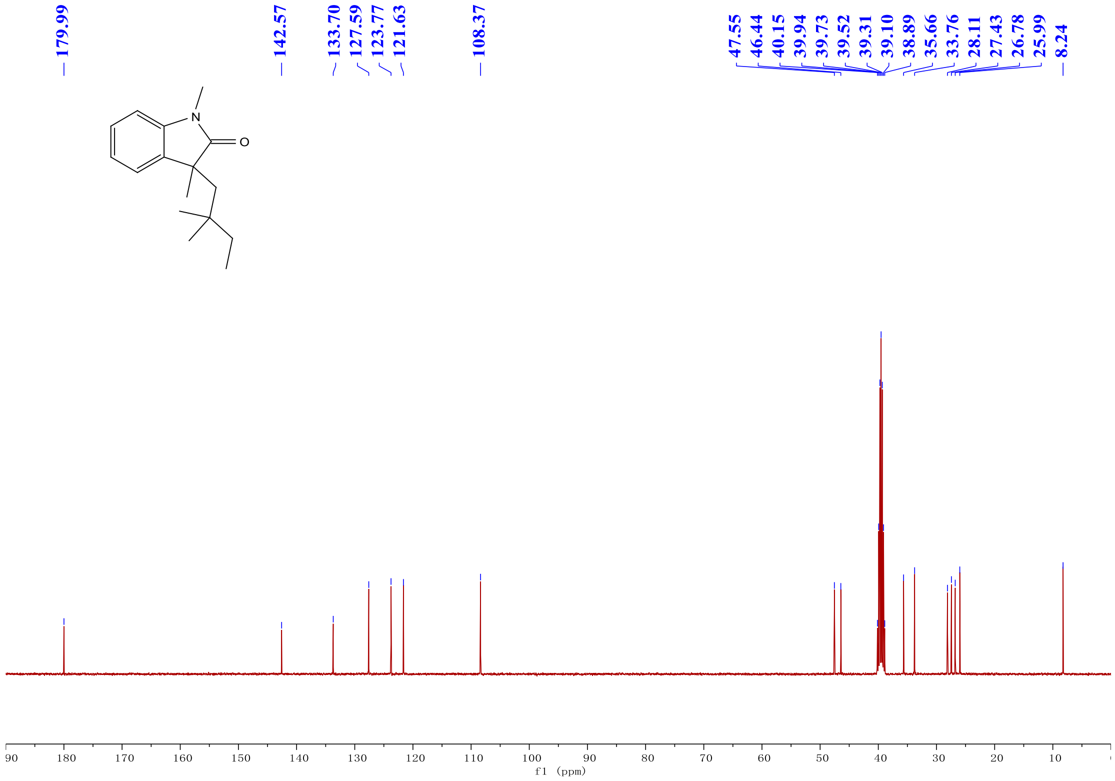

^{13}C {^{1}H} NMR spectrum of **3ab** (101 MHz, DMSO-d_6)

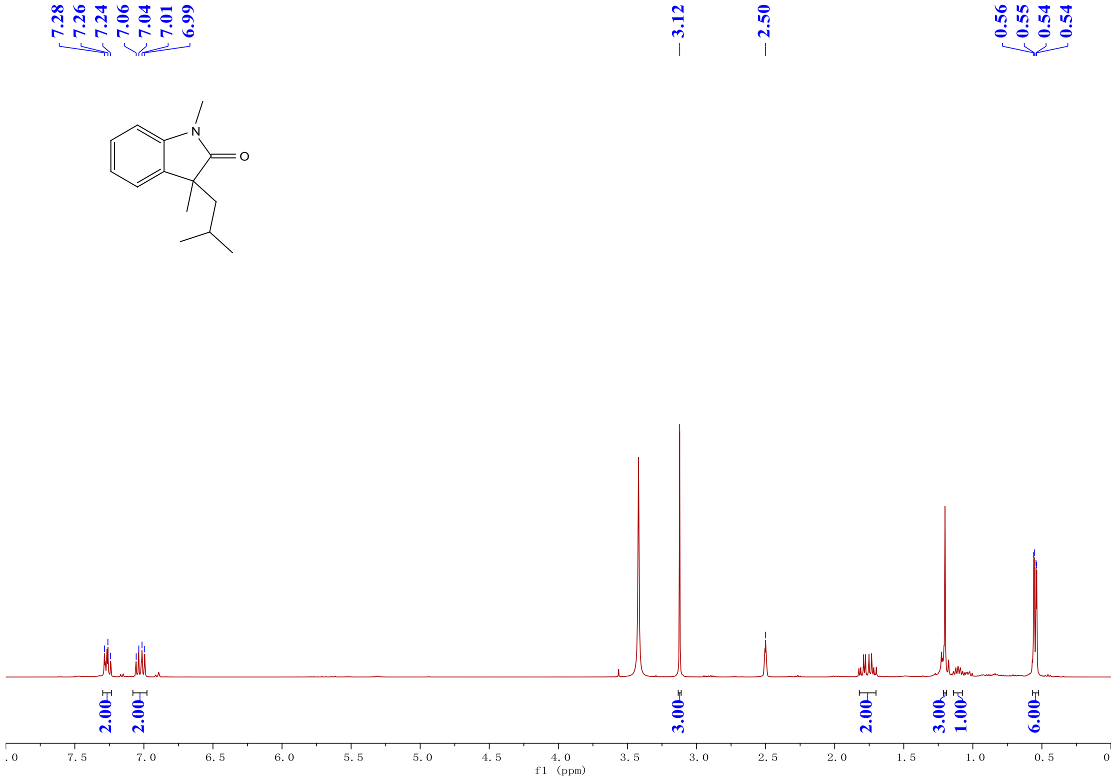

^{1}H NMR spectrum of **3ac** (400 MHz, DMSO-d_6)

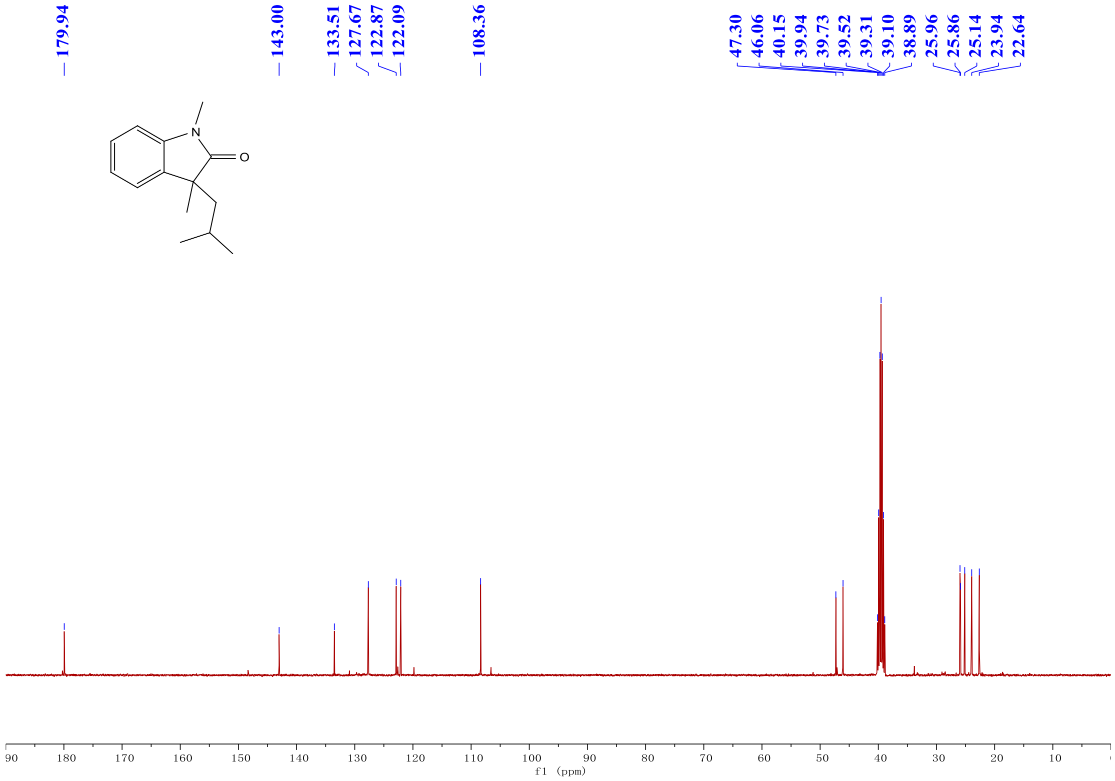

^{13}C {^{1}H} NMR spectrum of **3ac** (101 MHz, DMSO-d_6)

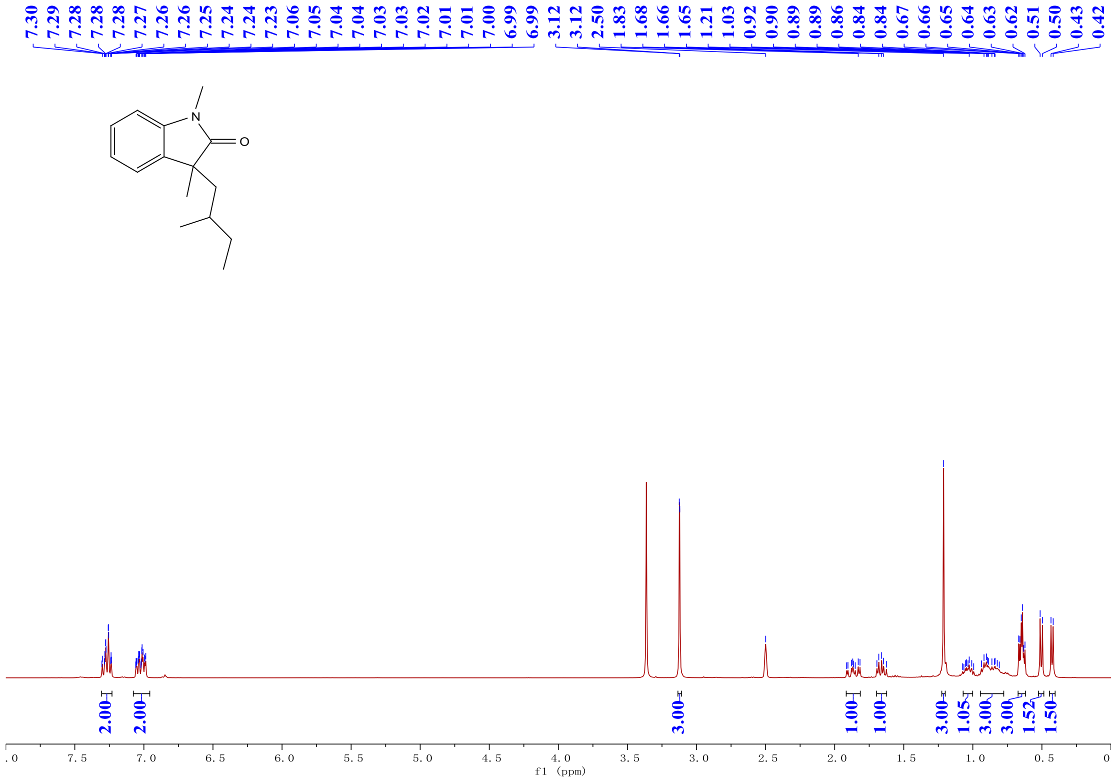

^{1}H NMR spectrum of **3ad** (400 MHz, DMSO-d_6)

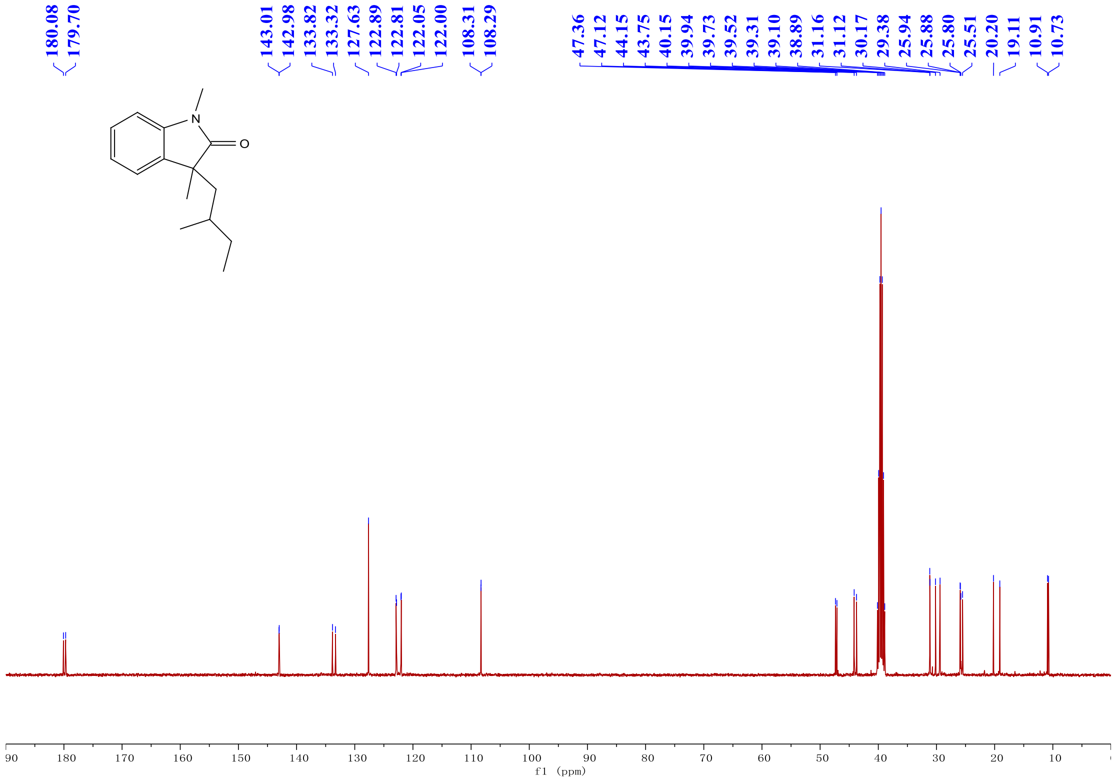

^{13}C {^{1}H} NMR spectrum of **3ad** (101 MHz, DMSO-d_6)

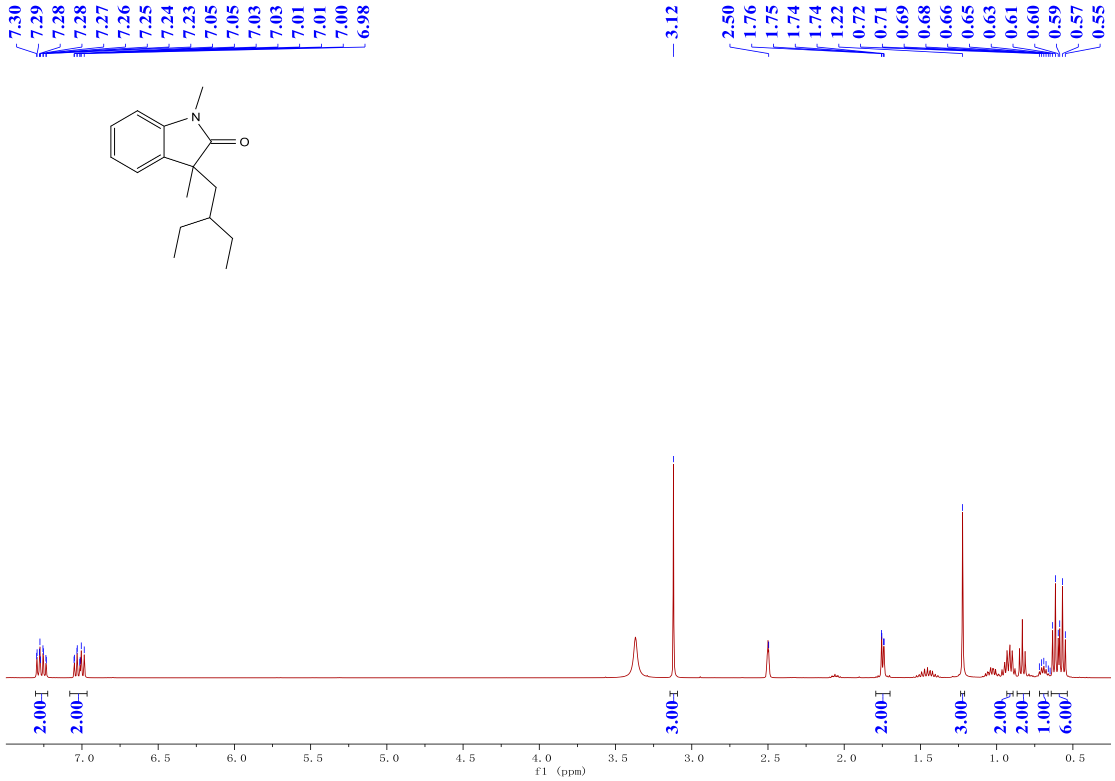

^{1}H NMR spectrum of **3ae** (400 MHz, DMSO-d_6)

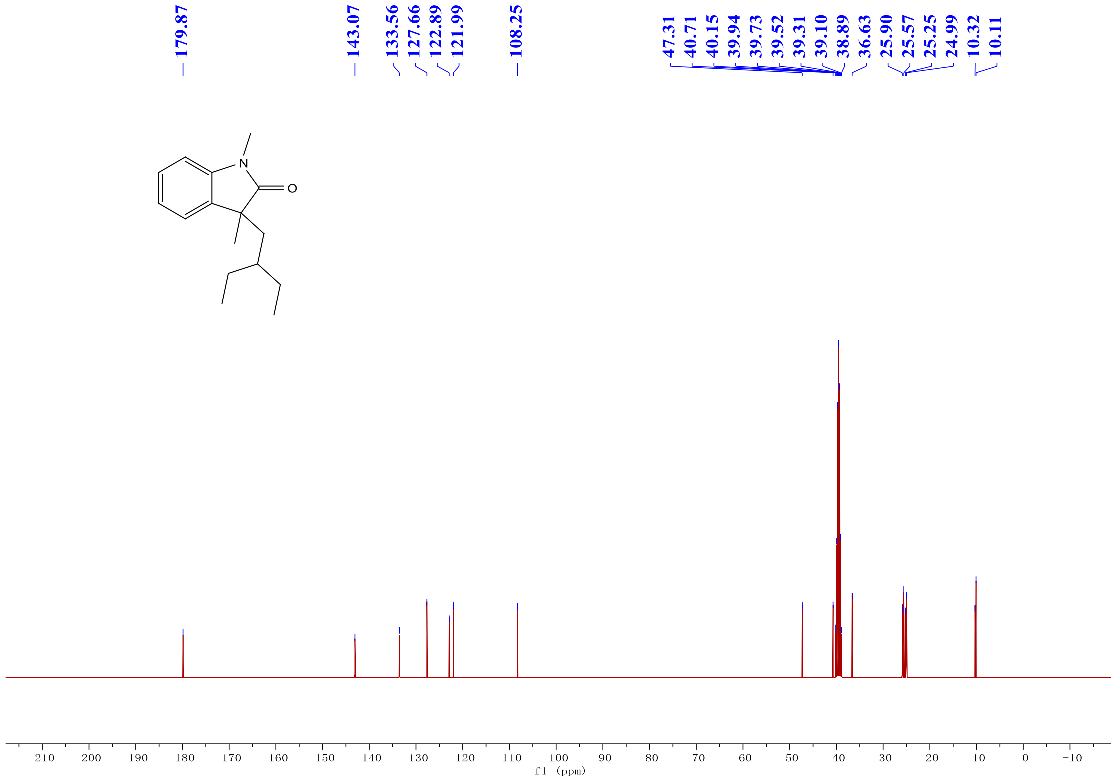

^{13}C {^{1}H} NMR spectrum of **3ae** (101 MHz, DMSO-d_6)

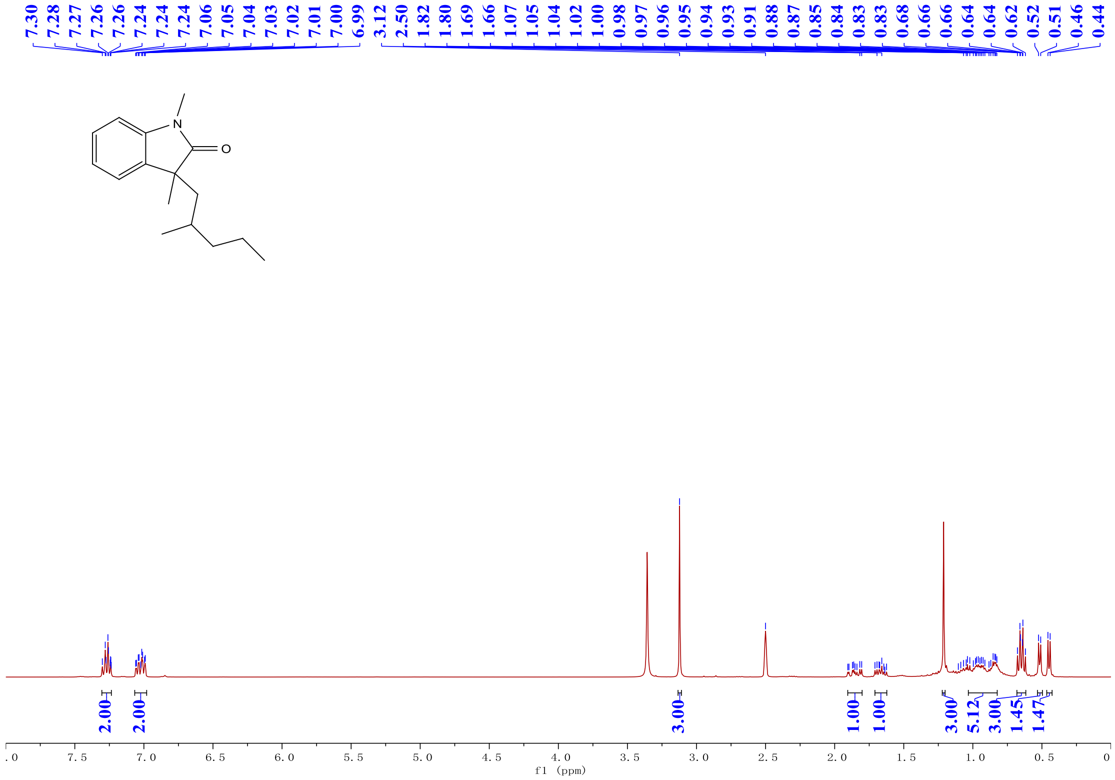

^{1}H NMR spectrum of **3af** (400 MHz, DMSO-d_6)

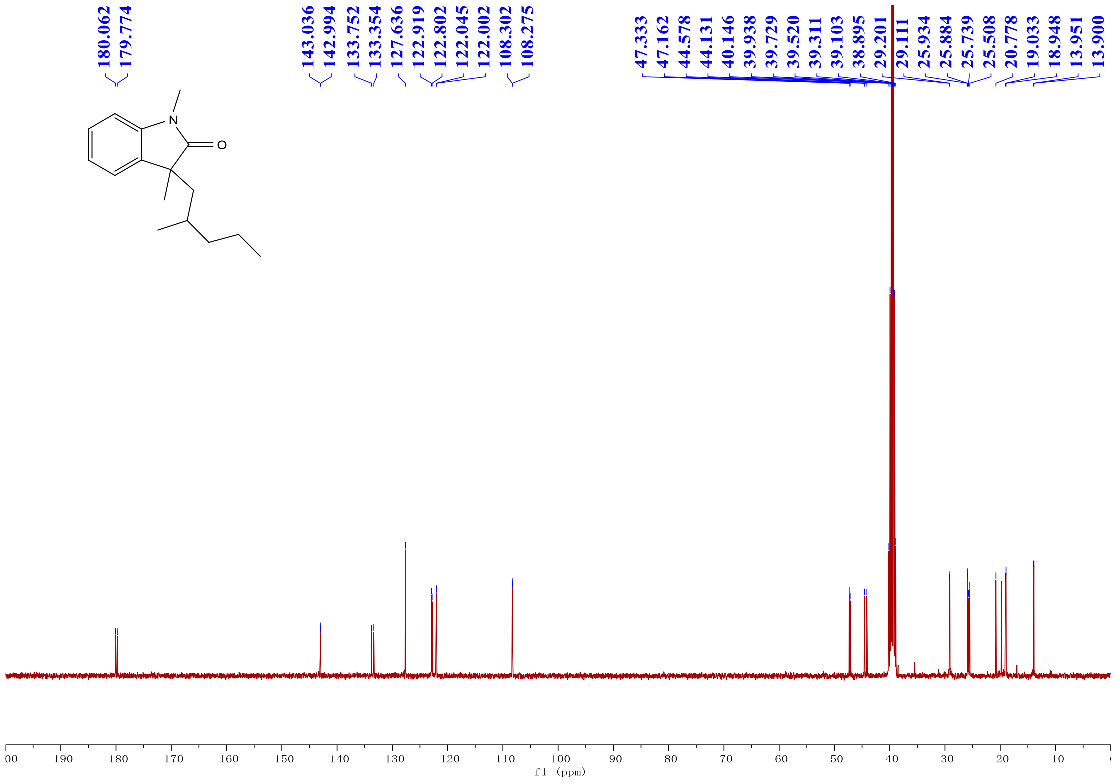

^{13}C {^{1}H} NMR spectrum of **3af** (101 MHz, DMSO-d_6)

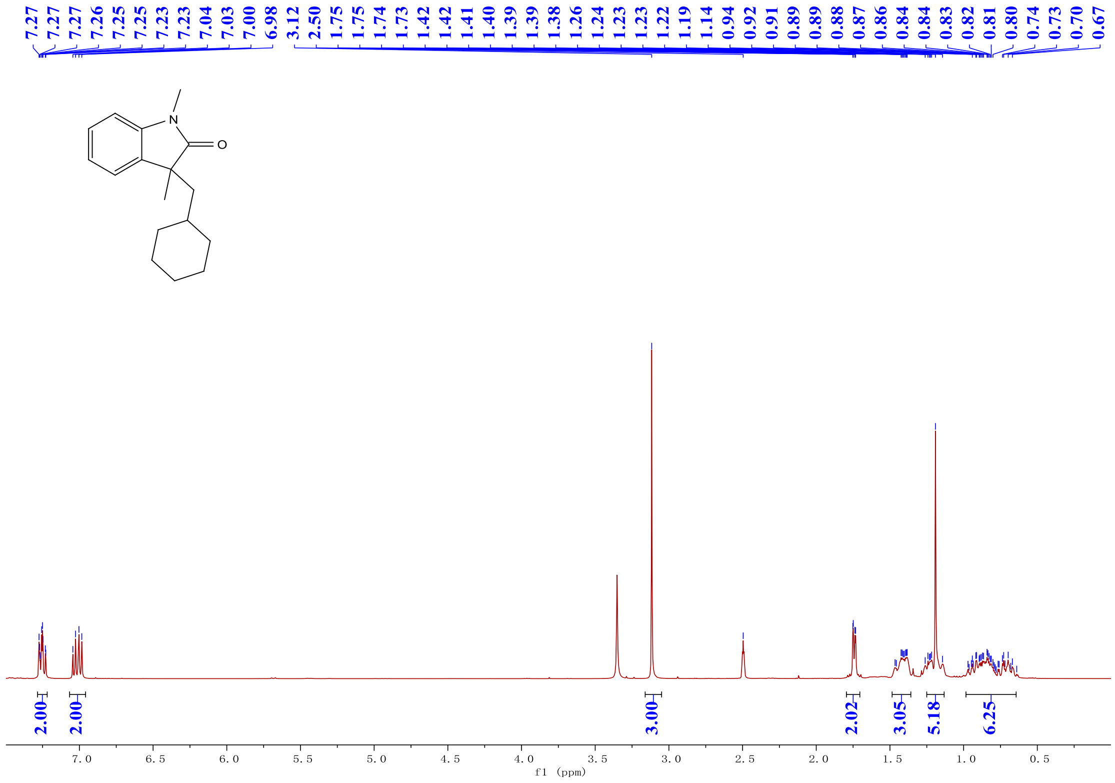

^{1}H NMR spectrum of **3ag** (400 MHz, DMSO-d_6)

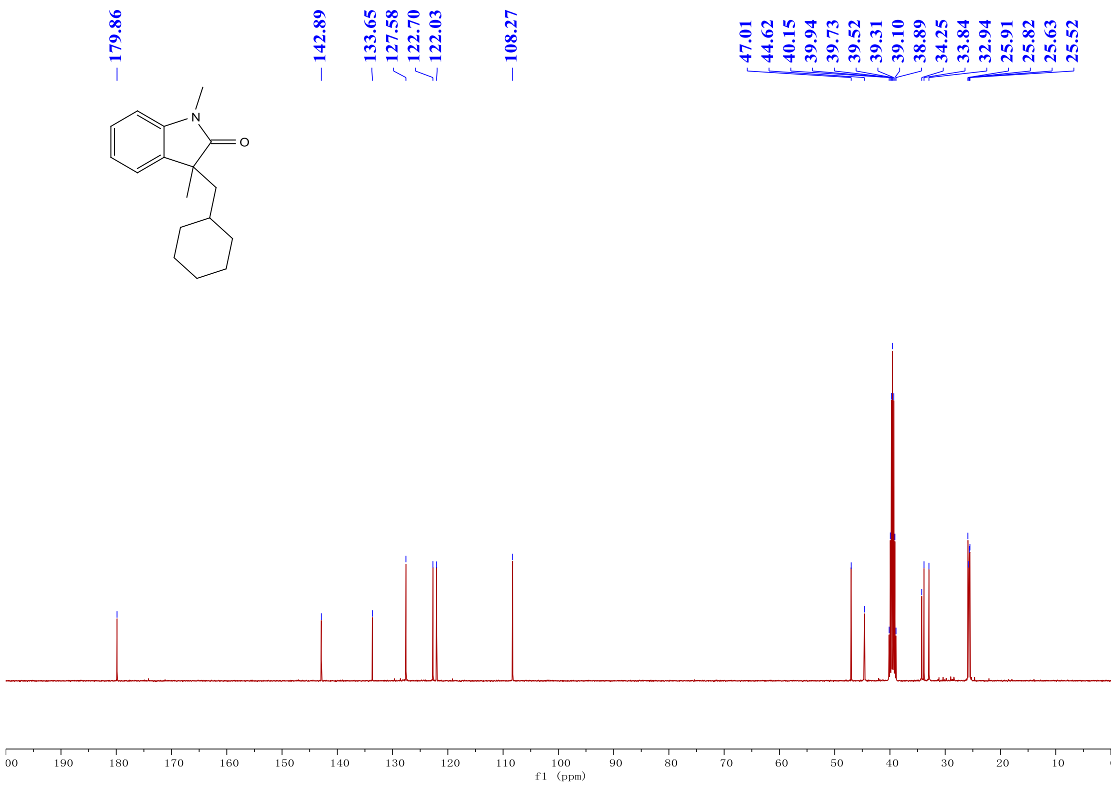

^{13}C {^{1}H} NMR spectrum of **3ag** (101 MHz, DMSO-d_6)

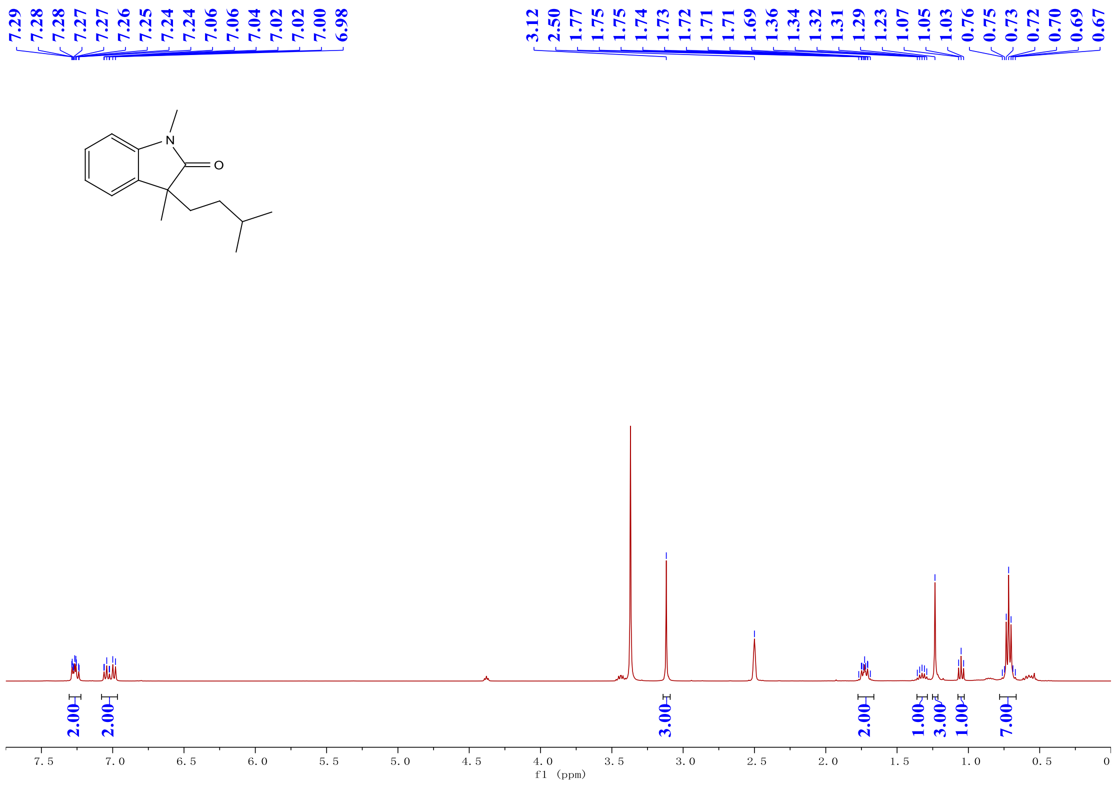

^{1}H NMR spectrum of **3ah** (400 MHz, DMSO-d_6)

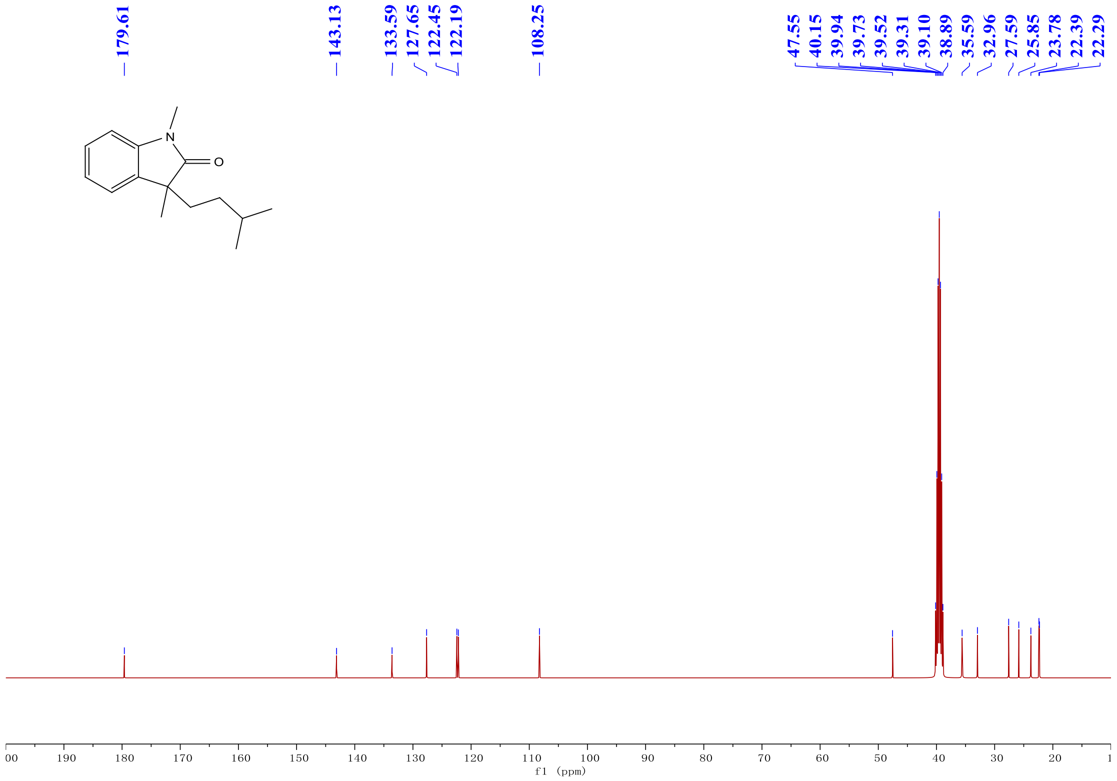

^{13}C {^{1}H} NMR spectrum of **3ah** (101 MHz, DMSO-d_6)

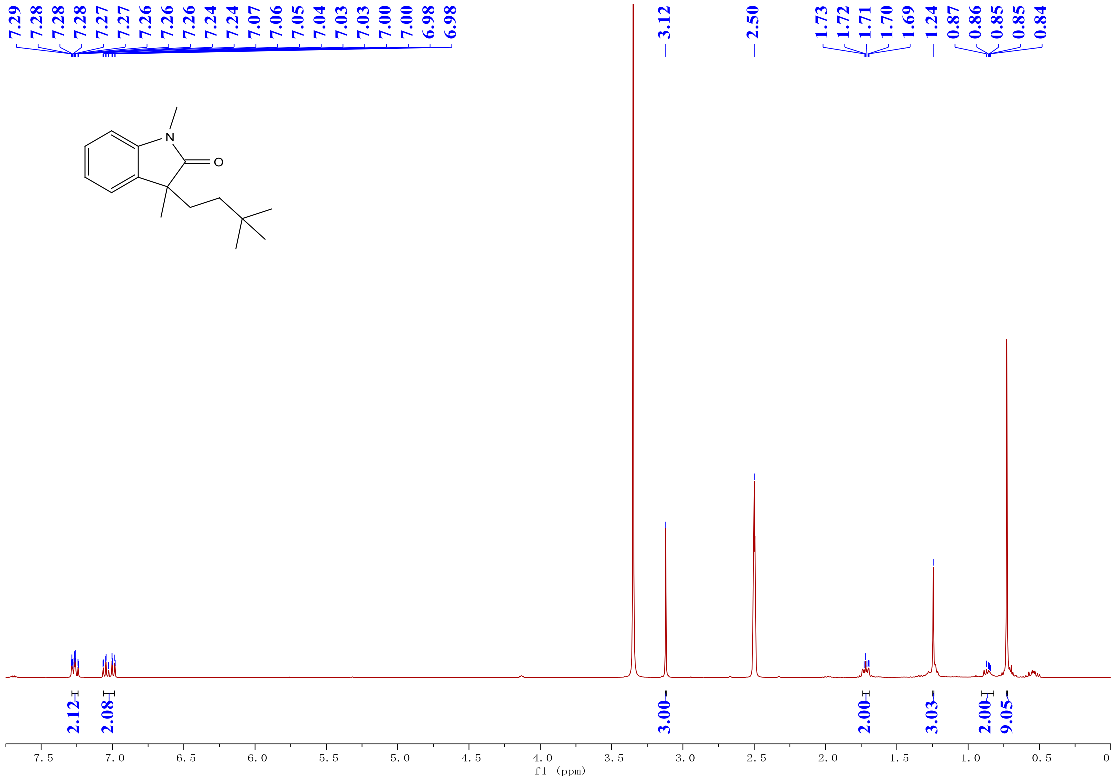

^{1}H NMR spectrum of **3ai** (400 MHz, DMSO-d_6)

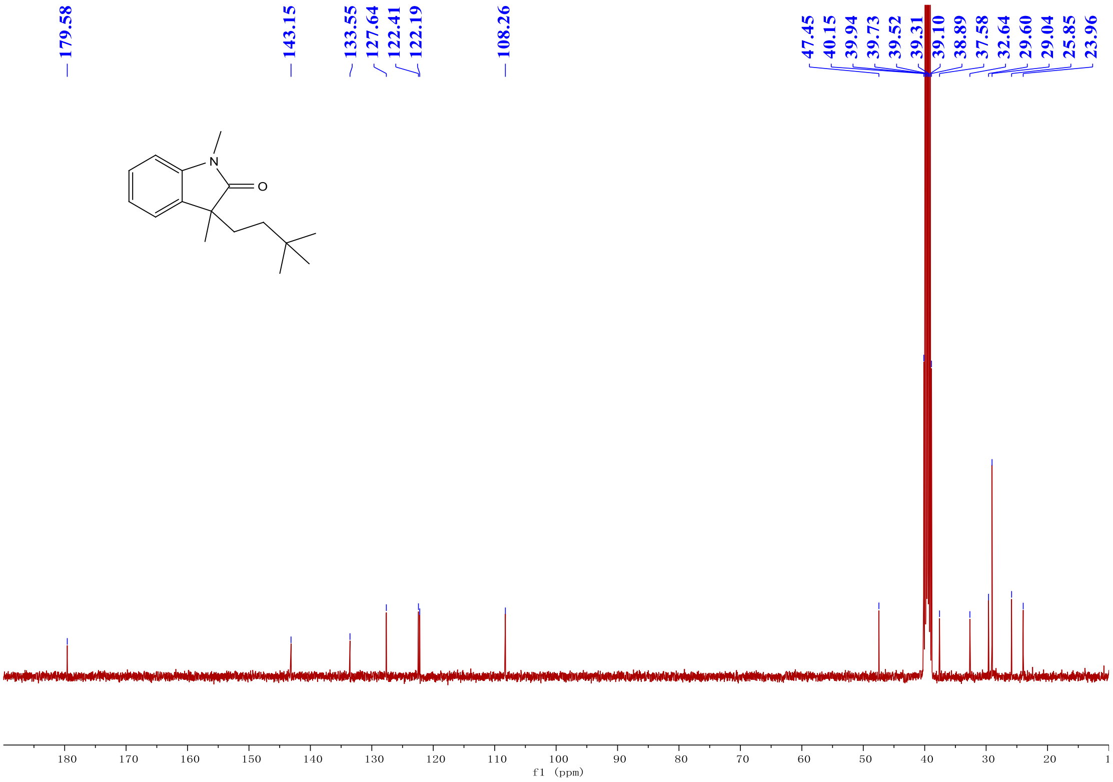

^{13}C {^{1}H} NMR spectrum of **3ai** (101 MHz, DMSO-d_6)

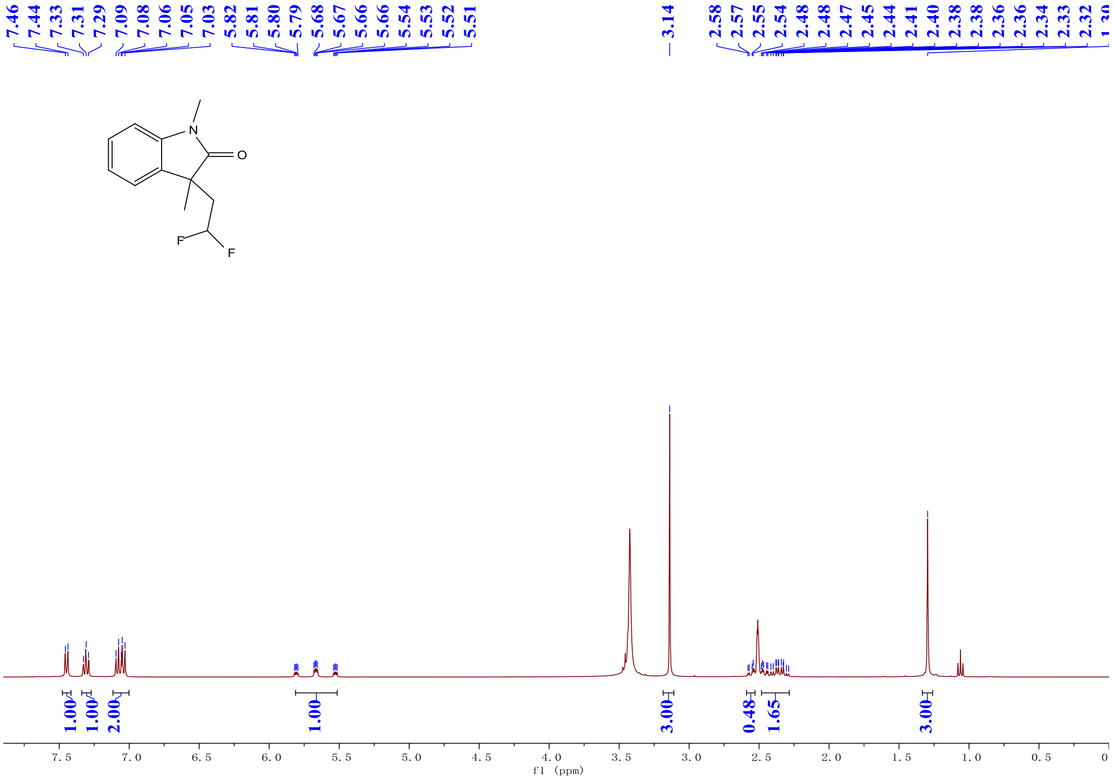

^{1}H NMR spectrum of **3aj** (400 MHz, DMSO-d_6)

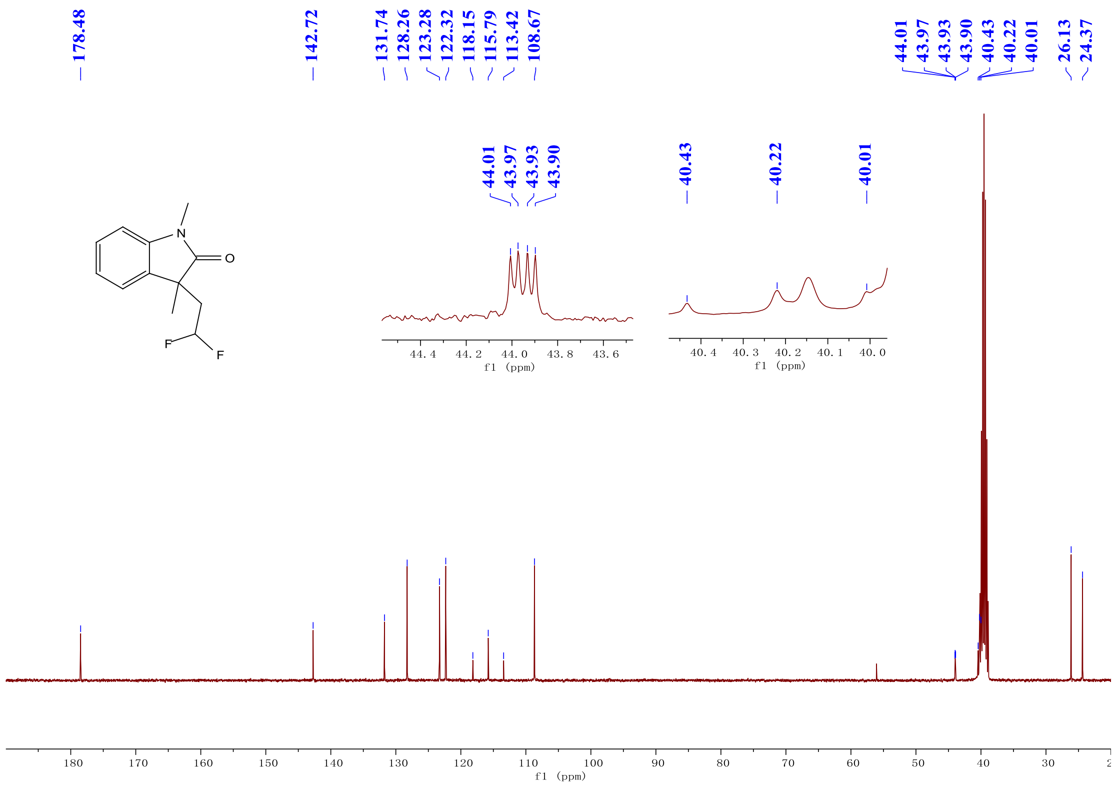

^{13}C {^{1}H} NMR spectrum of **3aj** (101 MHz, DMSO-d_6)

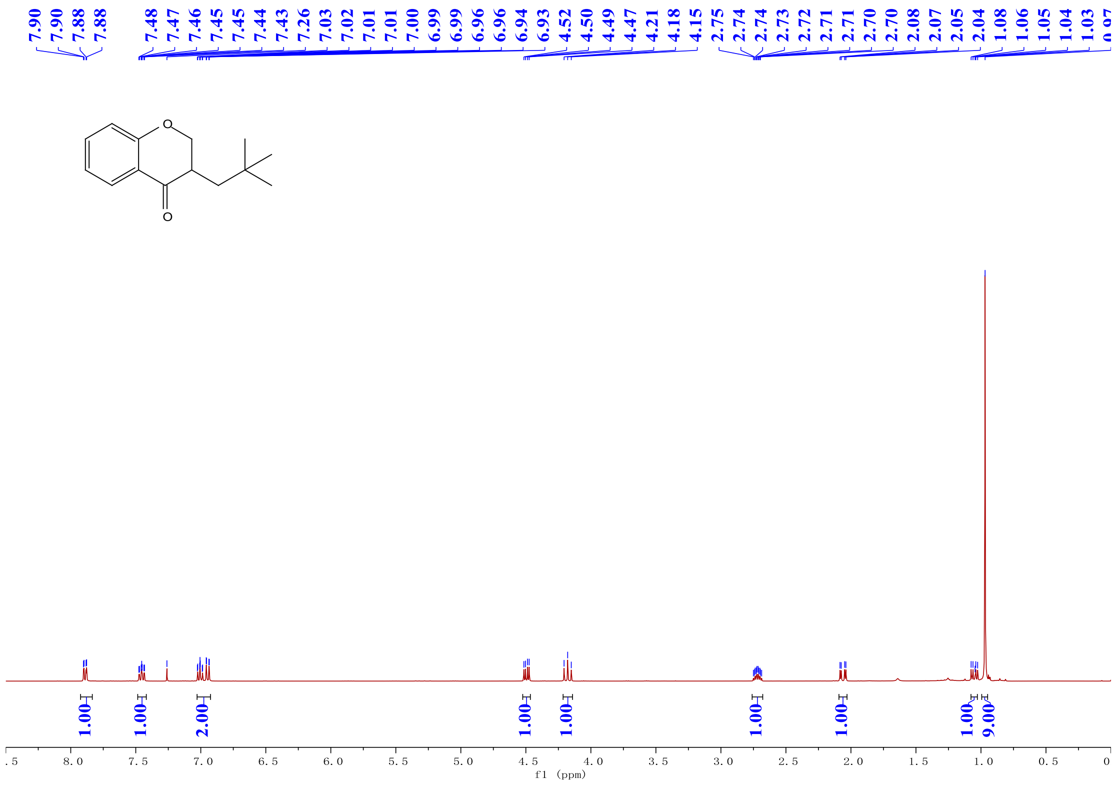

^{1}H NMR spectrum of **6aa** (400 MHz, $CDCl_3$)

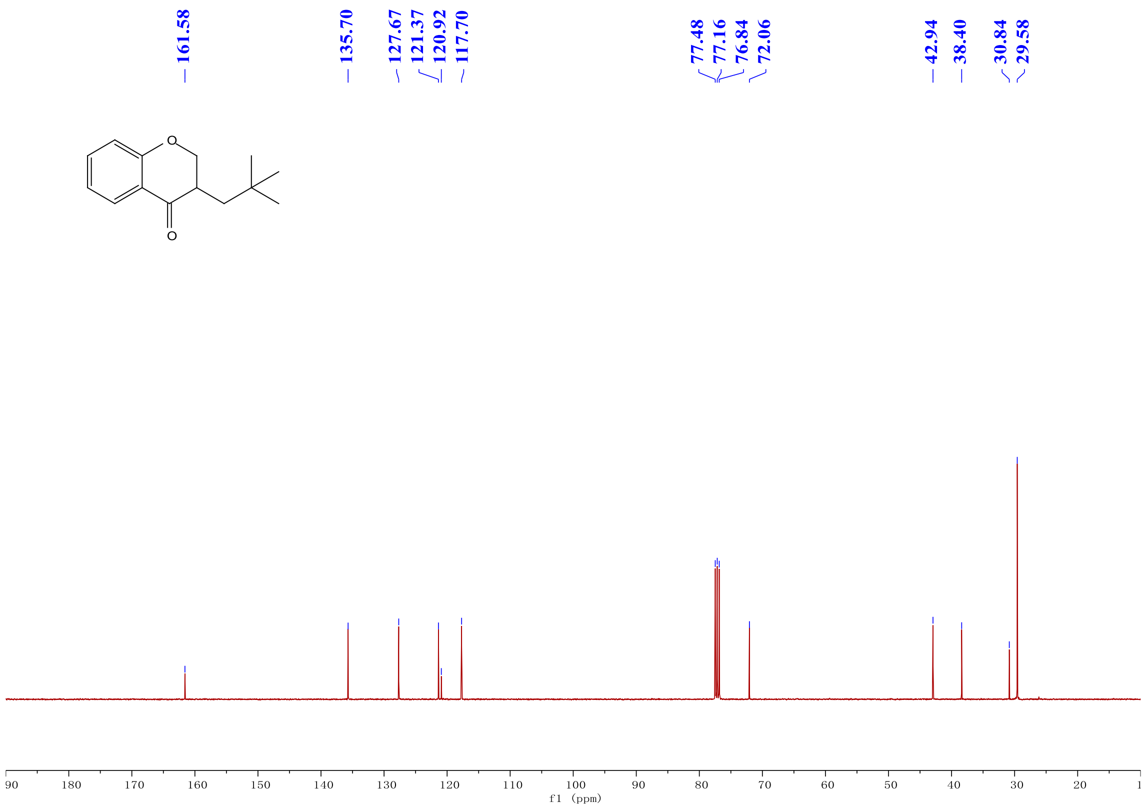

^{13}C {^{1}H} NMR spectrum of Compound **6aa** (101 MHz, $CDCl_3$)

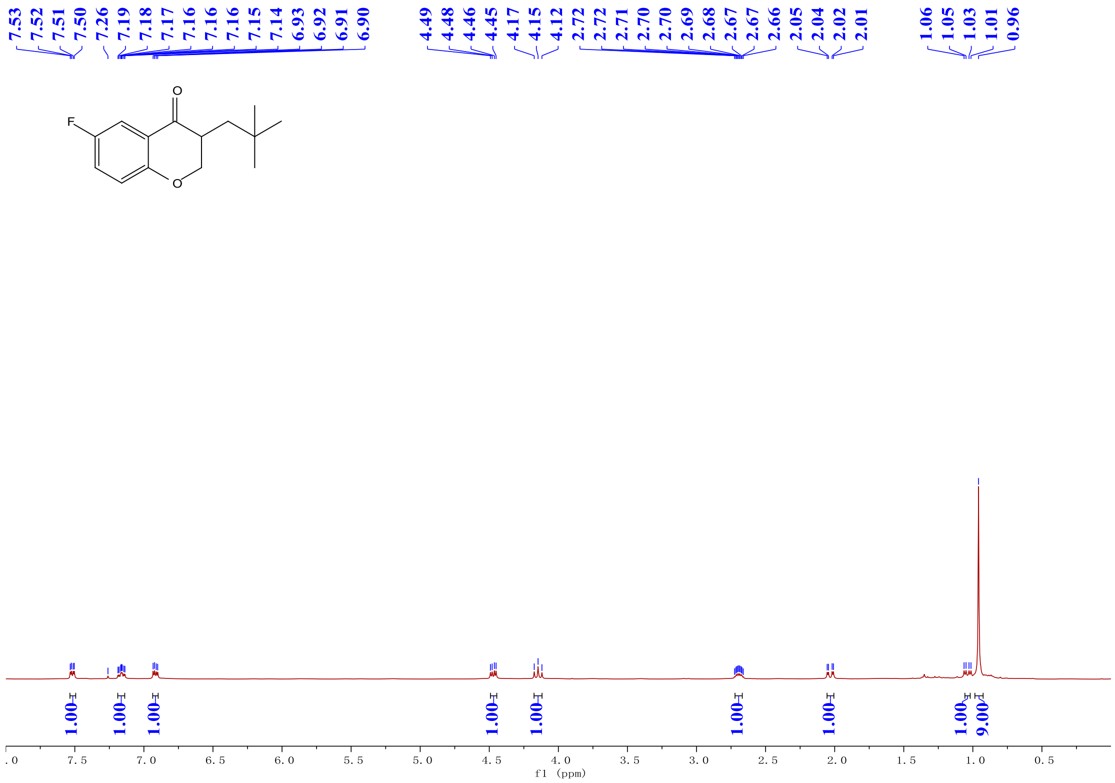

^{1}H NMR spectrum of **6ba** (400 MHz, $CDCl_3$)

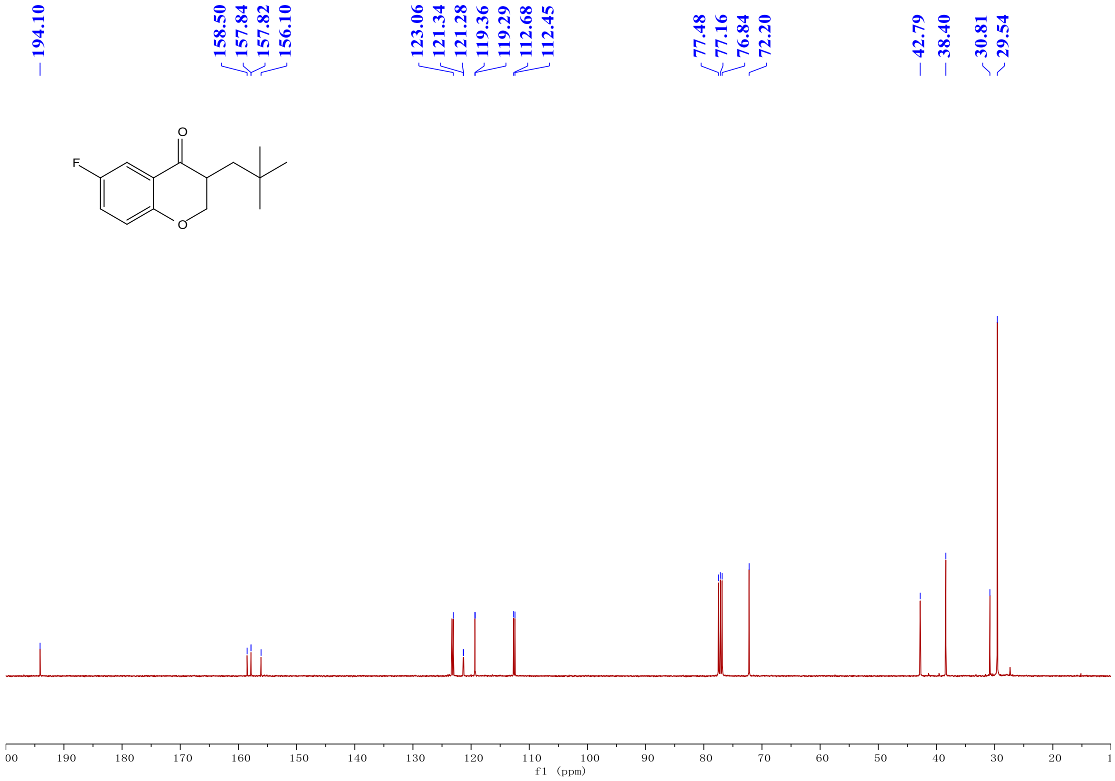

^{13}C {^{1}H} NMR spectrum of Compound **6ba** (101 MHz, $CDCl_3$)

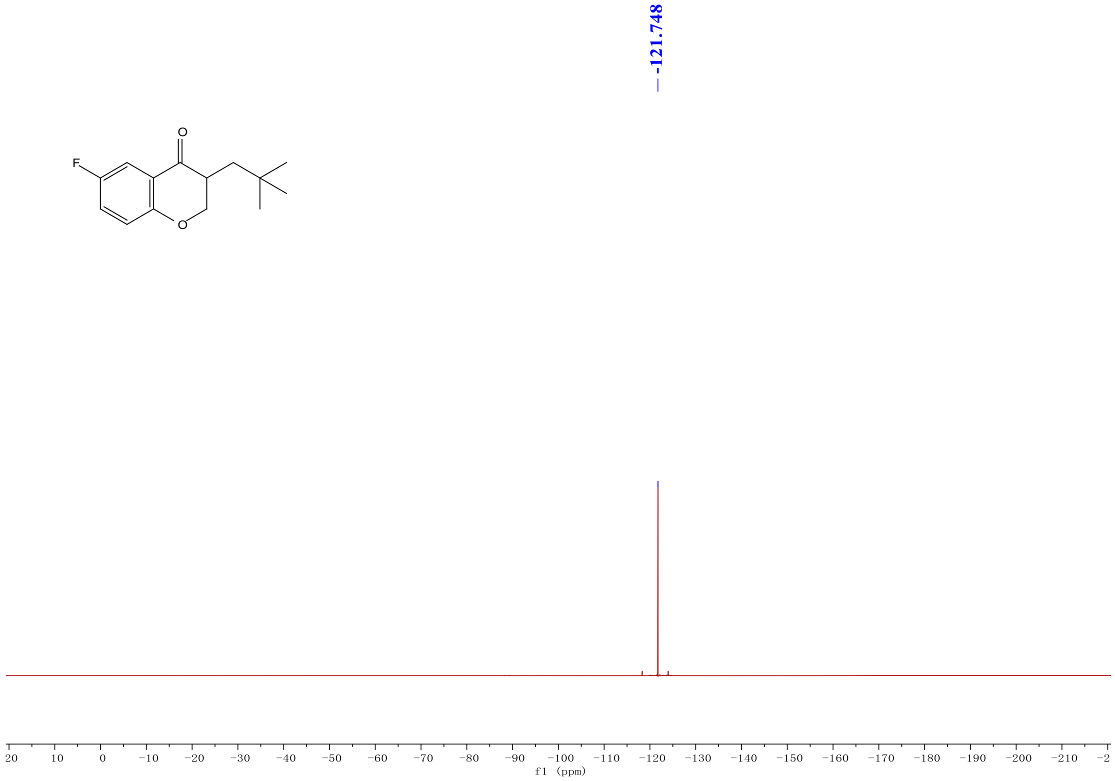

^{13}C {^{1}H} NMR spectrum of Compound **6ba** (101 MHz, $CDCl_3$)

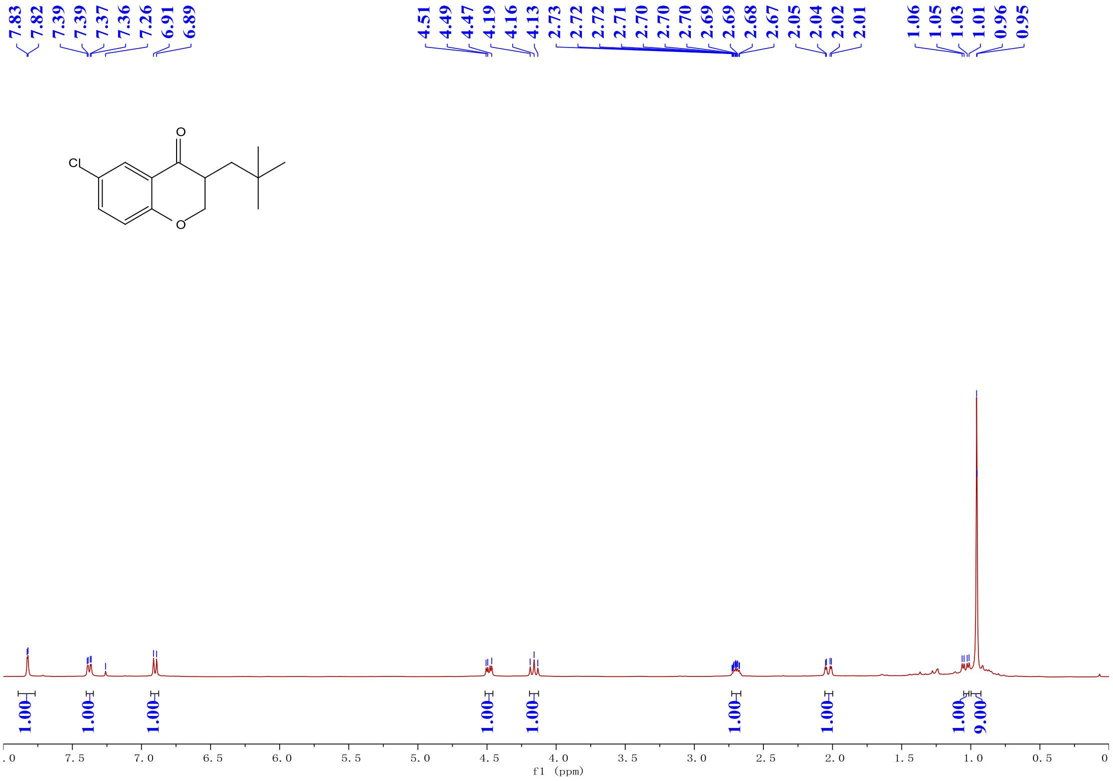

^1H NMR spectrum of **6ca** (400 MHz, $CDCl_3$)

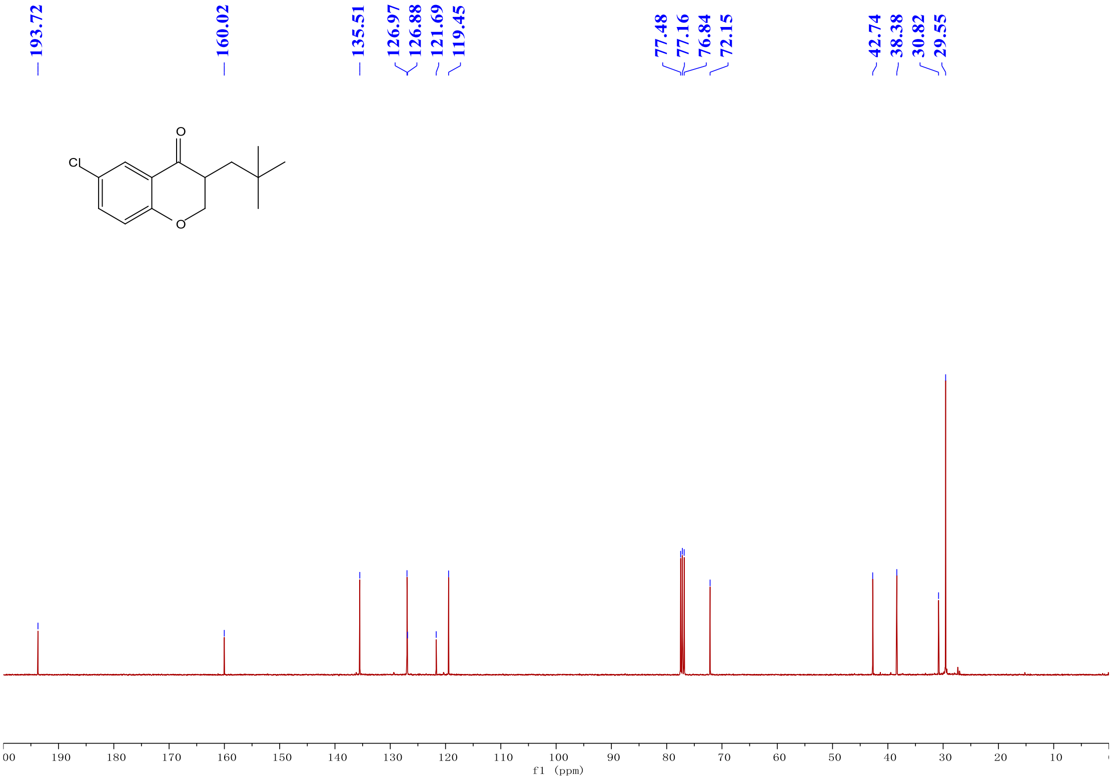

^{13}C {^1H} NMR spectrum of Compound **6ca** (101 MHz, $CDCl_3$)

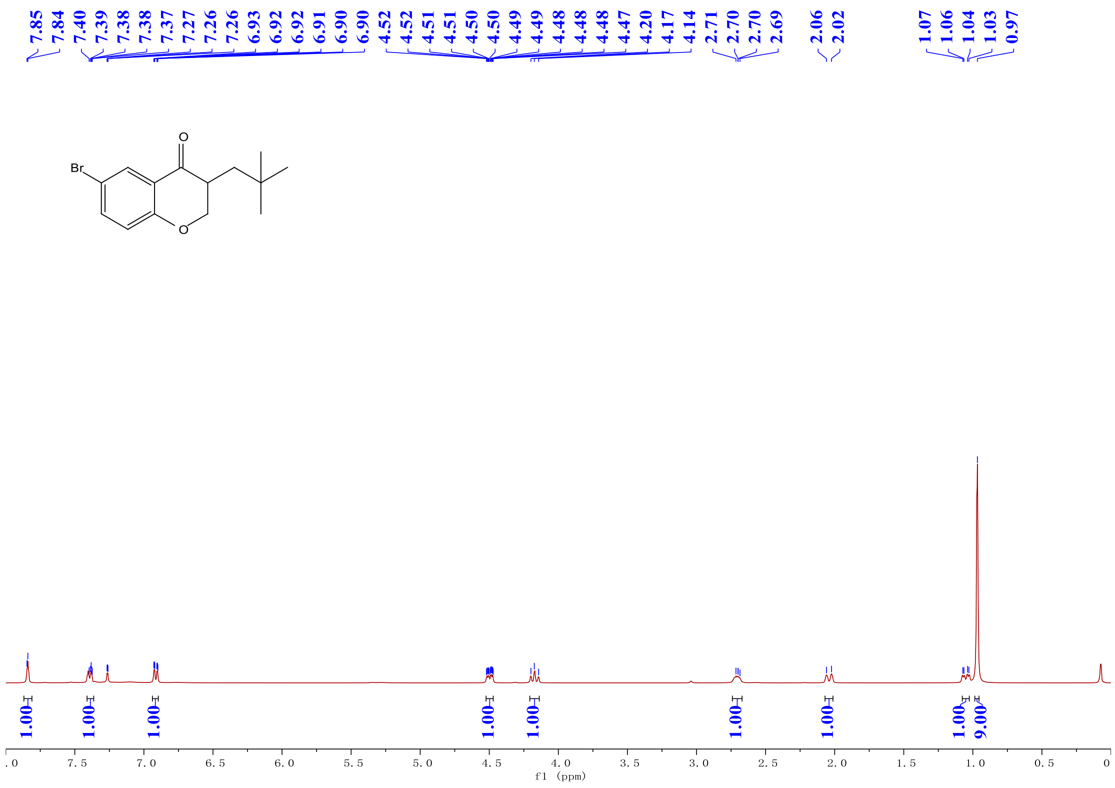

^{1}H NMR spectrum of **6da** (400 MHz, $CDCl_3$)

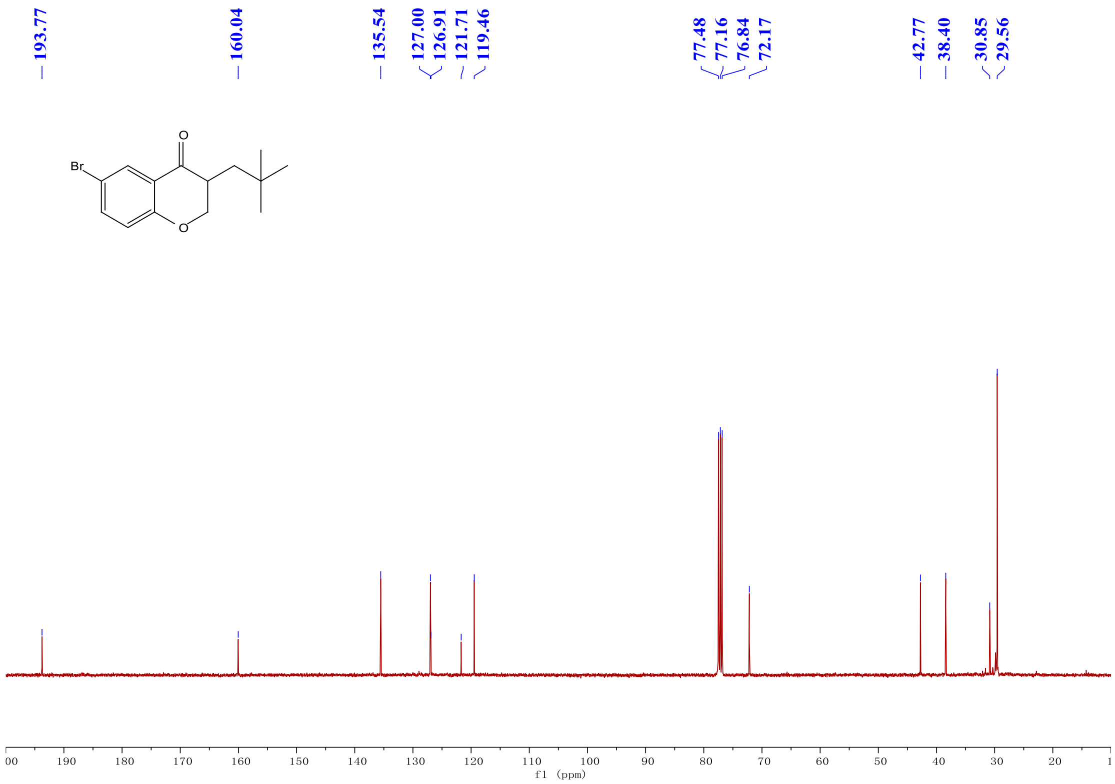

^{13}C {^{1}H} NMR spectrum of Compound **6da** (101 MHz, $CDCl_3$)

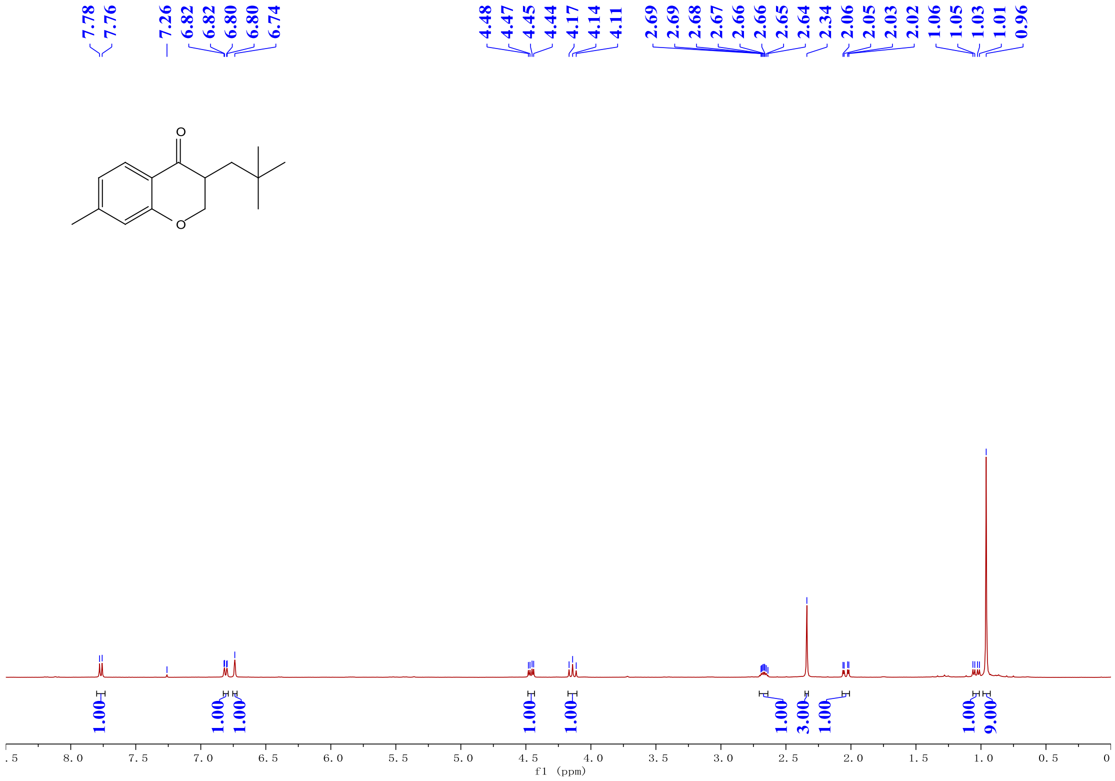

^{1}H NMR spectrum of **6ea** (400 MHz, $CDCl_3$)

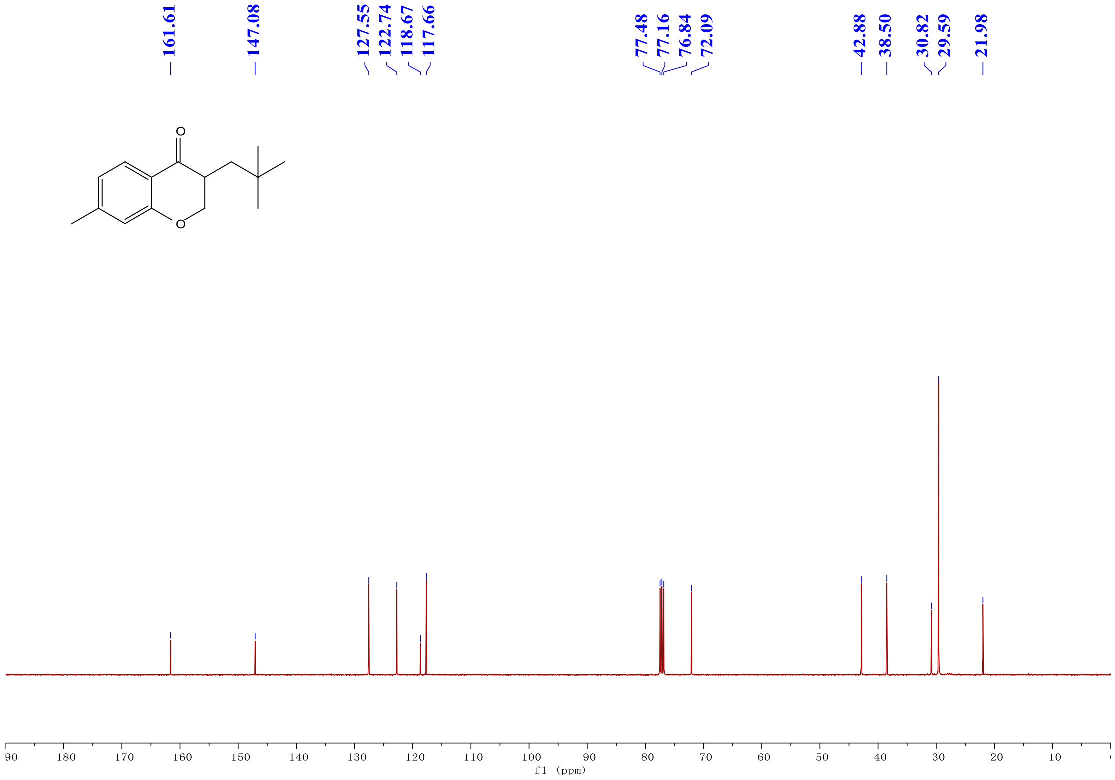

^{13}C {^{1}H} NMR spectrum of Compound **6ea** (101 MHz, $CDCl_3$)

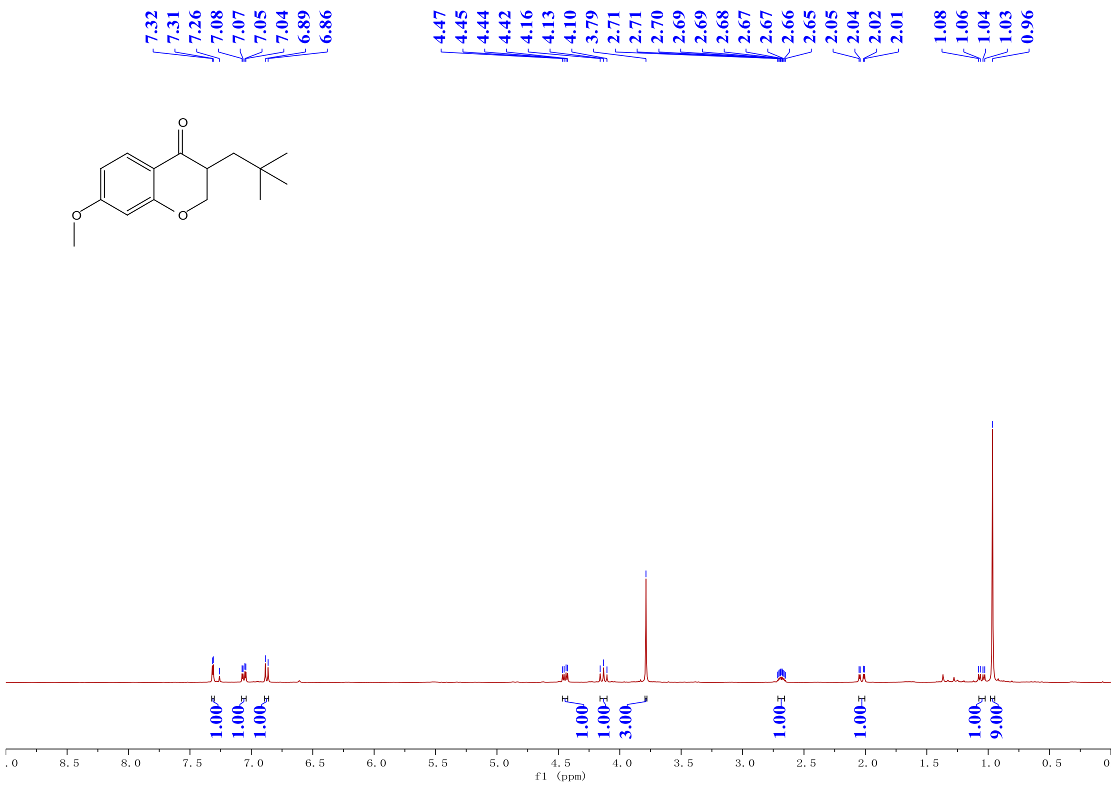

^{1}H NMR spectrum of **6fa** (400 MHz, $CDCl_3$)

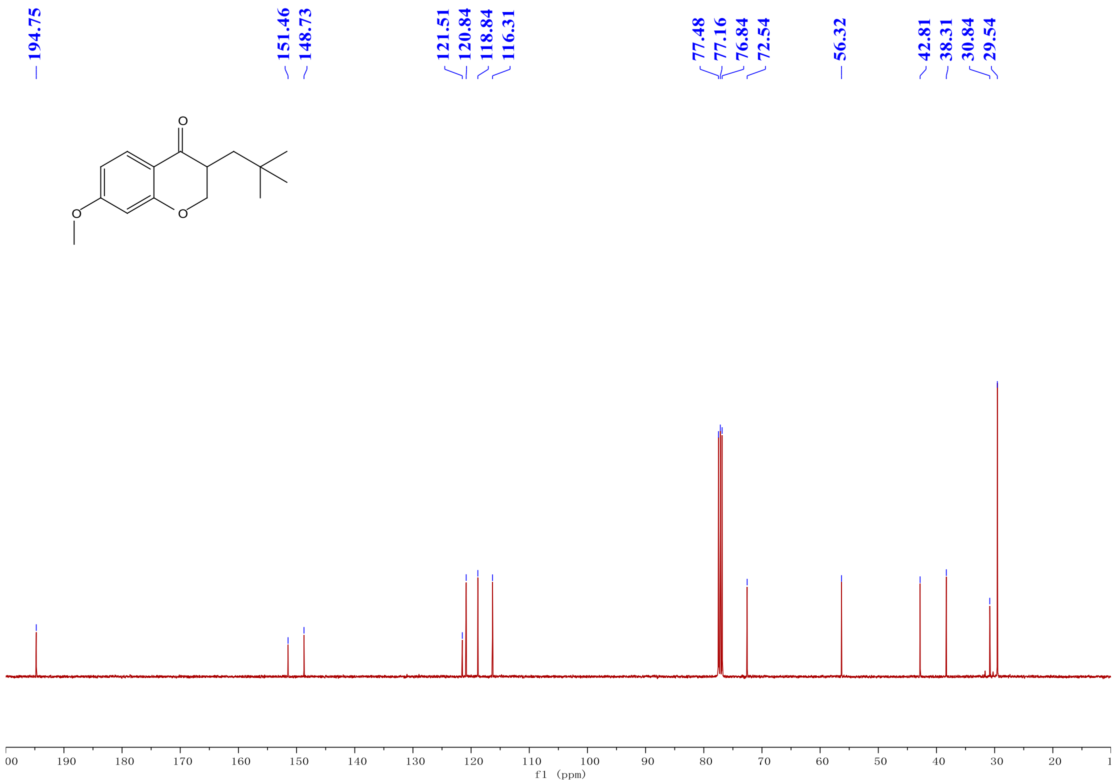

^{13}C {^{1}H} NMR spectrum of Compound **6fa** (101 MHz, $CDCl_3$)

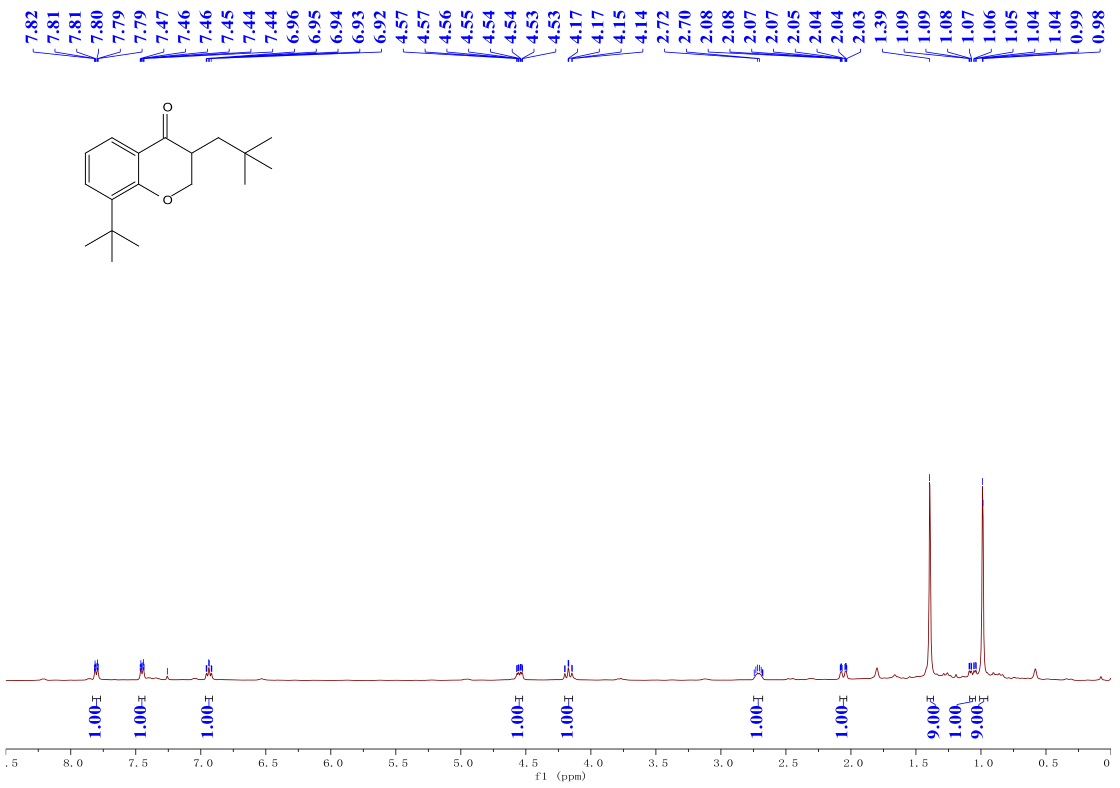

^{1}H NMR spectrum of **6ga** (400 MHz, $CDCl_3$)

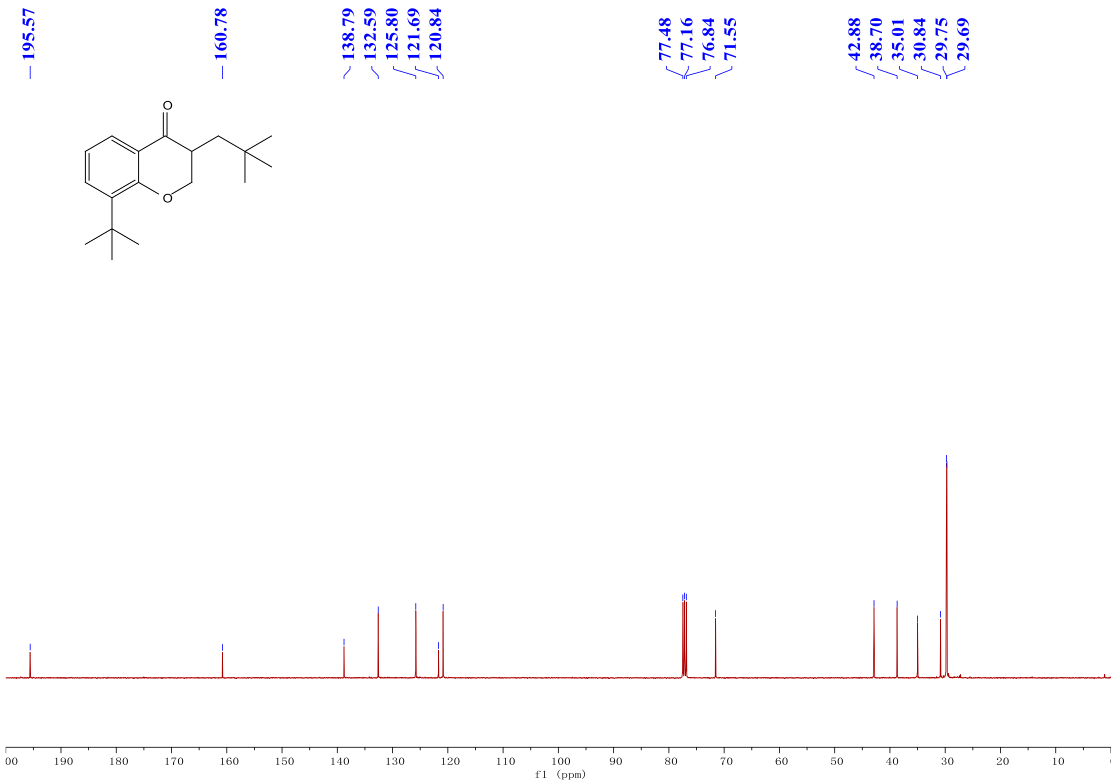

^{13}C {^{1}H} NMR spectrum of Compound **6ga** (101 MHz, $CDCl_3$)

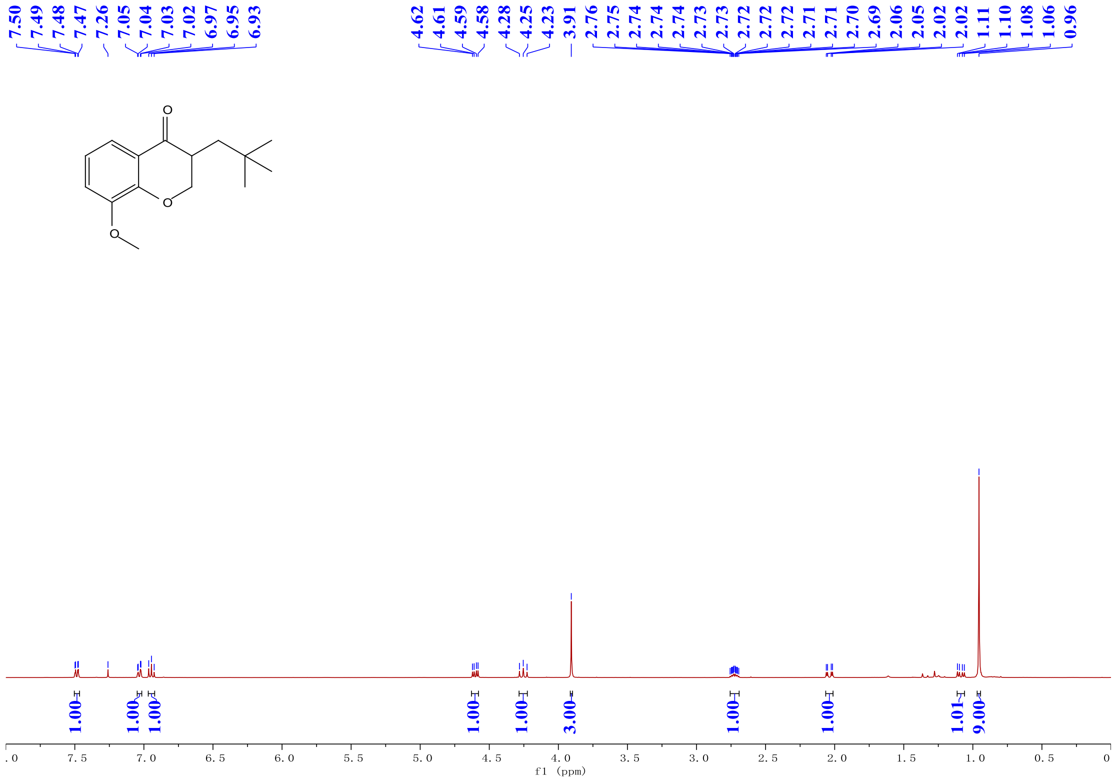

^{1}H NMR spectrum of **6ha** (400 MHz, $CDCl_3$)

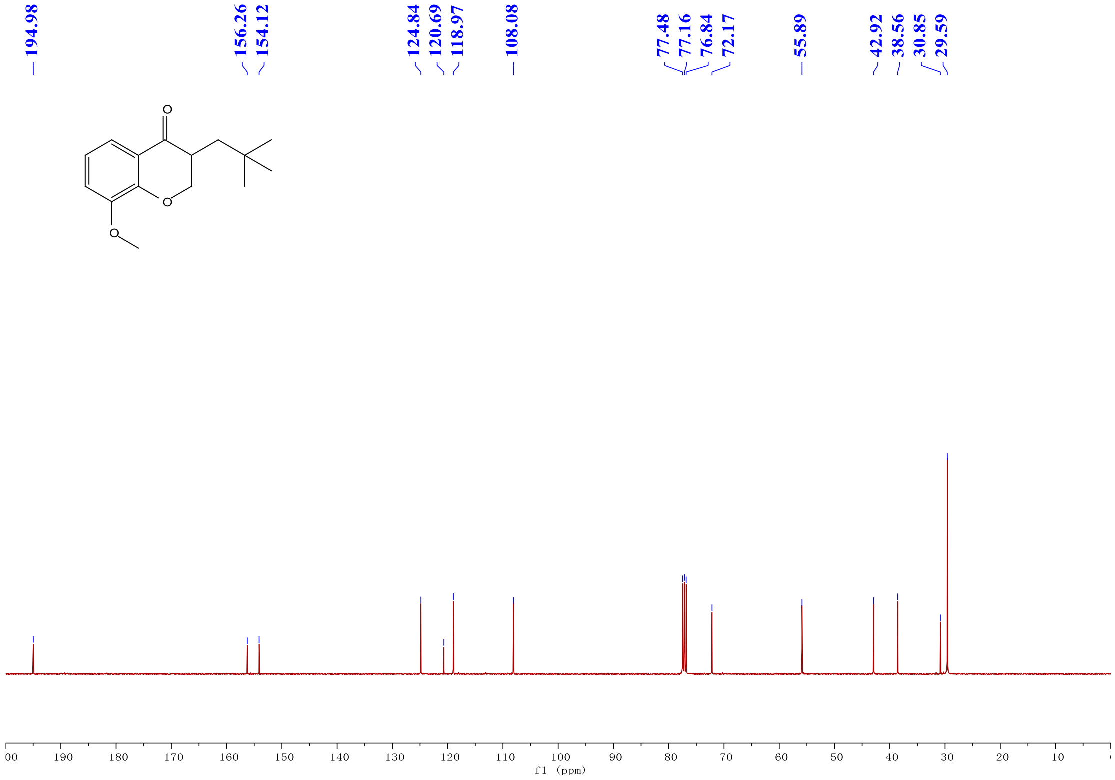

^{13}C {^{1}H} NMR spectrum of Compound **6ha** (101 MHz, $CDCl_3$)

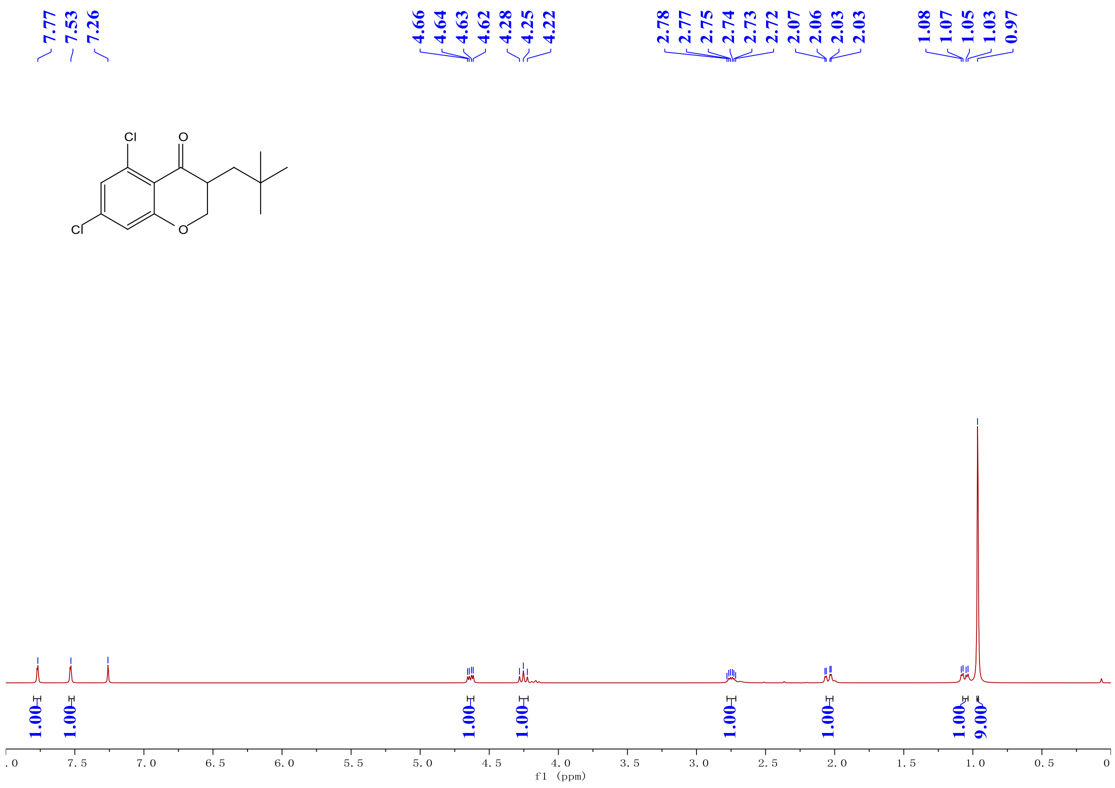

^{1}H NMR spectrum of **6ia** (400 MHz, $CDCl_3$)

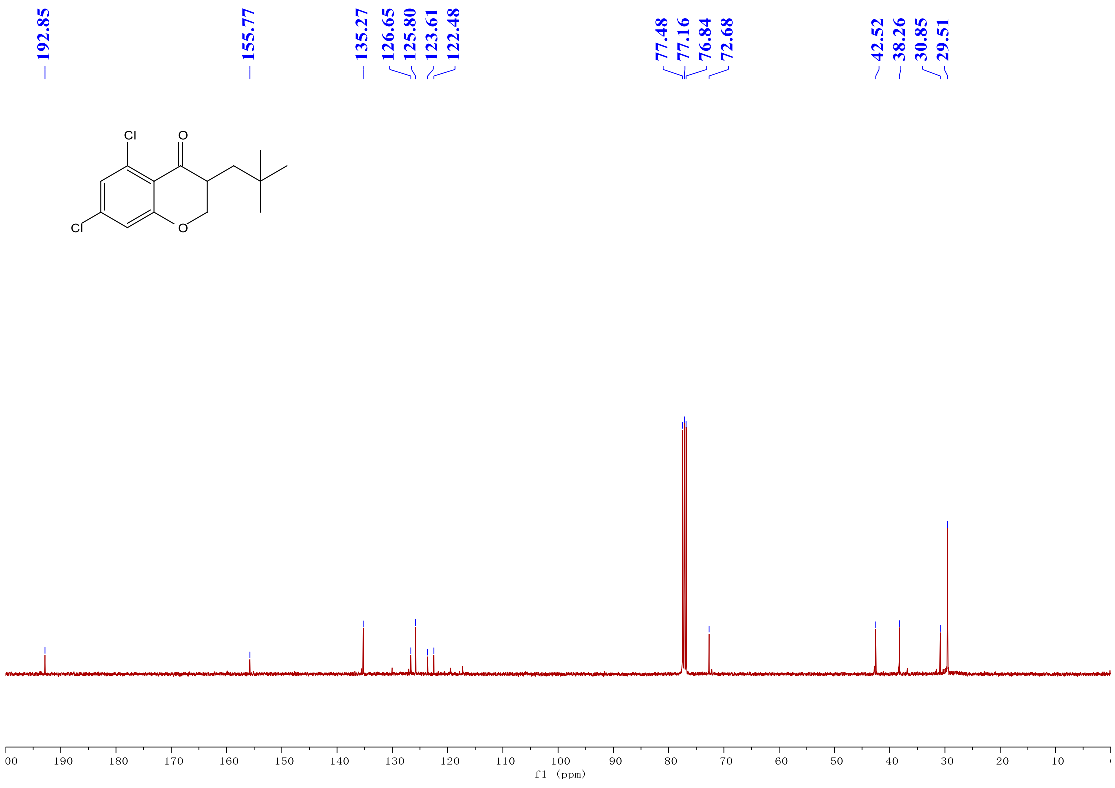

^{13}C {^{1}H} NMR spectrum of Compound **6ia** (101 MHz, $CDCl_3$)

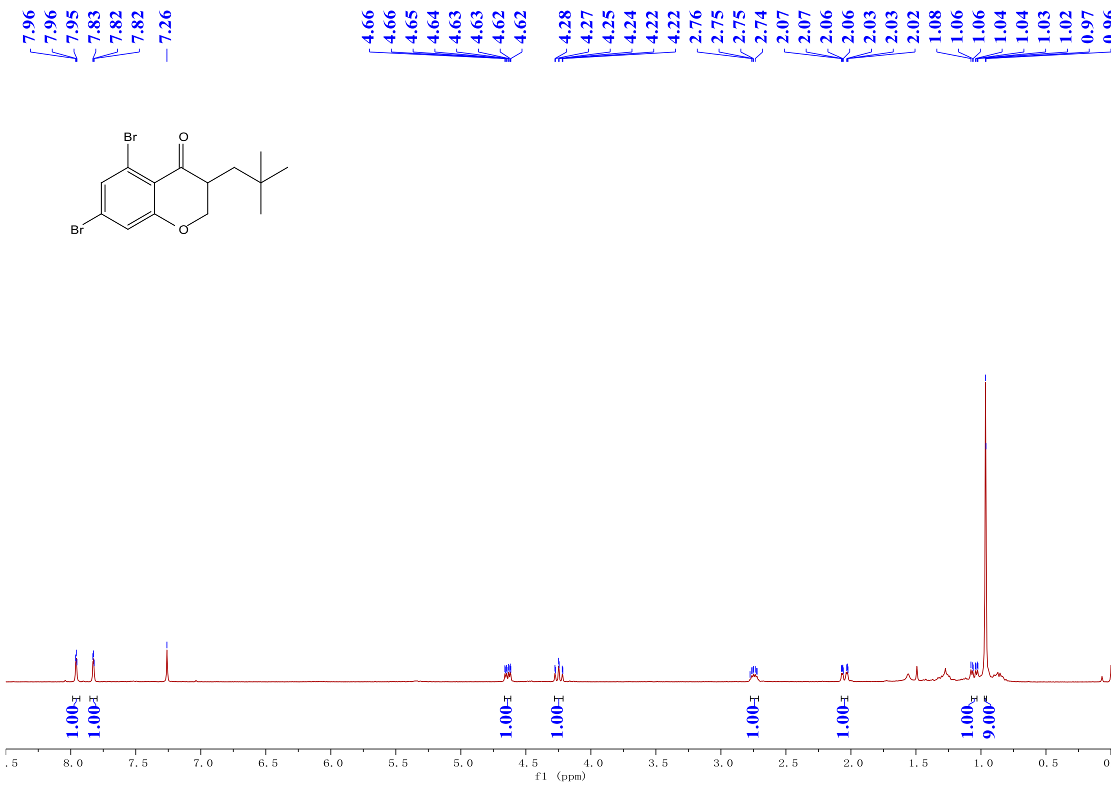

^{1}H NMR spectrum of **6ja** (400 MHz, $CDCl_3$)

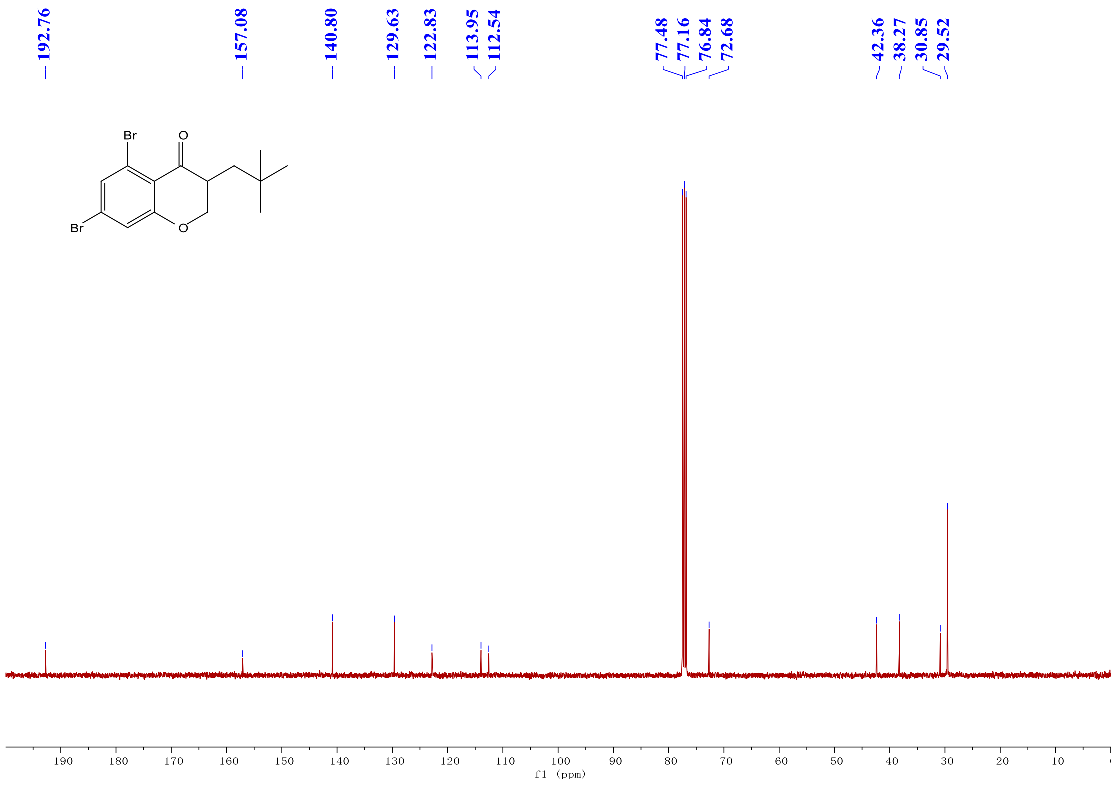

^{13}C {^{1}H} NMR spectrum of Compound **6ja** (101 MHz, $CDCl_3$)

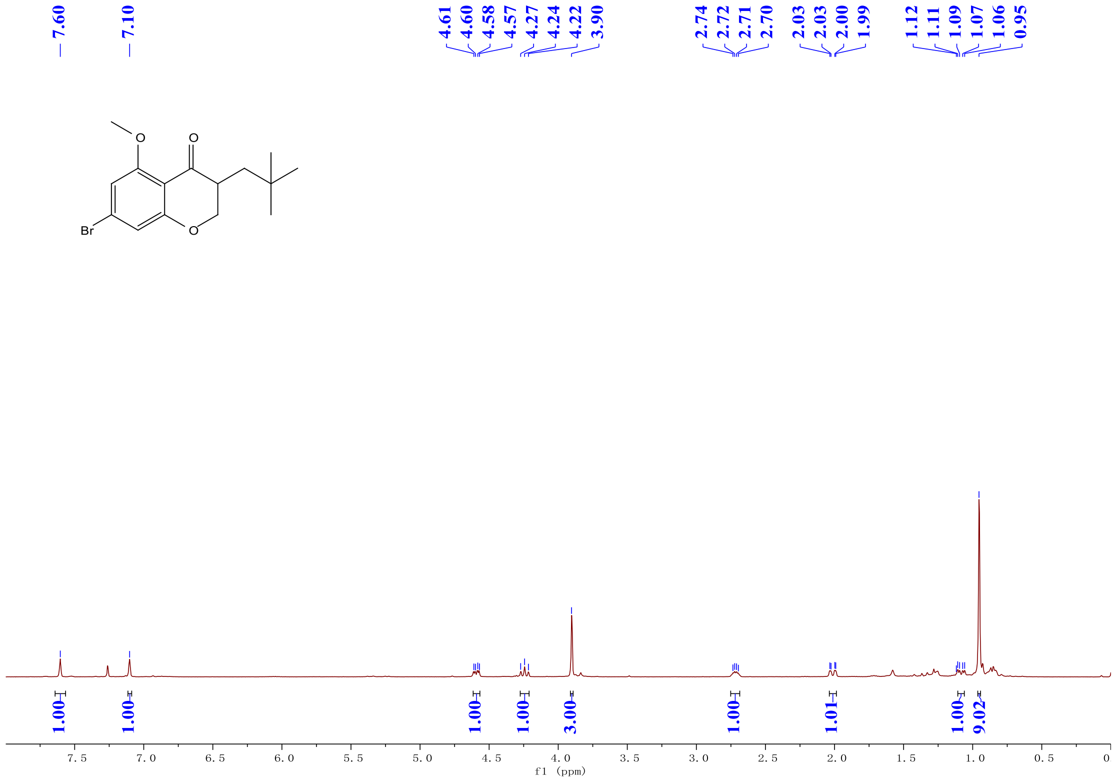

^{1}H NMR spectrum of **6ka** (400 MHz, $CDCl_3$)

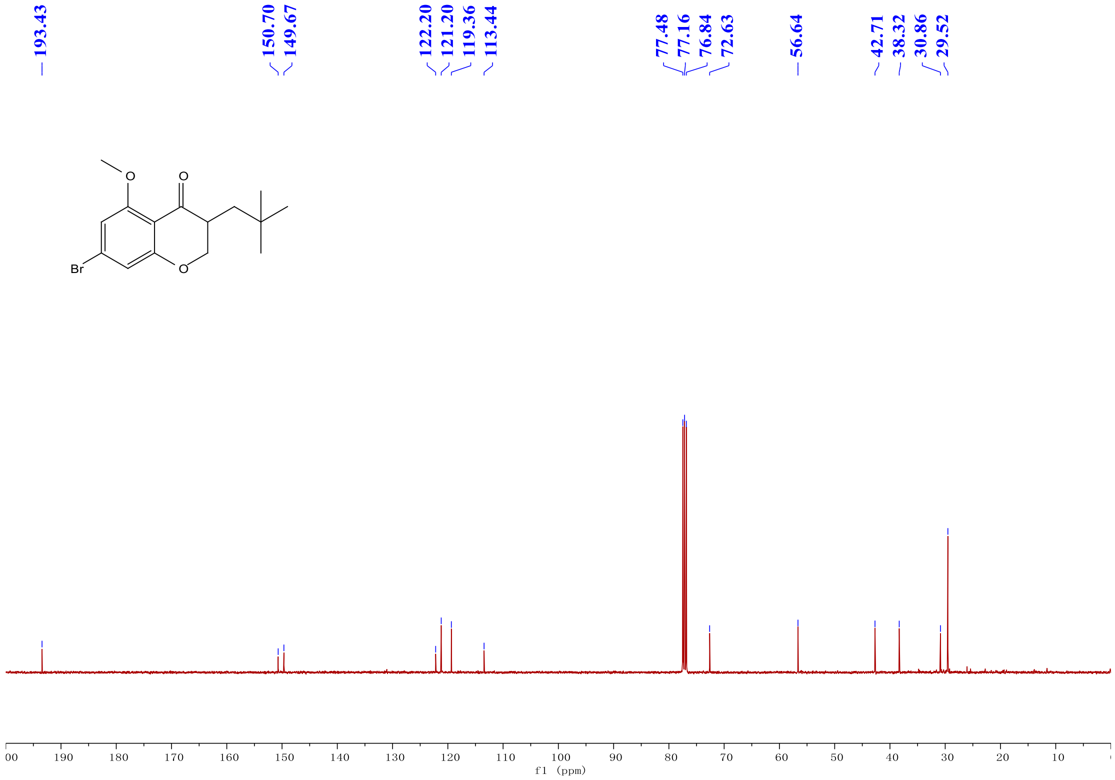

^{13}C {^{1}H} NMR spectrum of Compound **6ka** (101 MHz, $CDCl_3$)

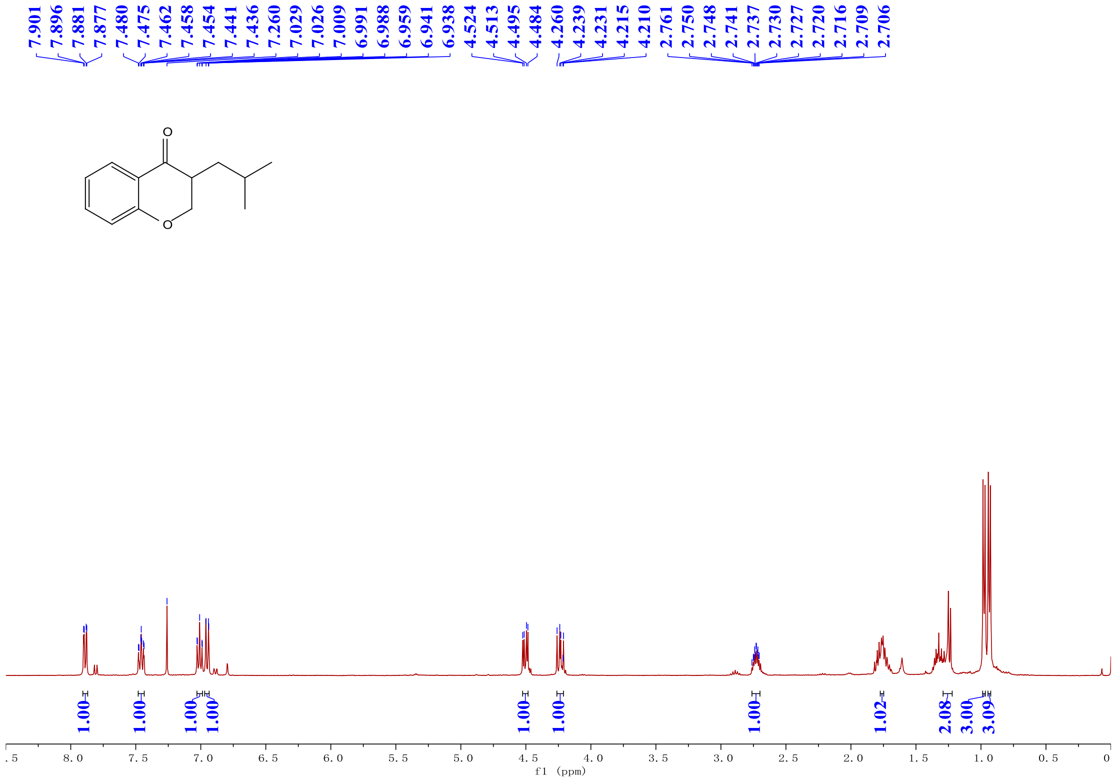

^1H NMR spectrum of **6ab** (400 MHz, $CDCl_3$)

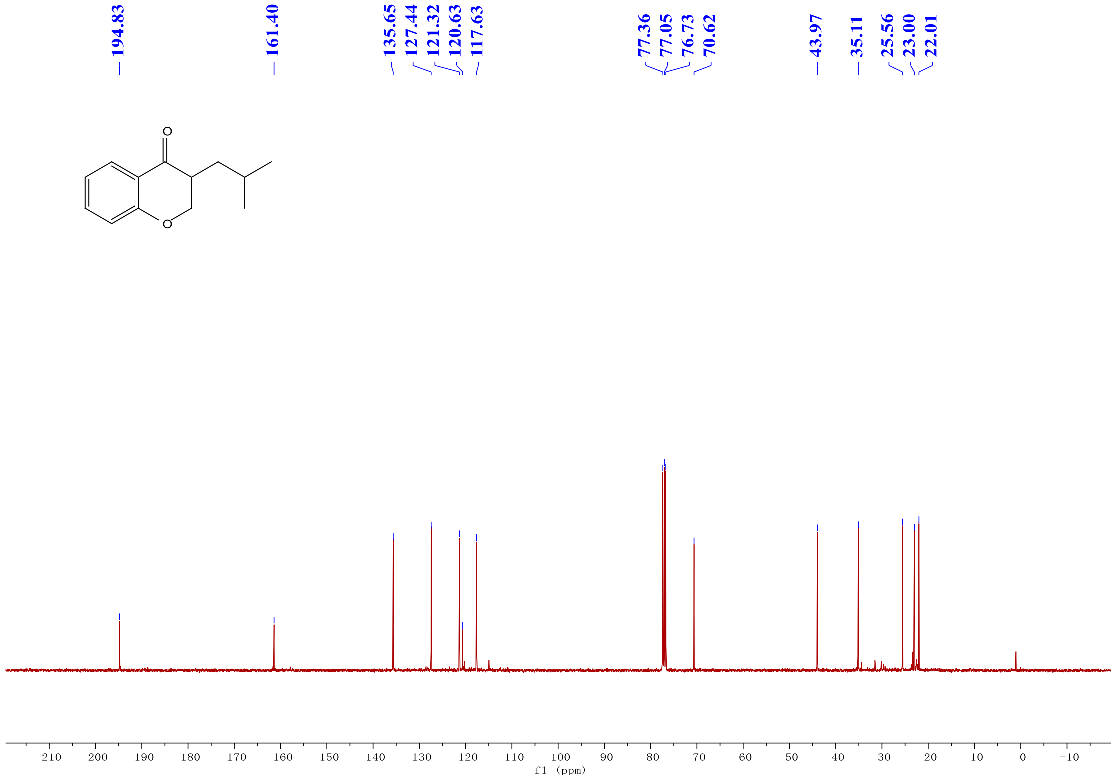

^{13}C {^1H} NMR spectrum of Compound **6ab** (101 MHz, $CDCl_3$)

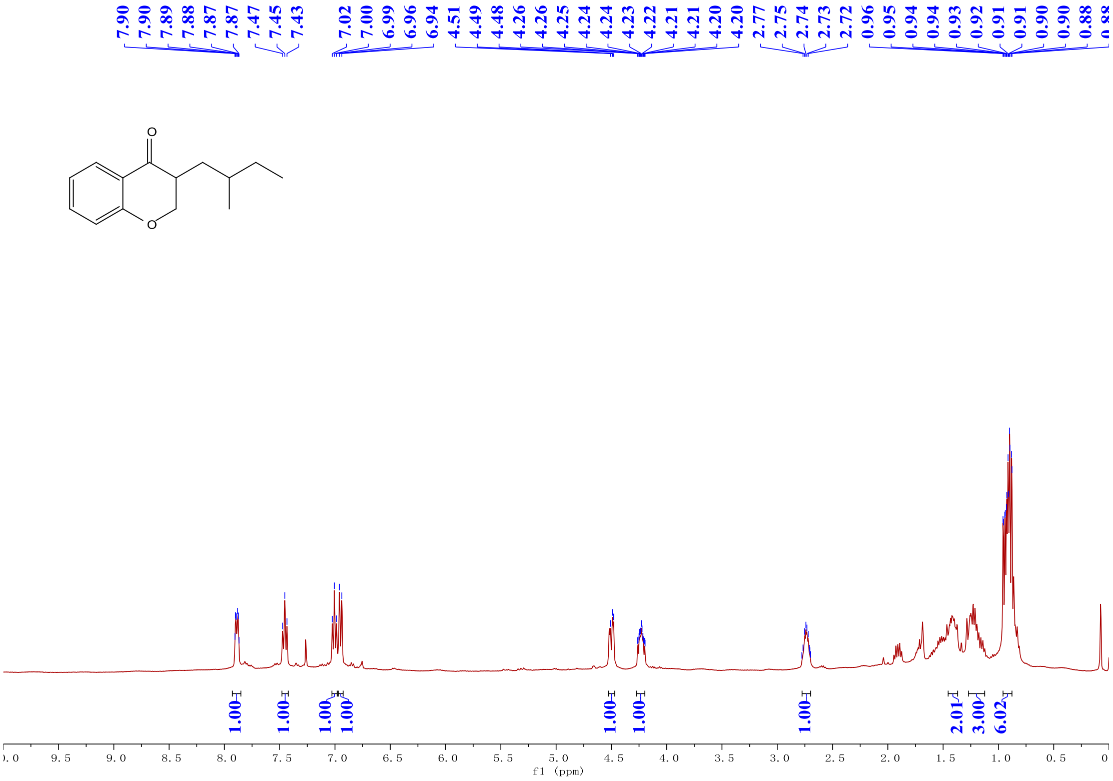

^{1}H NMR spectrum of **6ac** (400 MHz, $CDCl_3$)

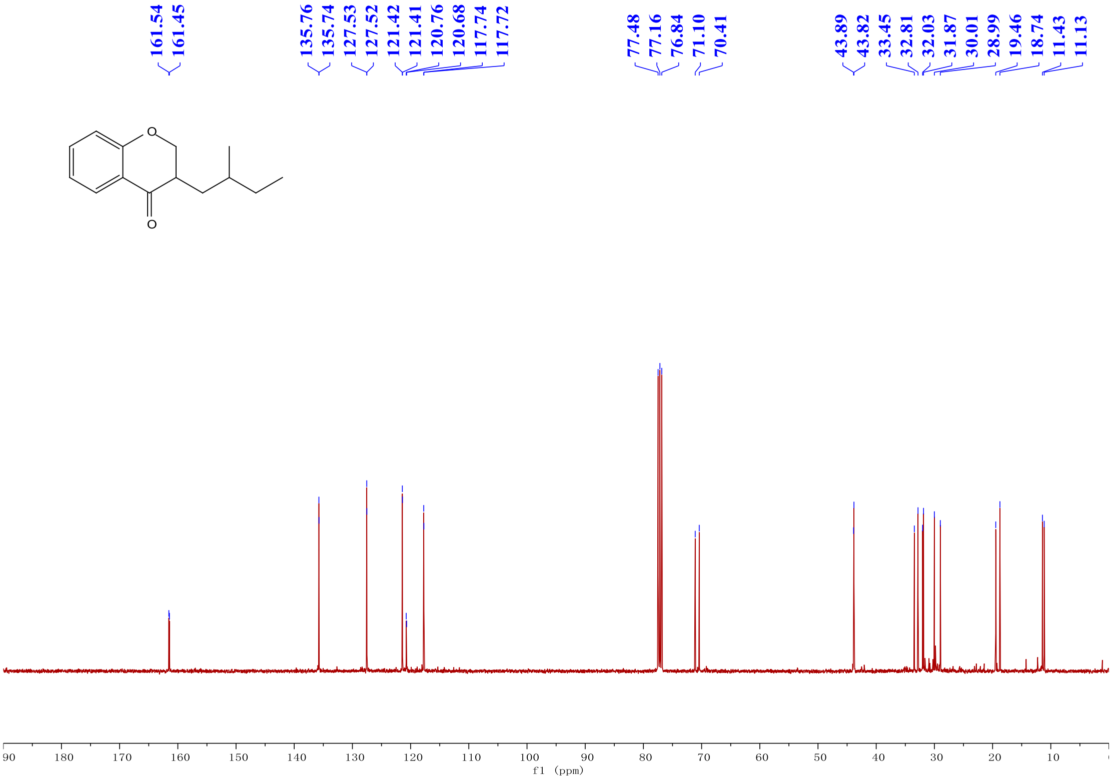

^{13}C {^{1}H} NMR spectrum of Compound **6ac** (101 MHz, $CDCl_3$)

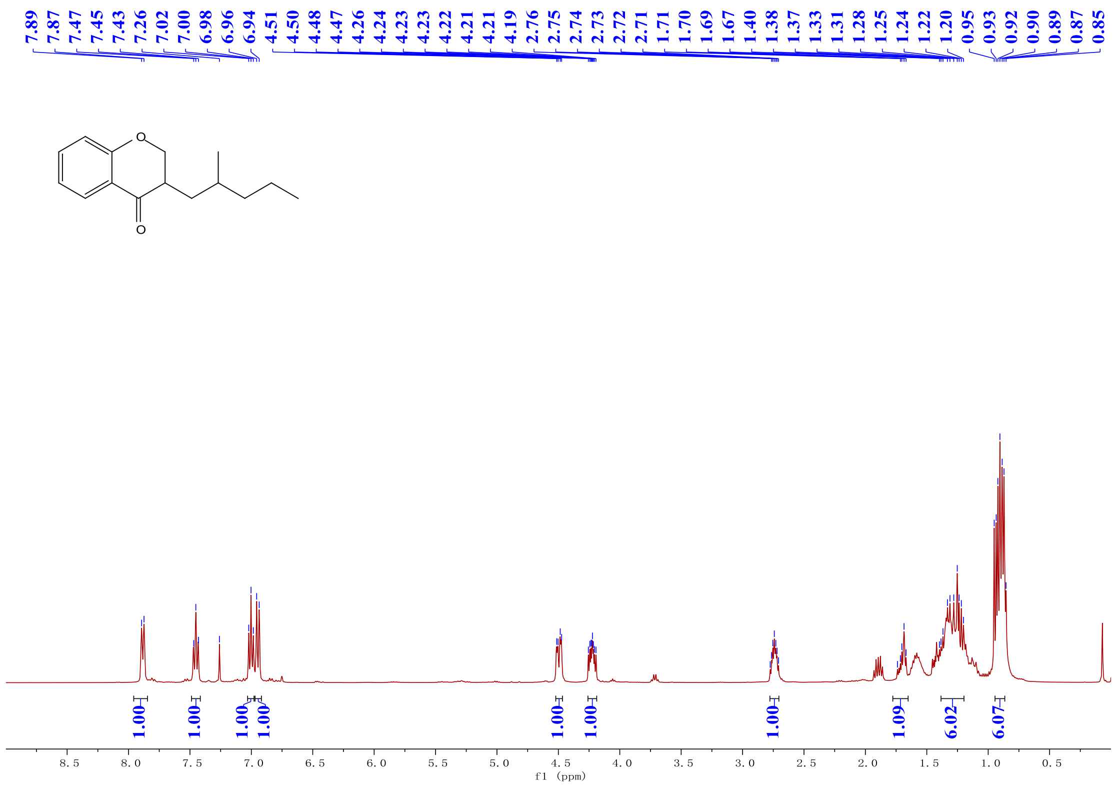

^{1}H NMR spectrum of **6ad** (400 MHz, $CDCl_3$)

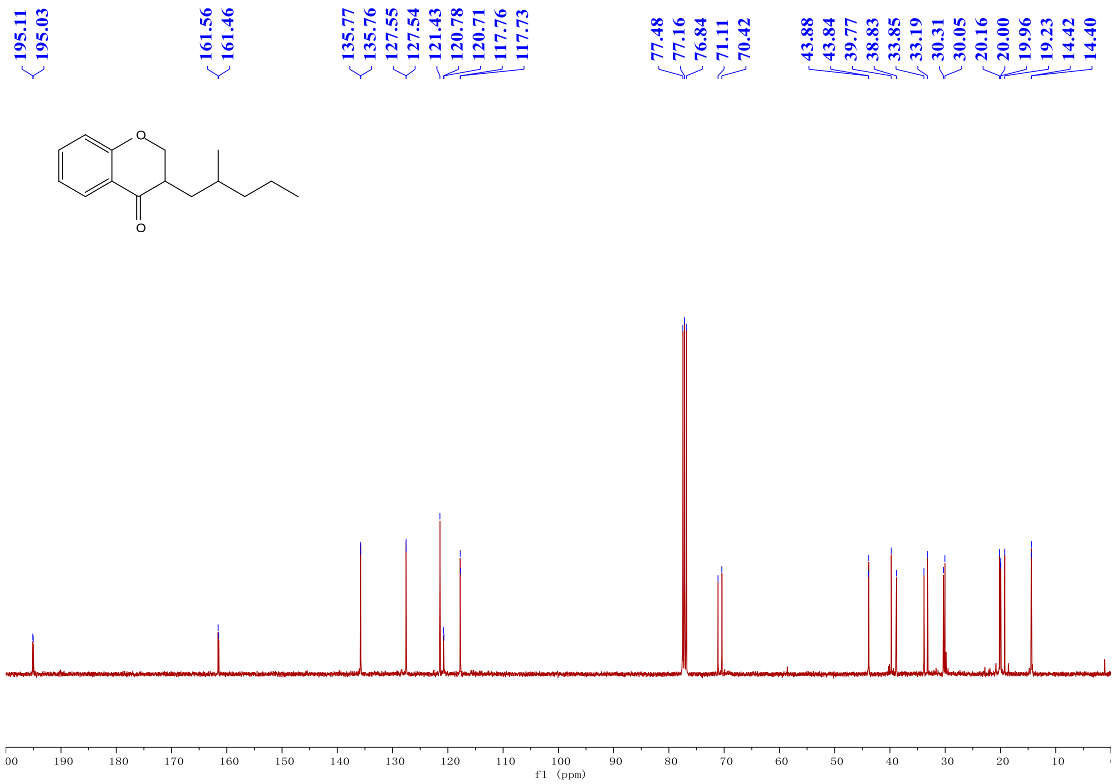

^{13}C {^{1}H} NMR spectrum of Compound **6ad** (101 MHz, $CDCl_3$)

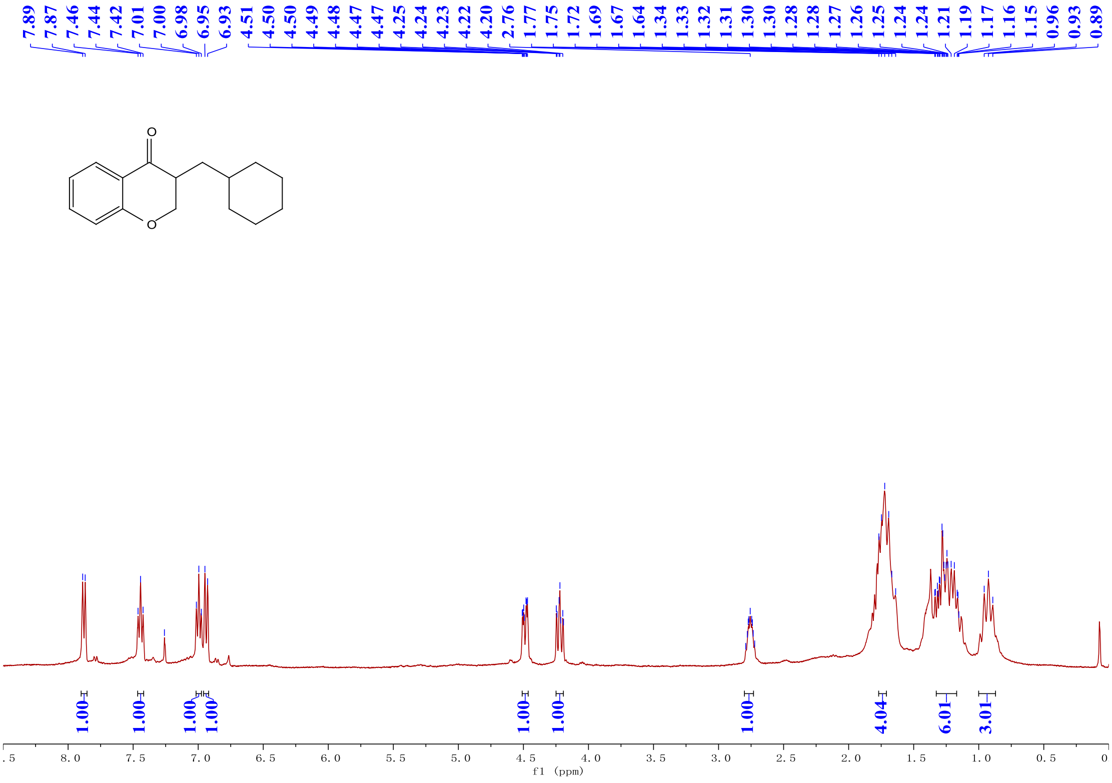

^{1}H NMR spectrum of **6ae** (400 MHz, $CDCl_3$)

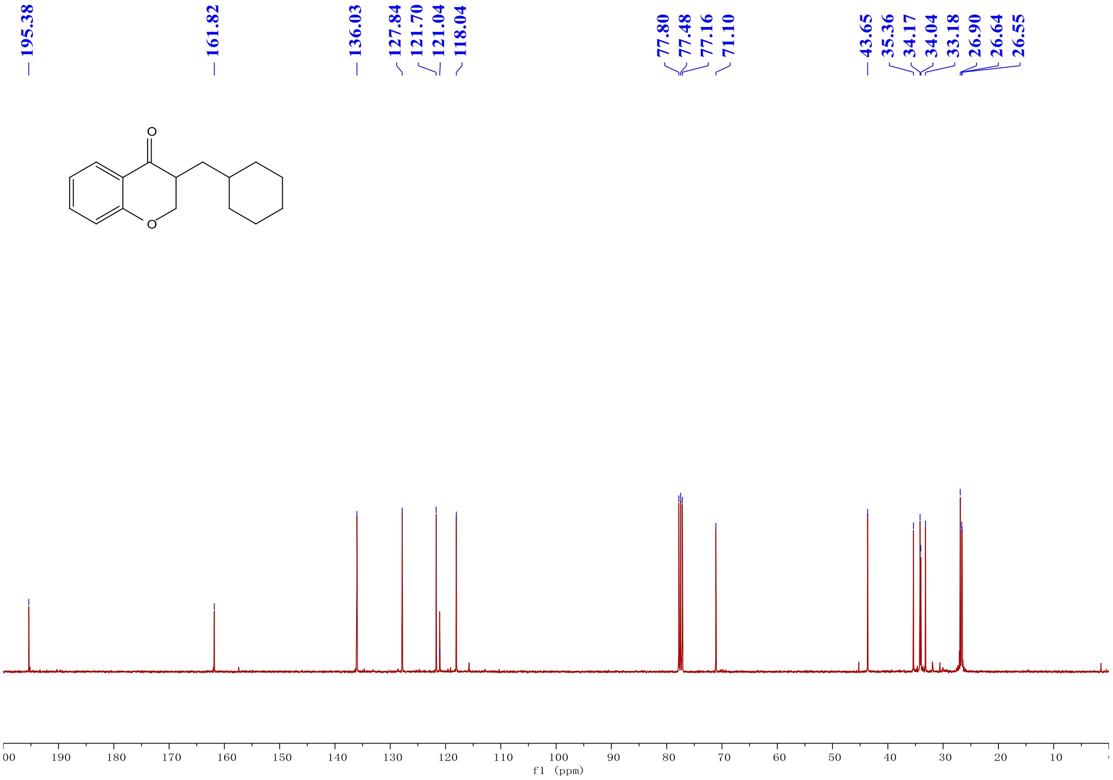

$^{13}C\ \{^{1}H\}$ NMR spectrum of Compound **6ae** (101 MHz, $CDCl_3$)

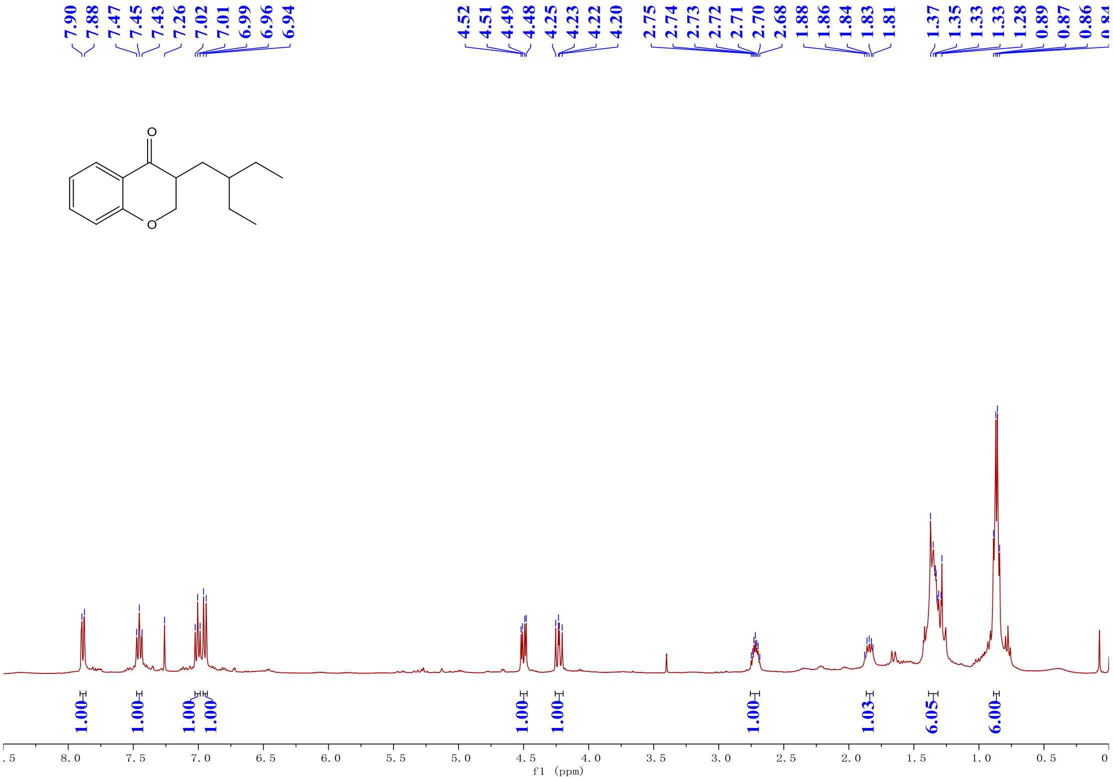

^1H NMR spectrum of **6af** (400 MHz, $CDCl_3$)

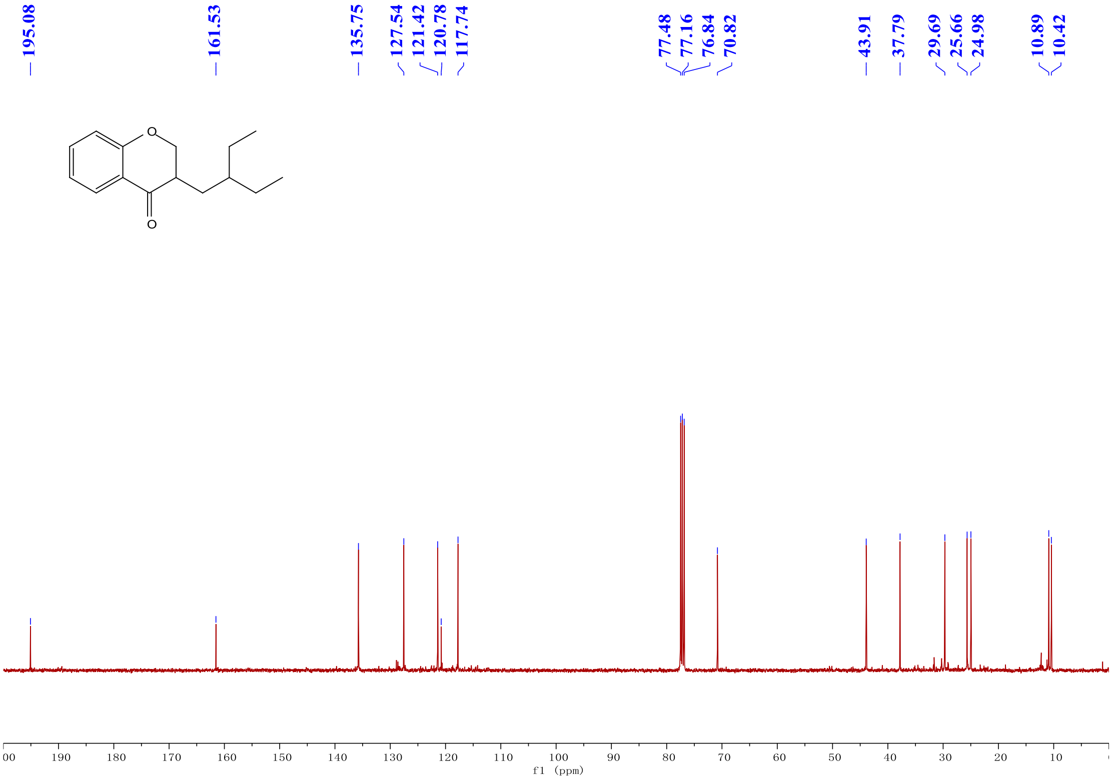

^{13}C {^1H} NMR spectrum of Compound **6af** (101 MHz, $CDCl_3$)

12. HRMS Spectra

7-methoxy-1,3-dimethyl-3-neopentylindolin-2-one (3ca):

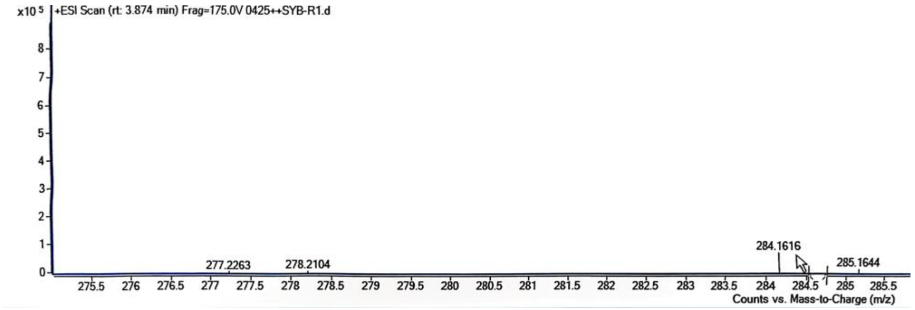

HRMS (ESI/Q-TOF) m/z: $[M+Na]^+$ calcd. for $C_{16}H_{23}NNaO_2^+$ 284.1621, found 284.1616.

7-fluoro-1,3-dimethyl-3-neopentylindolin-2-one (3da):

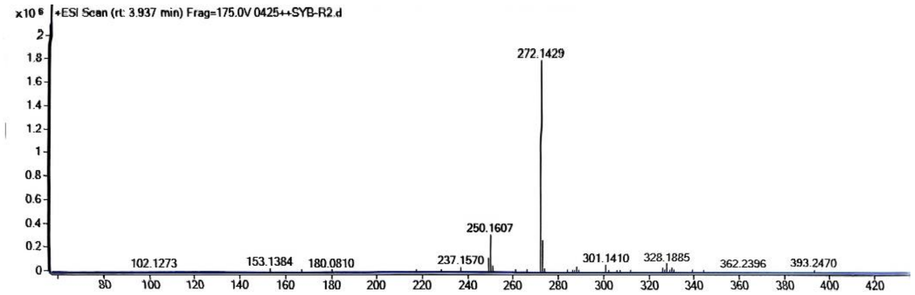

HRMS (ESI/Q-TOF) m/z: $[M+Na]^+$ calcd. for $C_{15}H_{20}FNNaO^+$ 272.1422, found 272.1429.

ethyl 1,3-dimethyl-3-neopentyl-2-oxoindoline-7-carboxylate (**3ga**):

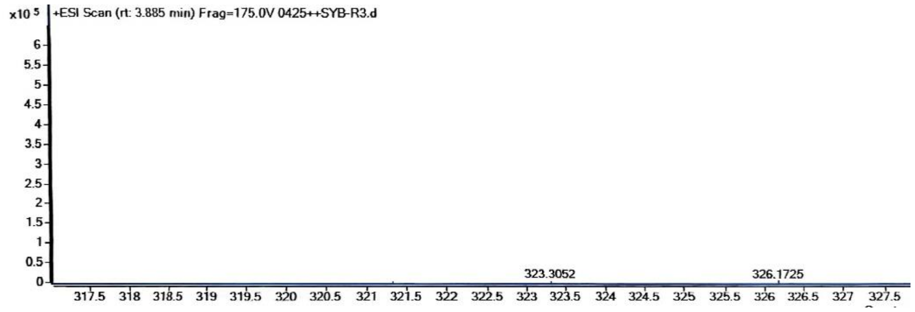

HRMS (ESI/Q-TOF) m/z: $[M+Na]^+$ calcd. for $C_{18}H_{25}NNaO_3^+$ 326.1727, found 326.1725.

4-methoxy-1,3-dimethyl-2-methylene-3-neopentylindoline (3qa):

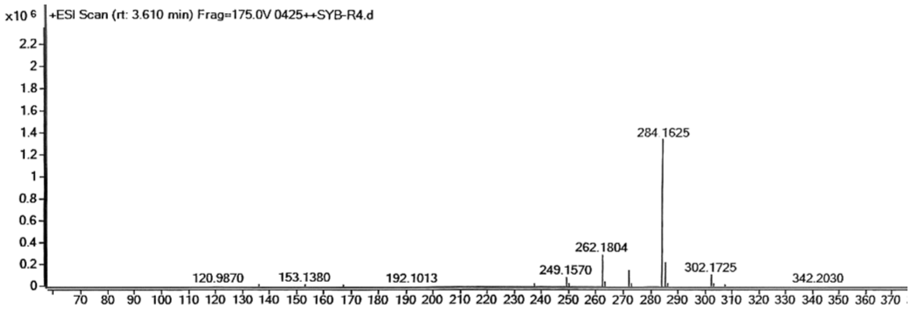

HRMS (ESI/Q-TOF) m/z: $[M+Na]^+$ calcd. for $C_{16}H_{23}NNaO_2^+$ 284.1621, found 284.1625.

6-bromo-1,3-dimethyl-3-neopentylindolin-2-one (3sa):

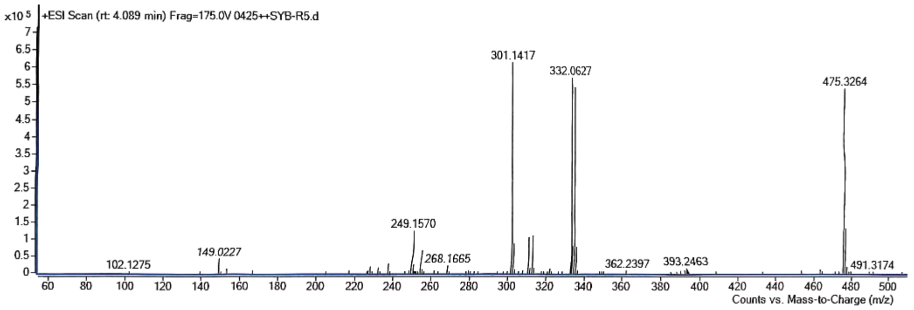

HRMS (ESI/Q-TOF) m/z: $[M+Na]^+$ calcd. for $C_{15}H_{20}BrNNaO^+$ 332.0620, found 332.0627.

3,5-dimethyl-3-neopentyl-1-(*p*-tolyl)indolin-2-one (3xa):

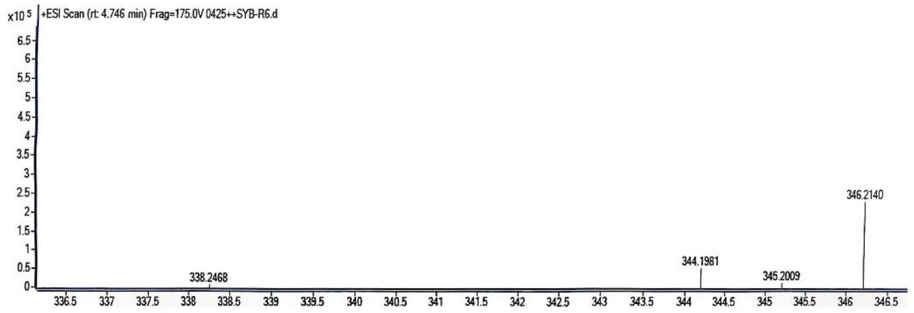

HRMS (ESI/Q-TOF) m/z: $[M+Na]^+$ calcd. for $C_{22}H_{27}NNaO^+$ 344.1985, found 344.1981.

3-(3,3-dimethylbutyl)-1-ethyl-3-methylindolin-2-one (3ai):

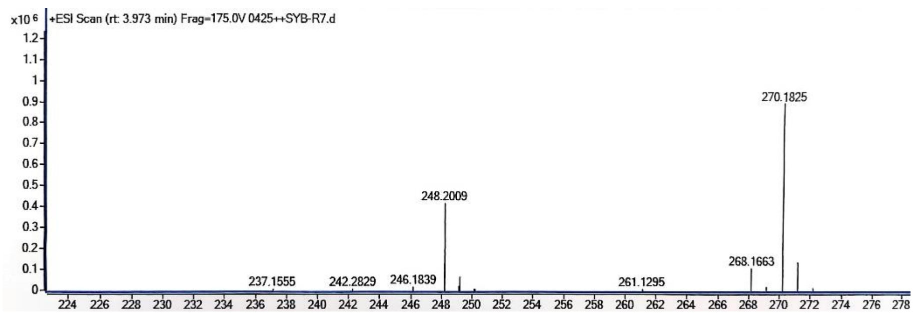

HRMS (ESI/Q-TOF) m/z: [M+Na]$^+$ calcd. for $C_{22}H_{23}NNaO^+$ 268.1672, found 268.1663.

7-methyl-3-neopentylchroman-4-one (6ea):

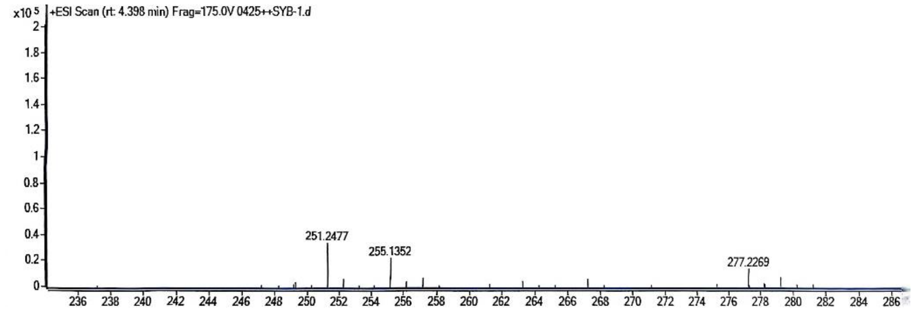

HRMS (ESI/Q-TOF) m/z: [M+Na]$^+$ calcd. for $C_{15}H_{20}NaO_2^+$ 255.1356, found 255.1352.

8-(*tert*-butyl)-3-neopentylchroman-4-one (6ga):

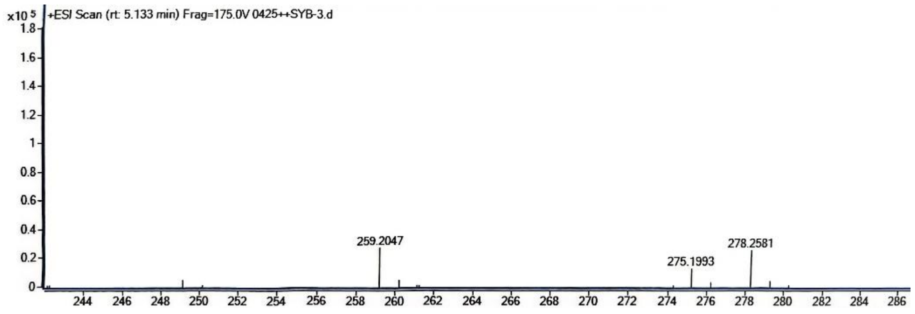

HRMS (ESI/Q-TOF) m/z: [M+H]$^+$ calcd. for $C_{18}H_{27}O_2^+$ 275.2006, found 275.1993.

8-methoxy-3-neopentylchroman-4-one (6ha):

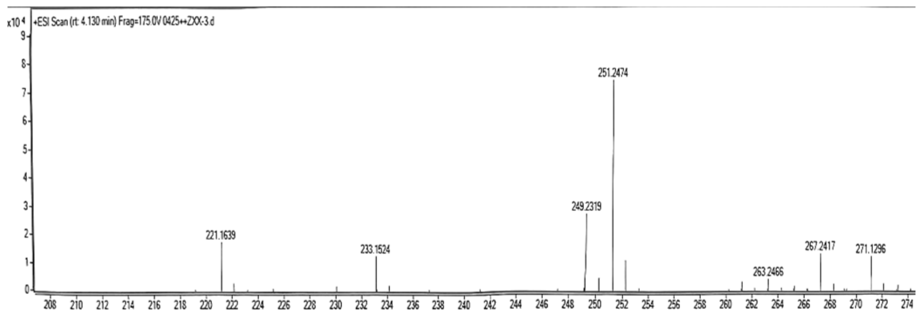

HRMS (ESI/Q-TOF) m/z: [M+Na]+ calcd. for $C_{15}H_{20}NaO_3^+$ 271.1305, found 271.1296.

6,8-dibromo-3-neopentylchroman-4-one (6ja):

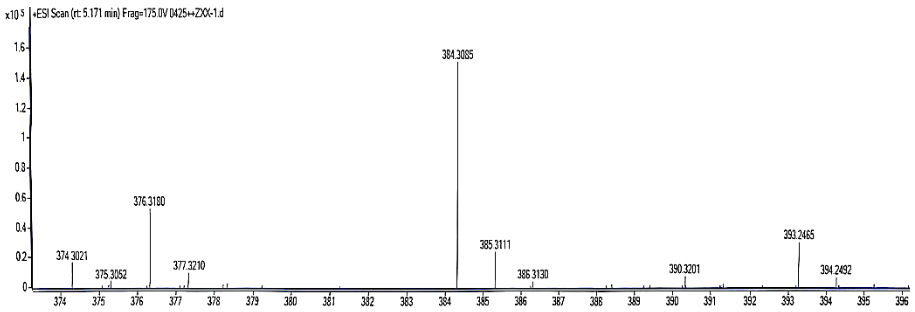

HRMS (ESI/Q-TOF) m/z: [M+H]+ calcd. for $C_{14}H_{17}Br_2O_2^+$ 376.3175, found 376.3180.

6-bromo-8-methoxy-3-neopentylchroman-4-one (6ka):

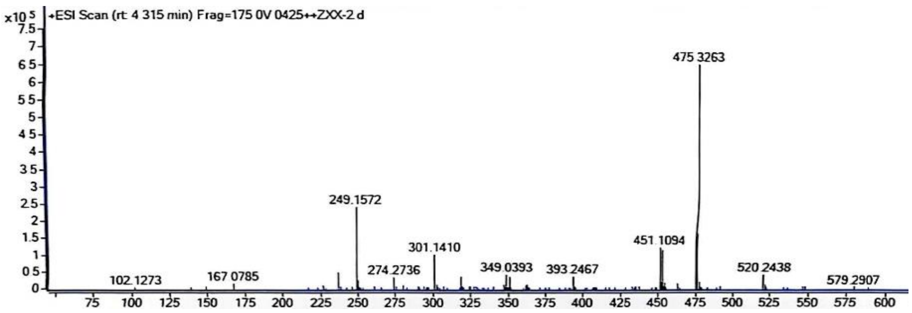

HRMS (ESI/Q-TOF) m/z: [M+Na]+ calcd. for $C_{15}H_{19}BrNaO_3^+$ 349.0410, found 349.0393.

3-isobutylchroman-4-one (6ab):

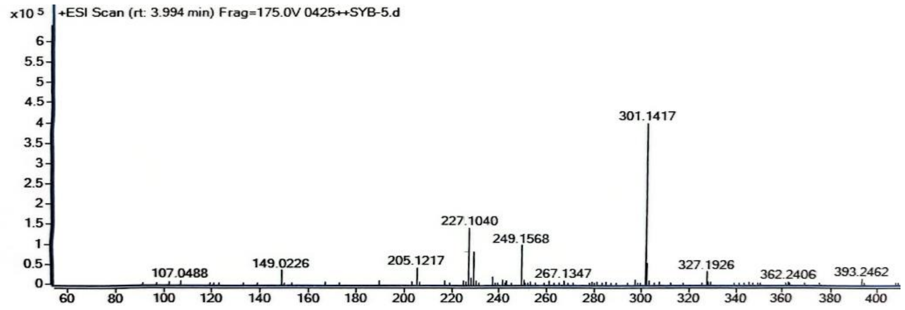

HRMS (ESI/Q-TOF) m/z: $[M+Na]^+$ calcd. for $C_{13}H_{16}NaO_2^+$ 227.1043, found 227.1040.

3-(2-methylbutyl)chroman-4-one (6ac):

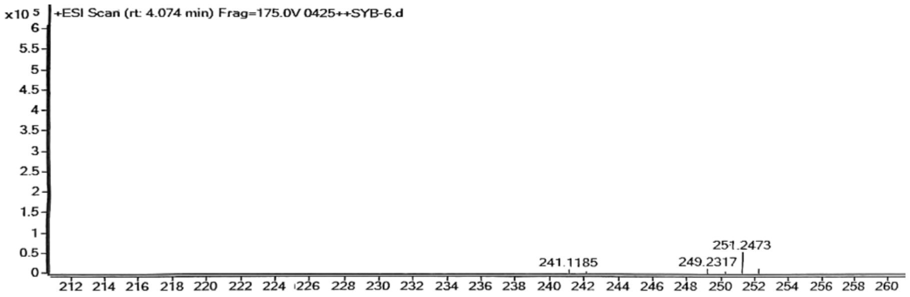

HRMS (ESI/Q-TOF) m/z: $[M+Na]^+$ calcd. for $C_{14}H_{18}NaO_2^+$ 241.1199, found 227.1185.

3-(2-methylpentyl)chroman-4-one (6ad):

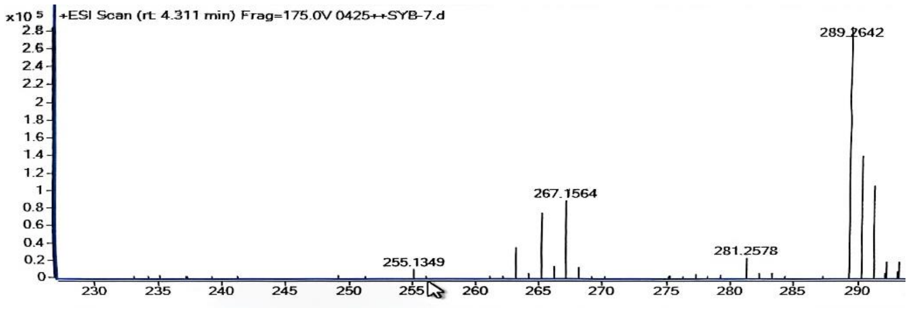

HRMS (ESI/Q-TOF) m/z: $[M+Na]^+$ calcd. for $C_{15}H_{20}NaO_2^+$ 255.1356, found 255.1349.

3-(cyclohexylmethyl)chroman-4-one (6ae):

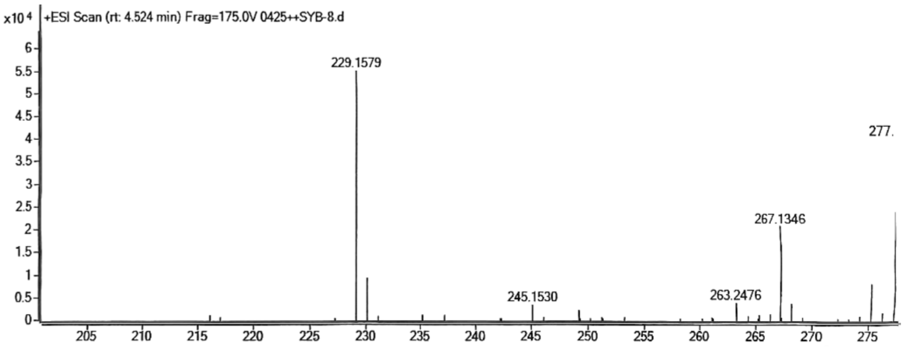

HRMS (ESI/Q-TOF) m/z: $[M+H]^+$ calcd. for $C_{15}H_{21}O_2^+$ 245.1537, found 245.1530.

3-(2-ethylbutyl)chroman-4-one (6af):

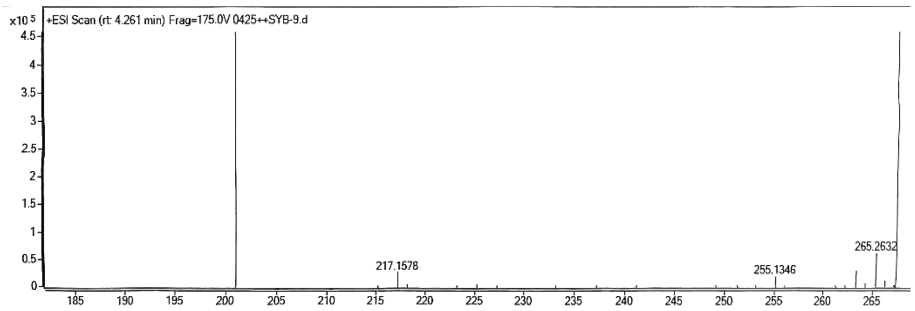

HRMS (ESI/Q-TOF) m/z: $[M+Na]^+$ calcd. for $C_{15}H_{20}NaO_2^+$ 255.1356, found 255.1346.
